# Supplementary material for: Cerebral small vessel disease genomics and its implications across the lifespan
Source: Nat Commun. 2020 Dec 8;11:6285. doi: 10.1038/s41467-020-19111-2 (PMC7722866; doi:10.1038/s41467-020-19111-2)
Supplement: Supplementary file 1 — Supplementary information [file 41467_2020_19111_MOESM1_ESM.pdf]

Supplementary note

This project is an EU Joint Programme -Neurodegenerative Disease Research (JPND) project. The project is supported through the following funding organisations under the aegis of JPND- www.jpnd.eu: Australia, National Health and Medical Research Council, Austria, Federal Ministry of Science, Research and Economy; Canada, Canadian Institutes of Health Research; France, French National Research Agency; Germany, Federal Ministry of Education and Research; Netherlands, The Netherlands Organisation for Health Research and Development; United Kingdom, Medical Research Council. This project has received funding from the European Union's Horizon 2020 research and innovation programme under grant agreement No 643417. This project has also received funding from the European Research Council (ERC) under the European Union's Horizon 2020 research and innovation programme under grant agreement No 640643 and from the European Union's Horizon 2020 research and innovation programme under grant agreements No 667375 and 754517. This work was also supported by a grant overseen by the French National Research Agency (ANR) as part of the "Investment for the Future Programme" ANR-18-RHUS-0002. Part of the computations were performed at the Bordeaux Bioinformatics Center (CBiB), University of Bordeaux and at the CREDIM (Centre de Ressource et Développement en Informatique Médicale) at University of Bordeaux, on a server infrastructure supported by the Fondation Claude Pompidou. We would like to thank Prof. Dr. Marco Düring, Institute for Stroke and Dementia Research, Klinikum der Universität München, for his valuable suggestions in defining and estimating the diffusion-tensor imaging metrics for the i-Share student cohort.

AGES: Age, Gene/Environment Susceptibility (AGES) –Reykjavik. The study was funded by the National Institute on Aging (NIA) (N01-AG-12100), Hjartavernd (the Icelandic Heart Association), and the Althingi (the Icelandic Parliament), with contributions from the Intramural Research Programs at the NIA and at the National Heart, Lung, and Blood Institute (Z01 HL004607-08 CE). The study was approved by the Icelandic National Bioethics Committee (VSN: 00-063) and the MedStarResearch Institute (project 2003-145).

ARIC: The Atherosclerosis Risk in Communities study has been funded in whole or in part with Federal funds from the National Heart, Lung, and Blood Institute, National Institutes of Health, Department of Health and Human Services, under Contract nos. (HHSN268201700001I, HHSN268201700002I, HHSN268201700003I, HHSN268201700005I, HHSN268201700004I). The authors thank the staff and participants of the ARIC study for their important contributions. Funding support for "Building on GWAS for NHLBI-diseases: the U.S. CHARGE consortium" was provided by the NIH through the American Recovery and Reinvestment Act of 2009 (ARRA) (5RC2HL102419). This project was funded from R01-NS087541 to Myriam Fornage and Eric Boerwinkle.

ASPS/ASPSFam: The authors thank the staff and the participants for their valuable contributions. We thank Birgit Reinhart for her long-term administrative commitment, Elfi Hofer for the technical assistance at creating the DNA bank, Ing. Johann Semmler and Anita Harb for DNA sequencing and DNA analyses by TaqMan assays and Irmgard Poelzl for supervising the quality management processes after ISO9001 at the biobanking and DNA analyses.

The Medical University of Graz and the Steiermärkische Krankenanstaltengesellschaft support the databank of the ASPS/ASPS-Fam. The research reported in this article was funded by the Austrian Science Fund (FWF) grant numbers PI904, P20545-B05 and P13180 and supported by the Austrian National Bank Anniversary Fund, P15435 and the Austrian Ministry of Science under the aegis of the EU Joint Programme-Neurodegenerative Disease Research (JPND)-www.jpnd.eu.**CHS:**

This CHS research was supported by NHLBI contracts HHSN268201200036C, HHSN268200800007C, HHSN268201800001C, N01HC55222, N01HC85079, N01HC85080, N01HC85081, N01HC85082, N01HC85083, N01HC85086, N01HC15103, HHSN268200960009C; and NHLBI grants U01HL080295, R01HL087652, R01HL105756, R01HL103612, R01HL120393, R01HL085251 and U01HL130114 with additional contribution from the National Institute of Neurological Disorders and Stroke (NINDS). Additional support was provided through R01AG023629, R01AG033193 from the National Institute on Aging (NIA). A full list of principal CHS investigators and institutions can be found at CHS-NHLBI.org.

The provision of genotyping data was supported in part by the National Center for Advancing Translational Sciences, CTSI grant UL1TR001881, and the National Institute of Diabetes and Digestive and Kidney Disease Diabetes Research Center (DRC) grant DK063491 to the Southern California Diabetes Endocrinology Research Center.

CHAP: This study has been funded by NIH grant R01-AG-09966 and R01-AG-11101 for the parent CHAP study. The genetic analysis was supported by NIH grant R01-AG-030146. This study was approved by Rush University Medical Center Internal Review Board.
CARDIA: The Coronary Artery Risk Development in Young Adults Study (CARDIA) is conducted and supported by the National Heart, Lung, and Blood Institute (NHLBI) in collaboration with the University of Alabama at Birmingham (HHSN268201800005I & HHSN268201800007I), Northwestern University (HHSN268201800003I), University of Minnesota (HHSN268201800006I), and Kaiser Foundation Research Institute (HHSN268201800004I). CARDIA was also partially supported by the Intramural Research Program of the National Institute on Aging (NIA) and an intra-agency agreement between NIA and NHLBI (AG0005). Genotyping was funded as part of the NHLBI Candidate-gene Association Resource (N01-HC-65226) and the NHGRI Gene Environment Association Studies (GENEVA) (U01-HG004729, U01-HG04424, and U01-HG004446). This manuscript has been reviewed and approved by CARDIA for scientific content.
CHS: This CHS research was supported by NHLBI contracts HHSN268201200036C, HHSN268200800007C, HHSN268201800001C, N01HC55222, N01HC85079, N01HC85080, N01HC85081, N01HC85082, N01HC85083, N01HC85086, N01HC15103, HHSN268200960009C; and NHLBI grants U01HL080295, R01HL087652, R01HL105756, R01HL103612, R01HL120393, R01HL085251, and U01HL130114 with additional contribution from the National Institute of Neurological Disorders and Stroke (NINDS). Additional support was provided through R01AG023629 and R01AG033193 from the National Institute on Aging (NIA). A full list of principal CHS investigators and institutions can be found at CHS-NHLBI.org.
The provision of genotyping data was supported in part by the National Center for Advancing Translational Sciences, CTSI grant UL1TR001881, and the National Institute of Diabetes and Digestive and Kidney Disease Diabetes Research Center (DRC) grant DK063491 to the Southern California Diabetes Endocrinology Research Center. The content is solely the responsibility of the authors and does not necessarily represent the official views of the National Institutes of Health.
FHS: This work was supported by the National Heart, Lung and Blood Institute's Framingham Heart Study (Contracts No. N01-HC-25195, No. HHSN268201500001I and No. 75N92019D00031), and its contract with Affymetrix, Inc. for genotyping services (Contract No. N02-HL-6-4278). A portion of this research utilized the Linux Cluster for Genetic Analysis (LinGA-II) funded by the Robert Dawson Evans Endowment of the Department of Medicine at Boston University School of Medicine and Boston Medical Center. This study was also supported by grants from the National Institute of Aging (R01s AG033040, AG033193, AG054076, AG049607, AG059421, U01 AG058589, AG061872 and U01-AG049505) and the National Institute of Neurological Disorders and Stroke (R01-NS017950, UH2 NS100605). Dr. DeCarli is supported by the Alzheimer's Disease Center (P30 AG 010129). We thank the study participants, as well as the study team (especially the investigators and staff of the neurology team) for their contributions and dedication to the study. The authors are pleased to acknowledge that the computational work reported on in this paper was performed on the Shared Computing Cluster that is administered by Boston University Research Computing Services. URL: www.bu.edu/tech/support/research/.
GENOA: Support for the Genetic Epidemiology Network of Arteriopathy (GENOA) was provided by the National Heart, Lung and Blood Institute (HL054464, HL054457, HL054481, HL087660, and HL119443) and the National Institute of Neurological Disorders and Stroke (NS041558) of the National Institutes of Health. Genotyping was performed at the Mayo Clinic and was made possible by the University of Texas Health Sciences Center (Eric Boerwinkle, Megan L. Grove-Gaona). We would also like to thank the families that participated in the GENOA study.
GeneSTAR: The Genetic Study of Atherosclerosis Risk (GeneSTAR) was supported by grants from the National Institutes of Health National Institute of Neurological Disorders and Stroke (R01NS062059), the National Institutes of Health/National Heart, Lung, and Blood Institute (U01 HL72518, HL097698), the National Institutes of Health/National Center for Research Resources (M01-RR000052) to the Johns Hopkins General Clinical Research Center, and the National Center for Research Resources and the National Center for Advancing Translational Sciences, National Institutes of Health (UL1 RR 025005) to the Johns Hopkins Institute for Clinical & Translational Research. We would like to thank the participants and families of GeneSTAR and our dedicated staff for all their sacrifices.
i-Share: the Internet based Students HeAlth Research Enterprise (i-Share) study is conducted by the Universities of Bordeaux and Versailles Saint-Quentin-en-Yvelines (France). The i-Share study has received funding by the French National Agency (Agence Nationale de la Recherche, ANR), via the 'Investissements d'Avenir' programme (grand number ANR-10-COHO-05) and from the University of Bordeaux Initiative of Exellence

(IdEX). This project has also received funding from the European Research Council (ERC) under the European Union's Horizon 2020 research and innovation programme under grant agreement No 640643.
LLS: The Leiden Longevity Study has received funding from the European Union's Seventh Framework Programme (FP7/2007-2011) under grant agreement n° 259679. This study was supported by a grant from the Innovation-Oriented Research Program on Genomics (SenterNovem IGE05007), the Centre for Medical Systems Biology, and the Netherlands Consortium for Healthy Ageing (grant 050-060-810), all in the framework of the Netherlands Genomics Initiative, Netherlands Organization for Scientific Research (NWO), UnileverColworth and by BBMRI-NL, a Research Infrastructure financed by the Dutch government (NWO 184.021.007).
LBC1936: This project is funded by the Age UK's Disconnected Mind programme (http://www.disconnectedmind.ed.ac.uk) and also by Research Into Ageing. The whole genome association part of the study was funded by the Biotechnology and Biological Sciences Research Council (BBSRC; Ref. BB/F019394/1). Analysis of the brain images was funded by the Medical Research Council Grants G0701120, G1001245, MR/M013111/1 an MR/R024065/1. The imaging was performed at the Brain Research Imaging Centre, The University of Edinburgh (http://www.bric.ed.ac.uk), a centre in the SINAPSE Collaboration (http://www.sinapse.ac.uk). The work was undertaken by The University of Edinburgh Centre for Cognitive Ageing and Cognitive Epidemiology (http://www.ccace.ed.ac.uk), part of the cross council Lifelong Health and Wellbeing Initiative (Ref. MR/K026992/1). Funding from the BBSRC, Engineering and Physical Sciences Research Council (EPSRC), Economic and Social Research Council (ESRC), Medical Research Council (MRC) and Scottish Funding Council through the SINAPSE Collaboration is gratefully acknowledged. The LBC1936 study authors thank the nurses and staff at the Wellcome Trust Clinical Research Facility (http://www.wtcrf.ed.ac.uk), where subjects were tested and the genotyping was performed.
Sydney MAS : We acknowledge and thank the participants for their participation in the study, their supporters and the Sydney MAS Research Team (current and former staff and students). DNA was extracted by Genetic Repositories Australia, an Enabling Facility, supported by National Health & Medical Research Council Grant (NHMRC) 401184. Genome-wide genotyping was performed by the Ramaciotti Centre, University of New South Wales. Sydney MAS is supported by Australian National Health & Medical Research Council (NHMRC) Program Grants 350833, 568969 and 109308.
OATS : We gratefully acknowledge and thank the OATS participants, their supporters and the OATS Research Team (current and former staff and students). This research was facilitated through Twins Research Australia, a national resource in part supported by a Centre for Research Excellence from the NHMRC. DNA was extracted by Genetic Repositories Australia, an Enabling Facility, supported by National Health & Medical Research Council Grant (NHMRC) 401184. Genome-wide genotyping was performed by Diamantina Institute, University of Queensland. Funding for this study was awarded by a NHMRC/Australian Research Council Strategic Award (401162) and the NHMRC Project grant 1405325.
PROSPER: The Prospective Study on Pravastatin in the Elderly at Risk (PROSPER) was supported by an investigator initiated grant obtained from Bristol-Myers Squibb. Prof. Dr. J. W. Jukema is an Established Clinical Investigator of the Netherlands Heart Foundation (grant 2001 D 032). Support for genotyping was provided by the seventh framework program of the European commission (grant 223004) and by the Netherlands Genomics Initiative (Netherlands Consortium for Healthy Aging grant 050-060-810).
Rotterdam Study: The generation and management of GWAS genotype data for the Rotterdam Study (RS I, RS II, RS III) was executed by the Human Genotyping Facility of the Genetic Laboratory of the Department of Internal Medicine, Erasmus MC, Rotterdam, The Netherlands. The GWAS datasets are supported by the Netherlands Organisation of Scientific Research NWO Investments (nr. 175.010.2005.011, 911-03-012), the Genetic Laboratory of the Department of Internal Medicine, Erasmus MC, the Research Institute for Diseases in the Elderly (014-93-015; RIDE2), the Netherlands Genomics Initiative (NGI)/Netherlands Organisation for Scientific Research (NWO) Netherlands Consortium for Healthy Aging (NCHA), project nr. 050-060-810. We thank Pascal Arp, Mila Jhamai, Marijn Verkerk, Lizbeth Herrera and Marjolein Peters, and Carolina Medina-Gomez, for their help in creating the GWAS database, and Karol Estrada, Yurii Aulchenko, and Carolina Medina-Gomez, for the creation and analysis of imputed data. The Rotterdam Study is funded by Erasmus Medical Center and Erasmus University, Rotterdam, Netherlands Organization for the Health Research and Development (ZonMw), the Research Institute for Diseases in the Elderly (RIDE), the Ministry of Education, Culture and Science, the Ministry for Health, Welfare and Sports, the European Commission (DG XII), and the Municipality of Rotterdam.
This work has been performed as part of the CoSTREAM project (www.costream.eu) and ORACLE project, and has received funding from the European Union's Horizon 2020 research and innovation programme under grant agreement No 667375 and No 678543. HHHA was supported by ZonMW grant number 916.19.151.

The authors are grateful to the study participants, the staff from the Rotterdam Study and the participating general practitioners and pharmacists.
SHIP/SHIP-Trend: SHIP is part of the Community Medicine Research net of the University of Greifswald, Germany, which is funded by the Federal Ministry of Education and Research (grants no. 01ZZ9603, 01ZZ0103, and 01ZZ0403), the Ministry of Cultural Affairs as well as the Social Ministry of the Federal State of Mecklenburg-West Pomerania, and the network 'Greifswald Approach to Individualized Medicine (GANI_MED)' funded by the Federal Ministry of Education and Research (grant 03IS2061A). Whole-body MR imaging was supported by a joint grant from Siemens Healthineers, Erlangen, Germany and the Federal State of Mecklenburg West Pomerania. The University of Greifswald is a member of the Caché Campus program of the InterSystems GmbH.
TASCOG : The Tasmanian Study of Gait and Cognition is supported by Project Grants from the National Health and Medical Research Council (NHMRC IDs 403000, 491109) and a Grant from the Wicking Dementia Education and Research Centre, Hobart. Velandai Srikanth is supported by a National Health and Medical Research Council Practitioner Fellowship.
3C: The Three City (3C) Study is conducted under a partnership agreement among the Institut National de la Santé et de la Recherche Médicale (INSERM), the University of Bordeaux, and Sanofi-Aventis. The Fondation pour la Recherche Médicale funded the preparation and initiation of the study. The 3C Study is also supported by the Caisse Nationale Maladie des Travailleurs Salariés, Direction Générale de la Santé, Mutuelle Générale de l'Education Nationale (MGEN), Institut de la Longévité, Conseils Régionaux of Aquitaine and Bourgogne, Fondation de France, and Ministry of Research–INSERM Programme "Cohortes et collections de données biologiques." Christophe Tzourio and Stéphanie Debette have received investigator-initiated research funding from the French National Research Agency (ANR) and from the Fondation Leducq. We thank Dr. Anne Boland (CNG) for her technical help in preparing the DNA samples for analyses. This work was supported by the National Foundation for Alzheimer's disease and related disorders, the Institut Pasteur de Lille, the labex DISTALZ and the Centre National de Génotypage.
UKBB-I: This study was supported in part by a Grants-in-Aid from the Japan Society for the Promotion of Science (18K15410, H. Suzuki). The UK Biobank and its Imaging Enhancement are funded by the Medical Research Council and the Wellcome Trust. Data was accessed through agreement 18545 (PI, PMM). PMM acknoweldges generous support from the Edmond J Safra Foundation and Lily Safra, the National Institute for Health Research (NIHR) Biomedical Research Centre (BRC), the NIHR Senior Investigators Programme and the UK Dementia Research Institute.
UKBB-II: This study has been conducted using the UK Biobank Resource under Application Number 23509.

Supplementary Methods

1. Study description

AGES-Reykjavik Study (AGES)

The AGES-Reykjavik Study is a single center prospective cohort study based on the Reykjavik Study. The Reykjavik Study was initiated in 1967 by the Icelandic Heart Association to study cardiovascular disease and risk factors. The cohort included men and women born between 1907 and 1935 who lived in Reykjavik at the 1967 baseline examination. Reexamination of surviving members of the cohort was initiated in 2002 as part of the AGESReykjavik Study. The AGES-Reykjavik Study is designed to investigate aging using a multifaceted comprehensive approach that includes detailed measures of brain function and structure. All cohort members were European Caucasians. The study design has been described previously.[1] Briefly, as part of a comprehensive examination, all participants answered a questionnaire, underwent a clinical examination and had blood drawn. All consenting participants without contraindications were offered a brain MRI on a dedicated machine in the study center: a total of 5003 participants had an MRI.[2] Of these, 3664 were genotyped at the Laboratory of Neurogenetics, Intramural Research Program, NIA, Bethesda, Maryland, and 3219 participants passed QC criteria for genotyping. Of these, 2765 had complete genotyping and MRI data with assessment of white matter lesion burden was available. A total of 298 participants with prevalent dementia or stroke were excluded, leaving 2467 for these analyses.

Atherosclerosis Risk In Communities Study (ARIC)

The ARIC study is a population-based cohort study of atherosclerosis and clinical atherosclerotic diseases.[3] At its inception (1987-1989), 15,792 men and women, including 11,478 white and 4,266 black participants were recruited from four U.S. communities: Suburban Minneapolis, Minnesota; Washington County, Maryland; Forsyth County, North Carolina; and Jackson, Mississippi. In the first 3 communities, the sample reflects the demographic composition of the community. In Jackson, only black residents were enrolled. Participants were between age 45 and 64 years at their baseline examination in 1987-1989 when blood was drawn for DNA extraction and participants consented to genetic testing. Vascular risk factors and outcomes, including transient ischemic attack, stroke and dementia, were determined in a standard fashion. During the first 2 years (1993-1994) of the third ARIC examination (V3), participants aged 55 and older from the Forsyth County and Jackson sites were invited to undergo cranial MRI. This subgroup of individuals with MRI scanning represents a random sample of the full cohort because examination dates were allocated at baseline through randomly selected induction cycles. After excluding individuals with prevalent stroke at V3, a total of 808 white and 798 black participants had phenotypic and genome-wide genotypic data.

Austrian Stroke Prevention Study (ASPS)

The ASPS study is a single center prospective follow-up study on the effects of vascular risk factors on brain structure and function in the normal elderly population of the city of Graz, Austria. The procedure of recruitment and diagnostic work-up of study participants has been described previously.[4,5] A total of 2007 participants were randomly selected from the official community register stratified by gender and 5 year age groups. Individuals were excluded from the study if they had a history of neuropsychiatric disease, including previous stroke, transient ischemic attacks, and dementia, or an abnormal neurologic examination determined on the basis of a structured clinical interview and a physical and neurologic examination. During 2 study periods between September 1991 and March 1994 and between January 1999 and December 2003 an extended diagnostic work-up including MRI and neuropsychological testing was done in 1076 individuals aged 45 to 85 years randomly selected from the entire cohort: 509 from the first period and 567 from the second. In 1992, blood was drawn from all study participants for DNA extraction. They were all European Caucasians. Genotyping was performed in 996 participants, and the 730 who also underwent MRI scanning with assessment of white matter hyperintensity burden were available for these analyses. Genotyping was done at the Human Genotyping Facility, Genetic Laboratory Department of Internal Medicine, Erasmus MC, Rotterdam, The Netherlands.

Austrian Stroke Prevention Family Study (ASPS-Fam)

ASPS-Fam is a prospective single-center community-based study on the cerebral effects of vascular risk factors in the normal aged population of the city of Graz, Austria.[6,7] ASPS-Fam represents an extension of the Austrian Stroke Prevention Study (ASPS), which was established in 1991.[4,5] Between 2006 and 2013, study participants of the ASPS and their first-grade relatives were invited to enter ASPS-Fam. Inclusion criteria were no history of previous stroke or dementia and a normal neurologic examination. A total of 419 individuals from 176 families were included into the study. The number of members per family ranged from 2 to 6. The entire cohort

underwent a thorough diagnostic workup including clinical history, laboratory evaluation, cognitive testing, and an extended vascular risk factor assessment. They were all European Caucasians. Those 274 participants who passed genotyping quality control and underwent MRI scanning were available for these analyses.

Cardiovascular Health Study (CHS)

The CHS is a population-based cohort study of risk factors for vascular disease in adults 65 years or older conducted across 4 field centers in the United States: Sacramento County, California; Washington County, Maryland; Forsyth County, North Carolina; and Pittsburgh, Allegheny County, Pennsylvania.[8] The original predominantly white cohort of 5,201 persons was recruited in 1989-1990 from a random sample of people on Medicare eligibility lists. An additional 687 African-Americans were enrolled in 1992-1993, for a total sample of 5,888.

Vascular risk factors and outcomes, including transient ischemic attack, stroke and dementia, were determined in a standard fashion.[9,10]

Chicago Health and Aging Project (CHAP)

The Chicago Health and Aging Project (CHAP) is a longitudinal, population-based study of Alzheimer's disease and other common health conditions among adults age 65 years and older conducted from 1993-2012 described in great detail previously.[11] Beginning in 1993, 78.7% of all residents over 65 years old (defined by a door-to-door census) of a geographically defined, biracial (63% African Americans) Chicago community were enrolled in CHAP. From 2001, community residents who reached age 65 were also enrolled as successive cohorts. Of the total 10,802 participants enrolled in CHAP, 6,158 were enrolled as members of the original cohort and 4,644 as members of the successive age cohorts. Data were collected triennially for six cycles. At the end of the Cycle 2 interview, a detailed clinical evaluation in a stratified random sample of the population about one-sixth of all participants who had a population interview. A total of 2,864 subjects were selected for the detailed clinical evaluations during which time DNA samples were collected and analyzed at the Broad Institute. Of those subjects, 952 subjects had MRI scans and were eligible to be part of this analysis.

Coronary Artery Risk Development in Young Adults (CARDIA) Study

The CARDIA study is a population based, prospective cohort examining the development and determinants of clinical and subclinical cardiovascular disease and its risk factors.[12] The CARDIA study initial enrollment consisted of 5,115 European Americans and African American men and women between 18 and 30 years old (52% African American and 55% women). The study is multicenter with recruitment in Birmingham, AL; Chicago, IL; Minneapolis, MN; and Oakland, CA. The IRB at each of the study sites approved the study protocols, and written informed consent was obtained from all participants. Baseline measurements were repeated, and additional measurements performed, at Years 2, 5, 7, 10, 15, 20, and 25. All participants gave informed consent and the study was approved by all relevant institutional review boards for human use.

Framingham Heart Study (FHS)

The FHS is a three-generation, single-site, community-based, prospective cohort study that was initiated in 1948 to investigate risk factors for cardiovascular disease including stroke. It now comprises 3 generations of participants: the original cohort followed since 1948 (Original);[13] their offspring and spouses of the offspring, followed since 1971 (Offspring);[14] and children from the largest offspring families enrolled in 2002 (Gen 3).[15] The Original cohort enrolled 5209 men and women who comprised two-thirds of the adult population then residing in Framingham, MA, USA. Survivors continue to receive biennial examinations. The Offspring cohort comprises 5,124 persons (including 3,514 biological offspring) who have been examined approximately once every 4 years. Participants in the first two generations were invited to undergo an initial brain MRI in 1999-2005. Brain MRI in Gen 3 only began in 2009 and is not included in these analyses. The population of Framingham was virtually entirely whites in 1948 when the Original cohort was recruited. Vascular risk factors and outcomes, including transient ischemic attack, stroke and dementia, were identified prospectively since 1948 through an ongoing system of FHS clinic and local hospital surveillance.[16,17] Of the 4,519 persons underwent genotyping and passed QC, 4,116 were alive in 1999 when the MRI study began. Of these, 2,319 participants from the Original and Offspring cohorts have undergone cranial MRI with measurement of white matter hyperintensity burden. Of these, 87 participants were excluded for stroke or TIA, 6 for dementia and 26 because of other neurological conditions such as brain tumors or severe head injury that might confound the assessment of white matter hyperintensity volume. The remaining 2,200 participants constitute the FHS sample for this study.

Genetic Epidemiology Network of Arteriopathy (GENOA)

The Genetic Epidemiology Network of Arteriopathy (GENOA) study, a part of the

Family Blood Pressure Program,[18] consists of hypertensive sibships that were recruited for linkage and association studies in order to identify genes that influence blood pressure and its target organ damage.[19] In the initial phase of the GENOA study (Phase I: 1996-2001), all members of sibships containing ≥ 2 individuals with essential hypertension clinically diagnosed before age 60 were invited to participate, including both hypertensive and normotensive siblings.

In the second phase of the GENOA study (Phase II: 2000-2004), 1241 European American and 1482 African American participants were successfully re-recruited to measure potential target organ damage due to hypertension. As part of an ancillary study (2001-2006), Phase II GENOA participants that had a sibling willing and eligible to participate underwent a brain MRI (N=916 European Americans and 830 African Americans). Genotyping was performed by the Center for Individualized Medicine's Medical Genome Facility at the Mayo Clinic. Participants were excluded from this analysis if they had unusable MRI data (due to cortical infarctions, masses metallic artifacts, or failure to complete MRI), had history of stroke or dementia, or had unavailable genotype data. After exclusions, a total of 789 European American and 599 African American participants were available for analysis.

Genetic Studies of Atherosclerosis Risk (GeneSTAR)

GeneSTAR is an ongoing prospective study designed to determine environmental, phenotypic, and genetic causes of premature cardiovascular disease.[20] Participants (n = 3,533) were recruited from European- and African-American families (n = 891) identified from 1983-2006 from probands with a premature coronary disease event prior to 60 years of age who were identified at the time of hospitalization in any of 10 Baltimore area hospitals. Apparently healthy siblings of the probands and offspring of the siblings and probands were screened for traditional coronary disease and stroke risk factors. A random subset of this study population participated in an MRI study between 2009 and 2013.[21] Siblings and offspring were excluded if they had a history of chronic corticosteroid use, life-threatening diseases, neurologic diseases that would preclude accurate MRI interpretation, and implanted metals that prohibited MRI scans. Participants with atrial fibrillation or symptomatic cardiovascular disease of any kind were excluded from the study.

i-Share Study

This study was used for the secondary analysis of association between a weighted genetic risk score of WMH burden and MRI-markers of white matter integrity on diffusion tensor imaging in young health adults. The Internet-based Students HeAlth Research Enterprise (i-Share) study is a prospective population-based cohort of students in higher education institutions in France. The i-Share is the largest ongoing epidemiological study conducted on students' health. The aims of the i-share cohort are (i) to assess the health's state and well-being of students, (ii) to study risk behaviors in this specific population, (iii) the frequency and consequences of various diseases in young adults, as well as, the pathophysiological mechanisms of certain diseases, such as diseases affecting older persons which have a long preclinical phase and for which intermediate biomarkers could already be measured at a very young age. Students enrolled at a university or other higher education institution, who were at least 18 years old, and who are able to understand written French were eligible to participate on a voluntary basis via the i-share website (http://www.i-share.fr/). Currently, over 20,000 participants have been included, of whom 1 999 participants from the Bordeaux University site, who provide written informed consent, were enrolled in an ancillary study involving brain MRI (acquired on a Siemens 3T Prisma scanner) and genetic testing, including genome-wide genotyping on the Affymetrix Precision Medicine Axiom array (imputed on the HRC reference panel, Supplementary Data 1). Of these, 1,738 participants had both high quality brain MRI and European ancestry specific genome-wide genotype data available. Diffusion-Weighted Imaging (DWI) was conducted using 2D-EPI with 1.7 mm cubic voxels and 100 directions multi-shell/multiband sequences, and images were analyzed with a custom pipeline applying standard preprocessing and computations using FSL and dipy tools.

Leiden Longevity Study (LLS)

The Leiden Longevity Study (LLS) (http://www.molepi.nl/research/longevity) consists of 421 nonagenarian sibling pairs aged older than 89 years for men and 91 years for women, their 1,671 offspring and the 744 partners thereof.[22] The middle aged study population of the LLS, excluding the nonagenarian siblings, consisted of 2,415 participants. The Medical Ethical Committee of the Leiden University Medical Centre approved the study and informed consent was obtained from all participants. MRI scan was taken from 367 unrelated participants and blood pressure has been determined at the same day.

Lothian Birth Cohort 1936 (LBC1936)

The LBC1936 consists of relatively healthy individuals assessed on cognitive and medical measures at age 70 years (n=1,091), and again with brain imaging traits at 73 years of age (n=866). They were born in 1936, most took part in the Scottish Mental Survey of 1947, and almost all lived independently in the Lothian region of

Scotland. A full description of participant recruitment and testing can be found elsewhere.[23,24] The study was approved by the Lothian (REC 07/MRE00/58) and Scottish Multicentre (MREC/01/0/56) Research Ethics Committees and all subjects give written informed consent. There are 621 individuals with GWAS and white matter lesion data. The following individuals were excluded (MMSE < 24 n=5, unknown MMSE n=1, stroke n=42) giving a final sample of 573 (303 Males, 270 Females).

Sydney Memory and Ageing Study (MAS)

The Sydney Memory and Ageing Study began in 2005 and is a longitudinal community-based study investigating mild cognitive impairment and the rate of cognitive change over time. Participants aged 70-90 years were randomly recruited from the compulsory electoral roll in Sydney, Australia. Exclusion criteria included limited English or a medical/psychological condition that would prevent them from completing assessments, dementia diagnosis, an age and education-adjusted MMSE score <24, psychotic symptoms, or a diagnosis of schizophrenia/bipolar disorder and/or a progressive malignancy. All participants provided informed written consent and the ethics committees of the University of New South Wales and the the South Eastern Sydney and Illawarra Area Health Service approved the study. At baseline, there were 1037 participants with a mean age of 78.84 years and 44.8% were men. Further details are provided in Sachdev et al., (2010).[25] For the current study, there were 522 individuals with genome-wide genotyping and WMH data available for analysis.

Older Australian Twins Study (OATS)

Participants were recruited from the Australian Twin Registry and also through a recruitment drive. At baseline, participants were aged 65 years and over. Inclusion criteria included an ability to consent, a co-twin who also consented to participate, completion of some education in English and residence in one of the three eastern states (Victoria, New South Wales, Queensland). Exclusion criteria included inadequate English to complete the assessment, current diagnosis of malignancy or other life-threatening medical illness and/or a current acute psychosis diagnosis. Informed consent was obtained from all participants and the ethics committees of the Australian Twin Registry, University of New South Wales, University of Melbourne, Queensland Institute of Medical Research and the South Eastern Sydney and Illawarra Area Health Service. At baseline, there were 623 participants with a mean age of 70.77 years and 65.2% of the sample were women. For further details see Sachdev et al. (2009)[26] and Sachdev et al. (2011).[27] For the current study, there were 370 individuals with genome-wide genotyping and WMH data available for analysis.

The Prospective Study on Pravastatin in the Elderly at Risk (PROSPER)

PROSPER was a prospective multicenter randomized placebo-controlled trial to assess whether treatment with pravastatin diminishes the risk of major vascular events in elderly. Between December 1997 and May 1999, we screened and enrolled subjects in Scotland (Glasgow), Ireland (Cork), and the Netherlands (Leiden). Men and women aged 70-82 years were recruited if they had pre-existing vascular disease or increased risk of such disease because of smoking, hypertension, or diabetes. A total number of 5804 subjects were randomly assigned to pravastatin or placebo. A large number of prospective tests were performed including Biobank tests and cognitive function measurements. A detailed description of the study has been published elsewhere.[28,29]

Rotterdam Study (RSI, RSII, RSIII)

The Rotterdam Study is a population-based cohort study among inhabitants of a district
of Rotterdam (Ommoord), The Netherlands, and aims to examine the determinants of disease and health in the elderly with a focus on neurogeriatric, cardiovascular, bone, and eye disease.[30] In 1990-1993, 7,983 persons aged 55 years and older participated and were re-examined every 3 to 4 years (Rotterdam Study I). In 2000-2001 the cohort was expanded by 3,011 persons aged 55 and over who had not yet been part of the Rotterdam Study (Rotterdam Study II). In 2006-2008 a second expansion (Rotterdam Study III) of 3,932 persons aged 45 and over was realized. All participants had DNA extracted at their first visit. Genotyping was attempted in participants with high-quality extracted DNA in 2007-2008. In total, 6,291 samples from the Rotterdam Study I, 2,157 samples from Rotterdam Study II and 3,048 samples from Rotterdam Study III were available with good quality genotyping data. Genotyping was done at the Human Genotyping Facility, Genetic Laboratory Department of Internal Medicine, Erasmus MC, Rotterdam, the Netherlands.
In 1995-1996, 563 non-demented persons of the 7,983 participants from the Rotterdam
Study I were randomly selected in strata of age and sex to undergo cranial MRI scanning. From 2005 onwards, cranial MRI scanning including assessment of cerebral white matter lesion burden was added to the core protocol.[31] As a result, 1,081, 1,138, and 2,564 participants from respectively Rotterdam Study I, II and III had been scanned and genotyped and were available for the discovery analysis.

Study of Health in Pomerania (SHIP, SHIP-TREND)

We analyzed data from the Study of Health in Pomerania (SHIP).[32] The target population was comprised of adult German residents in northeastern Germany living in three cities and 29 communities, with a total population of 212,157. A two-stage stratified cluster sample of adults aged 20-79 years (baseline) was randomly drawn from local registries. The net sample (without migrated or deceased persons) comprised 6,267 eligible subjects, of which 4,308 Caucasian subjects participated at baseline SHIP-0 between 1997 and 2001. Follow-up examination (SHIP-1) was conducted 5 years after baseline and included 3300 subjects. From 2008 to 2012 the third phase of data collection (SHIP-2, N=2333) was carried out. Concurrent with SHIP-2 a new sample called SHIP-Trend-0 (N=4420) in the same area was drawn in 2008 and similar examinations were undertaken. SHIP and SHIP TREND were approved by the local ethics committee. After complete description of the study to the subjects, written informed consent was obtained.
Subjects from SHIP-2 and SHIP-TREND-0 were asked to participate in a whole-body magnetic resonance imaging (MRI) assessment.[33] After exclusion of subjects who refused participation or who fulfilled exclusion criteria for MRI (e.g. cardiac pacemaker) 1183 subjects from SHIP-2 and 2189 subjects from SHIP-Trend-0 underwent the MRI scanning (total number n=3372). After exclusion of scans with technical artifacts, major structural abnormalities and stroke, full data sets with GWAS data and MRI scans were available in 981 subjects in SHIP-2 and 824 subjects in TREND and included in this project.

Tasmanian Study of Cognition and Gait (TASCOG)

TASCOG is a study of cerebrovascular mechanisms underlying gait, balance and cognition in a population-based sample of Tasmanian people aged at least 60 years. Individuals aged 60–86 years (N=395) living in Southern Tasmania, Australia, were randomly selected from the electoral roll to participate in the study. Individuals were excluded if they lived in a nursing home, had a contraindication for magnetic resonance scanning (MRI) or were unable to walk without a gait aid.[34] DNA was extracted from peripheral blood samples by proteinase K digestion following cell lysis, then phenol-chloroform purification. DNA was genotyped at the Diamantina Institute and Institute of Molecular Biosciences, University of Queensland, Australia, for 370 participants. Genotypes for 22 individuals were excluded, either because they were closely related to other individuals, they were outliers in a population ancestry analysis or their sex predicted from genotypes did not match sex as recorded in the database. Among the 348 remaining participants with available genome-wide data, 343 had MRI data and, after exclusion of 28 participants for stroke, 315 individuals were available for the present study.

Three-City Dijon Study (3C-Dijon)

The 3C is a cohort study conducted in three French cities (Bordeaux, Dijon, and Montpellier), comprising 9,294 participants, designed to estimate the risk of dementia and cognitive impairment attributable to vascular factors.[35] Eligibility criteria included living in the city and being registered on the electoral rolls in 1999, 65 years or older, and not institutionalized. The study protocol was approved by the Ethical Committee of the University Hospital of Kremlin-Bicêtre and each participant signed an informed consent.
Data reported in this article were obtained in Dijon (3C-Dijon study), where 4,931 individuals were recruited (1999 –2001). The overall design of the 3C-Dijon study is detailed elsewhere.[35-37] Participants aged less than 80 years and enrolled between June 1999 and September 2000 (n=2,763) were invited to undergo a brain MRI. Although 2,285 subjects agreed to participate (82.7%), because of financial limitations, 1,924 MRI scans were performed, of which 120 were not interpretable. Thus, cerebral white matter lesion measures were available in 1800 participants. Of these, 8 individuals were excluded because of prevalent dementia, 79 because of stroke, and 6 because of brain tumor, leaving 1,707 participants. MRIs were acquired from a 1.5-Tesla Magnetom scanner (Siemens, Erlangen, Germany). T1- and T2-weighted images of each subject were first aligned to each other using the AIR package. These images were then further analyzed with the optimized Voxel-Based Morphometry (VBM) protocol, using Statistical Parametric Mapping 99 (SPM99) that we modified in order to take into account the structural characteristics of the aged brain, as described in detail elsewhere. Fully automated image processing software was developed to detect, measure, and localize white matter hyperintensities (WMH).[38] Of the remaining 1,707 individuals with brain MRI data, 1,578 had QC'ed genetic data and after exclusion of participants with prevalent stroke, prevalent dementia or brain tumor 1,491 individuals were available for the genetic analysis.

UK BioBank (UKBB)

UK Biobank is a prospective study that recruited 502 620 community-dwelling participants from across the United Kingdom between 2006 and 2010, aged 40 to 69 years (http://www.ukbiobank.ac.uk). The study collects extensive data from questionnaires, interviews, health records, physical measures, biological samples, and

imaging. A subset of the participants also underwent brain MRI. Patients with a baseline diagnosis of stroke, multiple sclerosis, Parkinson disease, any other neurodegenerative problem (*InternationalClassification of Diseases*, *Ninth Revision/Tenth Revision*, or self-report or health-record linkage) or no genetic data were excluded. UK Biobank received ethical approval from the research ethics committee (reference 11/NW/0382). All participants provided informed consent to participate. Procedures for brain imaging acquisition and initial quality check have been described previously and are available on the UK Biobank website (Brain Imaging Documentation V1.3; http://www.ukbiobank.ac.uk). In brief, all brain MRI data were acquired on a single standard Siemens Skyra 3T scanner (Siemens Medical Solutions, Germany) using the standard Siemens 32-channel radiofrequency receiver head coil. Within this study the initial releases of imaging data were used for UKBB-I, whereas releases 3 and 4 have been used for UKBB-II. UKBB-I sample consisted of 19,291 unrelated individuals with both MRI and genetic data, after excluding 69 individuals due to the presence of neurological disorders at the baseline 19,222 indivudals were available for the genetic analysis. UKBB-II sample consisted of 7,709 unrelated individuals with useable imaging and genetic data, 143 individuals were excluded due to stroke and other neurological disorders at baseline, leaving 7,566 for analysis within this study.

2. Online resources (URLs) and extended methods

Gene expression weights for TWAS: http://gusevlab.org/projects/fusion/;
HESS/ρ-HESS: https://huwenboshi.github.io/hess/local_hsqg/;
LDSR: https://github.com/bulik/ldsc;
GWAS-PW: https://github.com/joepickrell/gwas-pw;
Radial-MR: https://github.com/WSpiller/RadialMR;
GREP: https://github.com/saorisakaue/GREP;
EPIGWAS: https://immunogenomics.hms.harvard.edu/code;
Histone regulatory marks: http://egg2.wustl.edu/roadmap/data/byFileType/peaks/consolidated/narrowPeak/;
Magma.Celltyping: https://github.com/NathanSkene/MAGMA_Celltyping;
MR-MEGA: https://www.geenivaramu.ee/en/tools/mr-mega;
Matrisome: http://matrisomeproject.mit.edu/

SBP and DBP risk score construction in UK Biobank:

Genome-wide significant SNPs identified for SBP (n=473) and DBP (n=477) from the recent and largest blood-pressure (BP) GWAS[58], were used as the instruments in constructing the risk score. Only pairwise independent, LD-filtered ($r2 < 0.1$, 1000 Genomes European panel) were considered. Effect estimates from the International Consortium of Blood Pressure-Genome Wide Association Studies (ICBP) were used for previously reported variants and from the replication meta-analysis for all the variants newly reported by Evangelou E et al. Effect estimates for SBP and DBP were then weighted with BP increasing alleles in the UK biobank particpants with WMH measures (n=19,222) and summed. Association statistics for GW significant WMH SNPs (n=25) and WMH wGRS (in aggregate) with WMH values were estimated in four equal sized bins stratified based on the SBP and DBP risk score distribution.

Mixed-linear model association:

Mixed linear model (MLM) was used to test the association of SNPs with each individual DTI traits, accounting for possible relatedness structure in the sample by calculating genetic relationship matrix (GRM) as implemented in the "mlma-loco" scheme in GCTA[67].

$$y = a + bx + g^{-} + e$$

Where y is the investigated trait-outcome, a is the mean term, b is the additive effect of the SNP tested for association, x is the SNP genotype dose, g- is the accumulated effect of all SNPs except those on the chromosome where the tested SNP is located.

Matrisome annotation :

The web-based annotation tool (http://matrisomeproject.mit.edu/; The matrisome project[68]) was used to annotate the genome-wide associated WMH risk loci by querying the HGNC symbol of the gene nearest to the index WMH SNP. Based on the in silico and in vivo approach, the matrisome project curates the protein composition of the extracellular matrix (ECM) and classifies it in to the core protein component that constitutes the structure of ECM, and the interactive component that interacts with the core unit (ECM-affiliated proteins, ECM-regulators and secreted factors).

Supplementary table 1: MRI protocol and phenotyping

Study	Scanner	MRI protocol	WMH detection and quantification*
AGES	1.5 T Signa Twinspeed EXCITE system (General Electric Medical Systems, Waukesha, W)	The AGESRS/MNI pipeline, that segments the whole brain (cerebrum and cerebellum) into GM, normal WM (referred to as NWM), WMH and CSF. The pipeline is multispectral i.e. it uses the contrast properties from all the different pulse sequences in the tissue segmentation process. The scanning protocol includes a proton density (PD)/T2 -weighted fast spin echo (FSE) sequence (TE1, 22 ms; TE2, 90 ms; TR, 3220 ms; echo train length, 8; FA, 90°; FOV, 220 mm; matrix, 256 × 256), and a fluid attenuated inversion recovery (FLAIR) sequence (TE, 100 ms; TR, 8000 ms, inversion time, 2000 ms, FA, 90°; FOV, 220 mm; matrix, 256 × 256). These latter two sequences were acquired with 3-mm thick slices and in-plane pixel size of 0.86 mm x 0.86 mm. All images were acquired to give full brain coverage and were localized at the AC/PC commissure line.	Defects in the brain parenchyma are identified with a signal intensity isointense to that of CSF on all MR images. They are classified as CSF and areas with increased signal on PD, T2 and FLAIR images associated with parenchymal defects as WMH.<br>Total white matter lesion (WML) volume was computed automatically with an algorithm based on the Montreal Neurological Institute pipeline.[39] The AGES-Reykjavik/Montreal Neurological Institute pipeline has been modified to accommodate full brain coverage including cerebellum and brainstem, multispectral images (T1-weighted three-dimensional spoiled gradient echo sequence, FLAIR, and proton density/T2-weighted fast spin echo sequences), high throughput, and minimal editing. The classification of WML was achieved with an artificial neural network classifier in the four dimensional intensity space defined by the four imaging sequences. The classifier was trained by the input of manually labeled image data from the four sequences.[40]
ARIC	General Electric (General Electric Medical Systems) or Picker (Picker Medical Systems) 1.5-Tesla.	The scanning protocol included a series of sagittal T1-weighted scans and axial proton-density, T2-weighted and T1-weighted scans with 5 mm thickness and no interslice gaps. Images were interpreted directly from a PDS-4 digital workstation consisting of four 1024 X 1024-pixel monitors capable of displaying all 96 images simultaneously. Both ARIC and CHS used the same protocols for scanning and for interpretation.[41]	WMHs were estimated as the relative total volume of periventricular and subcortical white matter signal abnormality on proton density–weighted axial images by visual comparison with eight templates that successively increased from barely detectable white matter changes (Grade 1) to extensive, confluent changes (Grade 8). Individuals with no white matter changes received Grade 0, and those with changes worse than Grade 8 received Grade 9.
CHS	General Electric or Picker 1.5-Tesla scanners at 3 field centers and on a 0.35-Tesla Toshiba scanner at the fourth	Both ARIC and CHS used the same protocols for scanning and for interpretation.[42]	Both ARIC and CHS used the same protocols for scanning and for interpretation. WMH were rated visually on a 0-9 Scale.[42]
ASPS	1.5-Tesla whole body imaging systems (Gyroscan S 15 and ACS, Philips Medical Systems, Eindhoven, The Netherlands)	We performed axial proton-density and T2-weighted sequences. Additionally, T1-weighted images were acquired in the sagittal plane. For all images, slice thickness was 5 mm with no interslice distance.[43]	Axial PDW sequences were used for WMH quantification. Lesion load measurements were done on proton density–weighted images on an UltraSPARC workstation (Sun Microsystems) using DISPImage16.[43] Using a hard copy with all lesions outlined as a reference, a trained technician outlined all lesions on the computer image with use of a semi-automated segmentation algorithm provided by the DISPImage program. The total lesion volume was calculated by multiplying the total lesion area by slice thickness.
ASPS-Fam	3 Tesla whole body scanner (TimTrio; Siemens Healthcare, Erlangen, Germany)	Fluid-attenuated inversion recovery (FLAIR) sequence	WMHs were recorded on fluid-attenuated inversion recovery images as previously described.[5]<br>WMHs were outlined using a home-written IDL program (Exelis Visual Information Solutions, USA). They were semiautomatically

			segmented by combined region growing and local thresholding after manual selection. Total WMH volume (cube millimeter) was calculated from the lesion masks using the program FSLMATHS (FSL, Oxford, http://www.fmrib.ox.ac.uk/).
CHAP	GE 1.5 Telsa Scanner (Excite platform, V11)	A single gaussian distribution is fitted to image data and a segmentation threshold for white matter hyper intensity volume was determined a priori as 3.5 SDs in pixel intensity above the mean of the fitted distribution of brain parenchyma. The following sequences were used:<br>1. Sagittal 2D spin echo locator sagittal T1, TE=9 ms (minimum), TR=500 ms, Slice thickness: 5 mm, slice spacing: 1 mm, FOV: 25 cm x 18.75 cm, matrix: 256 x 256, NEX: 1, Bandwidth: 15.63 KHz Phase FOV: 0.75, Freq Dir: S/I, Inferior Saturation On, Flow comp On. Scan Time: 1 minute 44 seconds.<br>2. Sagittal 2D multi-slice dual spin-echo axial PD/T2, TE=30, 80 ms, TR=5000 ms, Slice Thickness: 3 mm, slice spacing: 0 mm, FOV: 25 cm x 18.75 cm, matrix: 256 x 256, NEX: 1, Bandwidth: 15.63 KHz, Phase FOV: 0.75, Freq Direction: A/P, Inferior Saturation On, Flow comp On. Scan time: 17 minutes.<br>3. Axial-oblique 3D Fast Spoiled Gradient Recalled Echo (FSPGR) Sequence. TE: 2.9 ms (min), TR: 9 ms (min), Flip angle: 15 deg, Slice thickness: 1.5 mm, slice spacing: 0.0 mm, Number of Slices: 128, NEX: 2, FOV: 25 cm x 25 cm, Matrix: 256 x 256, Bandwidth: 15.63 KHz, Phase FOV: 1.00, Freq Direction: A/P, Options: Increased image dynamic range: On (CV User 2: 40.00, CV User 4: 8.00). Scan time: 7 min. 33 sec.<br>4. Axial-oblique 2D Fluid Attenuated Inversion Recovery (FLAIR) Fast Spin Echo sequence:<br>TE: 144 ms, TR: 11000 ms, TI: 2250 ms, Flip Angle: 90 deg, Slice thickness: 3 mm, slice spacing: 0.0 mm (Interleaved), FOV: 22 cm x 22 cm, NEX: 1, Matrix: 256 (freq) x 192 (phase), Bandwidth: 15.63 KHz, Phase FOV: 1.00, Freq Direction: A/P Options: Superior/Inferior saturation pulse On (80 mm thick). Scan time : 5 min 8 sec.	The segmentation algorithm was based on an Expectation-Maximization (EM) algorithm that iteratively refines its segmentation estimates to produce the most consistent outputs using the native-space T1 images along with a model of image smoothness.[44,45] The initial estimate was obtained from the template-space warps of previously segmented images, since locations of WM/GM/CSF tissues are known in the template space, transforming these masks back to the each image's native space produces rough estimate 3-tissue segmentations. Using these initial values, a Gaussian model of T1-weighted image intensity for each tissue class was used to produce a segmentation. The segmentation yielded by these appearance models was then refined using a Markov Random Field (MRF) model, a computational statistical method that efficiently produces a label map consistent with both the input intensities and image smoothness statistics. Inference in the MRF is computed using an adaptive priors model.[46] This refined segmentation from the MRF is then used to compute new Gaussian intensity models for each tissue class, and the algorithm repeats, iteratively switching between calculating Gaussian appearance models and MRF-based segmentation, until convergence. The MRF-based segmentation at the final iteration was used as the final output segmentation.<br>The multiple sets of predefined regions of interest including lobar volumes, the Desikan-Killiany Atlas from Freesurfer and Brodmann areas were defined by an expert anatomist.[47] Regional measures were calculated by back transformation of the atlas into segmented image native space at the imaging for dementia and aging (IDeA) lab. A voting scheme was used to assure precise labelling of each region after interpolation of the atlas into native space.
CARDIA	MRI scanning was conducted in conjunction with the Y25 examination at 3 of the 4 field centers : Birmingham, AL ; Minneapolis, MN, and Oakland, CA using 3T scanners (Oakland: Siemens 3T Tim Trio/VB 15 platform ; Minneapolis : Siemens 3T Tim Trio/VB 15 platform	We used the following pulse sequences for morphological analysis : Sagital 3DT2 : TR 3200 ms; TE 40 ms; FOV 250 mm; Matrix 256X256 ; slice thickness 1 mm; Sagital 3D FLAIR : TR 6000 ms ; TI 2200 ms; TE 160 ms ; FOV 250 mm ; Matrix 256X256 ; slice thickness 1 mm; and Sagital 3D MPRAGE: TR 1900 ms; TI 900 ms; TE 2.89 ms ; FA 9 deg ; FOV 250 mm ; Matrix 256X256 ; slice thickness 1mm.	Structural MR images were processed using previously described methods that were based on an automated multispectral computer algorithm that classifies all supratentorial brain tissue into GM, WM, and CSF. GM and WM were further characterized as normal and abnormal (ischemic).[48] A total of 719 participants (428 whites; 291 blacks) had usable MRI sequences.

	and Birmingham: Philips 3T Achieva/2.6.3.6 platform)		
FHS	1 or 1.5 T Siemens Magnetom scanner	3D T1 and double echo proton density (PD) and T2 double spin echo coronal images were acquired in 4-mm contiguous slices from nasion to occiput with a repetition time [TR] 2420 msec, an echo time [TE] of TE1 20/TE2 90 msec, an echo train length of 8, a field of view [FOV] of 22 cms, and an acquisition matrix of 192 X 256 interpolated to 256 X 256 with one excitation.<br>All MR images were transferred to the centralized reading center at the University of<br>California–Davis Medical Center and analyses were performed on QUANTA 6.2, a customdesigned image analysis package operating on a Sun Microsystems Ultra 5 workstation. Images were analysed and interpreted blind to subject data and in random order. Semi-automated analysis of pixel distributions, based on mathematical modeling of MRI pixel intensity histograms for cerebrospinal fluid (CSF) and brain matter (white matter and gray matter), were used to determine the optimal threshold of pixel intensity to best distinguish CSF from brain matter based on previously published methods. The intracranial vault above the tentorium was outlined manually to determine the total intra-cranial volume (TCV).	For segmentation of WMH from other brain tissues the first and second echo images from T2 sequences were summed and a lognormal distribution was fitted to the summed data (after removal of CSF and correction of image intensity non-uniformities). A segmentation threshold for WMH was determined as 3.5 standard deviations in pixel intensity above the mean of the fitted distribution of brain parenchyma. These methods have been shown to have high inter- and intra- rater reliabilities in previous studies with F values ranging from 7 to 19.
GENOA	Signa 1.5 T MRI scanner<br>(GE Medical Systems, Waukesha, WI, USA)	Interactive imaging processing steps were performed by a research associate who had no knowledge of the subjects' personal or medical histories or biological relationships. The methods for semiautomated MRI measurements of brain anatomy have been described previously.[49]	A fully automated algorithm was used to segment each slice of the edited multi-slice FLAIR sequence into voxels assigned to one of three categories: brain, cerebrospinal fluid, or leukoaraiosis. Total intracranial volume (head size) was measured from T1-weighted spin echo sagittal images, each set consisting of 32 contiguous 5 mm thick slices with no interslice gap, field of view = 24 cm, matrix = 256 x 192, obtained with the following sequence: scan time = 2.5 min, echo time = 14 ms, repetitions = 2, replication time = 500 ms.24 Total brain and leukoaraiosis volumes were determined from axial fluid-attenuated inversion recovery (FLAIR) images, each set consisting of 48 contiguous 3-mm interleaved slices with no interslice gap, field of view = 22 cm, matrix = 256 x 160, obtained with the following sequence: scan time = 9 min, echo time = 144.8 ms, inversion time = 2,600 ms, repetition time = 26,002 ms, bandwidth = +/- 15.6 kHz, one signal average.
GeneSTAR	Philips 3T imaging	MPRAGE images were skull- stripped and co-registered to FLAIR images. Spatial normalization of the co-registered MPRAGE and FLAIR images into MNI space was performed via affine transformation. Following sequences were used for WMH quantification: Axial T1-weighted MPRAGE: TR TE, TI (inversion time), 1mm contiguous, FOV 240, matrix 256x256x160;Axial turbo spin echo FLAIR (fluid attenuation inversion recovery): TR 11000ms, TI	A trained rater manually delineated the WMHs on the normalized FLAIR images (with reference to the MPRAGE images for verification of pathology) using Medical Image Processing, Analysis, and Visualization (MIPAV) software. We segmented the brain in native MPRAGE space using an automated probabilistic methodology that employs a topology-preserving algorithm and mapped the resulting tissue mask to MNI space. We measured total brain, intracranial, cortical grey matter, and white matter volumes in native MPRAGE space and WMH volumes in MNI Space. Total brain volume (in cubic millimeters) was identified as the sum of white matter, WMH, and grey matter volume from the vertex of the

			brain to the foramen magnum. Intracranial volume was defined (in cubic millimeters) as the sum of all meningeal material, soft tissue, and sulcal and ventricular cerebrospinal volumes inferior to bone from the vertex to the foramen magnum.
i-Share	3T Siemens Prisma	The MRI protocol lasted about 40 minutes and included the following sequences:<br><br>- 3D T1-weighted MPRAGE sagittal acquisition , TR/TE/TI = 2000/2.0/880 ms, repeat x2, producing 1 mm3 isotropic T1w volumes covering the whole brain<br>- 3D T2-weighted FLAIR SPACE sagittal acquisition, TR/TE/TI = 5000/394/1800 ms, repeat x2, producing 1 mm3 isotropic T2w fluid attenuated volumes covering the whole brain<br>- 2D T2*-DWI axial acquisition, echoplanar imaging, TR/TE=3540/75.0 ms, multiband x3, 100 directions, multishell b=0 s/mm2 (8+8 phase-encoding reversed), b=300 s/mm2 (8 dir), b=1000 s/mm2 (32 dir), b=2000 s/mm2 (60 dir), producing 1.75 mm3 isotropic diffusion weighted volumes covering the whole brain<br>- 3D T2*-SWI axial acquisition, TR/TE1=24.0/9.42 ms producing 0.8x0.8x3 mm3 anisotropic T2*-susceptibility weighted volumes (43 slices) covering the whole brain<br>- 2D T2*-BOLD resting state axial acquisition, echoplanar imaging, TR/TE=800/35.0 ms, multiband x6, producing 2.4 mm3 isotropic blood oxygen level-weighted volumes, covering the whole brain (66 slices)	To examine the lifetime impact of WMH risk variants, we explored the association of a weighted genetic risk score for WMH with MRI-markers of white matter integrity in i-Share participants, using DTI parameters, estimated as follows :<br><br>DWI was conducted and images were analyzed with an FSL derived custom software.[50] Briefly, white matter tracts were "skeletonized" with Tract-Based Spatial Statistics (TBSS).[51] and a diffusion histogram analysis was performed, as previously described[52] to derive DTI metrics measuring the integrity of the white matter microstructure, including fractional anisotropy (FA) and mean diffusivity (MD), as well as peak width of skeletonized mean diffusivity (PSMD). PSMD was calculated using a fully automated method via a shell script (freely available at www.psmd-marker.com). Shortly, FA data, nonlinearly aligned with FNIRT (FMRIB Nonlinear Image Registration Tool) into a common space (standard space FMRIB 1 mm FA template), were projected onto the skeleton, which was derived from standard-space template. MD images were then projected onto the same skeleton, using the FA-derived projection parameters. Histogram analysis of the MD data derived from the skeleton was performed. PSMD was calculated as the difference between the 95th and 5th percentiles of the voxel-based MD values within the skeleton.
LLS	Philips Achieva, 3.0 T scanner	The protocol included the following: 3DT1-weighted images: TR = 9.7 ms, TE = 4.6 ms, FA = 8°, FOV = 224 x 177 x 168 mm, resulting in a nominal voxel size of 1.17 x 1.17 x 1.4 mm, covering the entire brain with no gap between slices, acquisition time was approximately 5 minutes; T2-weighted images: TR = 4200 ms, TE = 80 ms, FA = 90°, FOV = 224 x 180 x 144 mm, matrix size 448 x 320, 40 transverse slices to cover the entire brain with a slice thickness of 3.6 mm with no gap between slices; FLAIR: TR = 11000 ms, TE = 125 ms, FA = 90°, FOV = 220 x 176 x 137 mm, matrix size 320 x 240, 25 transverse slices to cover the entire brain with a slice thickness of 5 mm with no gap between slices.	White matter lesion volume in milliliters was automatically quantified by using a previously validated method: In short, after initial tissue segmentation, white matter masks generated by FSL (FMRIB Software Library v5.0, Oxford GB)) were spatially transformed to fluid-attenuated inversion recovery (FLAIR) images by using the FLIRT tool. White matter hyperintensities were automatically identified from the mask by using a threshold of 3 standard deviations above the mean FLAIR signal intensity, which was obtained from the cerebral periphery to limit skewing of the signal intensity distribution from hyperintense periventricular white matter voxels.
LBC1936	GE Signa Horizon HDx 1.5T clinical scanner (General Electric, Milwaukee, WI, USA) equipped with a self-shielding gradient set (33 mT/m maximum gradient strength) and manufacturer supplied 8-channel	T1-w coronal and T2-W, FLAIR, and T2*-weighted axial whole brain images were obtained.	WMH were measured in the cerebral hemispheres, cerebellum and brainstem, by a semi-automatic computational program written specifically for the project,<br>MCMxxxVI, a multispectral color fusion method that combines different pairs of sequences in red-green color space and performs minimum variance quantization to highlight different tissues.[53]<br>Intracranial volume, brain and WMH volume were extracted and manually corrected as necessary to remove false positive lesions in the insular cortex, cingulate gyrus, anterior temporal cortex and around the floor of the third ventricle, and correct false negatives (http://www.bric.ed.ac.uk/research/imageanalysis.html). All focal

	phased-array head coil.		stroke lesions were manually removed.
MAS	Philips 3T Intera Quasar scanner (Philips Medical Systems, The Netherlands) for the first half of scans. This was replaced with a Philips 3T Achieva Quasar Dual scanner (Philips Medical System, The Netherlands), which was used for the rest of MAS participants.	T1-weighted, and T2-weighted FLAIR images were acquired. The scanning parameters used at the two scanners are identical:<br><br>T1-weighted MRI – TR = 6.39 ms, TE = 2.9 ms, flip angle = 8°, matrix size = 256 × 256, FOV = 256 × 256 × 190, and slice thickness = 1 mm with no gap in between, yielding 1 × 1 × 1 mm3isotropic voxels.<br><br>T2-weighted FLAIR – TR = 10000 ms, TE = 110 ms, TI = 2800 ms, matrix size = 512 × 512, slice thickness = 3.5 mm without gap, and in plane resolution = 0.488 × 0.488 mm.	White matter hyperintesnsities (WMH) volumes were calculated using UBO Detector.[54]
OATS	New South Wales study site: Philips 1.5T Gyroscan scanner was initially used, and later replaced with a Philips 3T Achieva Quasar Dual scanner.<br><br>Victoria study site: Siemens 1.5T Magnetom Avanto scanner.<br><br>Queensland study site: Siemens 1.5T Sonata scanner	T1-weighted MRI – T1-weighted images acquired from 1.5T scanners in all three centres: in-plane resolution 1 × 1 mm with slice thickness of 1.5 mm, contiguous slices, TR (repetition time) = 1530 ms, TE (echo time) = 3.24 ms, TI (inversion time) = 780 ms, and flip angle = 8°. Scanning parameters for T1-weighted MRI acquired on the 3T scanner in New South Wales: TR = 6.39 ms, TE = 2.9 ms, spatial resolution = 1 × 1 × 1 mm3.<br>T2-weighted FLAIR – FLAIR images acquired from 1.5T scanners in all three centres: TR = 10000 ms, TE = 120 ms, TI = 2800 ms, slice thickness = 3.5 mm, and in-plane resolution = 0.898 × 0.898 mm2. The scanning parameters of the 3T scanner at New South Wales: TR = 10000 ms, TE = 110 ms, TI = 2800 ms, slice thickness = 3.5 mm and in-plane resolution = 0.898 × 0.898 mm2.	White matter hyperintesnsities (WMH) volumes were calculated using UBO Detector.[54]
PROSPER	1.5 Tesla (Philips Medical Systems, Best, the Netherlands)	This segmentation was based on the T2-weighted and FLAIR images.	Tissue-type segmentation with partial volume estimation is carried out to calculate total volume of brain tissue (including separate estimates of volumes of gray matter, white matter, peripheral gray matter, and ventricular cerebrospinal fluid).[50] The algorithm FIRST (FMRIB's Integrated Registration and Segmentation Tool) was applied to estimate the volume of hippocampus, nucleus accumbens, globus pallidus, amygdala, putamen, caudate nucleus and thalamus. FIRST is part of FSL (FMRIB's Software Library) and performs both registration and segmentation of the mentioned subcortical regions. Segmentation of white matter hyperintensities volume was performed automatically using software for Neuro-Image Processing in Experimental Research (SNIPER), an in-house developed program for image processing.[55] This segmentation was based on the T2-weighted and FLAIR images.

RSI, RSII, RSIII	1.5 T scanner (GE Signa Excite) using an 8-channel head coil	Structural imaging is performed with T1-weighted (T1w), proton density-weighted (PDw) and fluid-attenuated inversion recovery (FLAIR) sequences.[31] The combination of different MR contrasts provided by these sequences can be used for automated brain tissue and white matter lesion segmentation. For this purpose, the T1w scan is acquired in 3D at high in-plane resolution and with thin slices (voxel size <1 mm3).	WMH volume was quantified using two fully automated methods, which was described previously in more detail (for RSI[56] and RSII/RSIII[31]). The former used the HASTE, PD and T2 sequences and the latter used the FLAIR, T1 and PD. Briefly, cerebrospinal fluid (CSF), gray matter (GM) and white matter (WM) are segmented by an atlas-based k-nearest neighbor classifier on multi-modal magnetic resonance imaging data. This classifier is trained by registering brain atlases to the subject. The resulting GM segmentation is used to automatically find a WMH threshold in a fluid-attenuated inversion recovery scan.
SHIP, SHIP-TREND	1.5-T MR imager (Magnetom Avanto ; Siemens Medical Systems, Erlangen, Germany)	T1, MP-RAGE/ axial plane, TR=1900 ms, TE=3.4 ms, Flip angle=15°, resolution of 1.0 x 1.0 x 1.0mm3 and T2 FLAIR / axial plane, TR= 5000, TE= 325, voxel= 0,9 x 0,9 x 3,0.	T1- and T2-weighted images of each subject were first aligned to each other using the AIR package. These images were then further analyzed with the optimized Voxel-Based Morphometry (VBM) protocol, using Statistical Parametric Mapping 99 (SPM99) that we modified in order to take into account the structural characteristics of the aged brain, as described in detail elsewhere. Fully automated image processing software was developed to detect, measure, and localize white matter hyperintensities (WMH).[38]
TASCOG	1.5T GE scanner	MRI scans were performed using a GE 1.5 Tesla scanner, with the following sequences; High resolution T1-weighted spoiled gradient echo (SPGR) MRI scans [TR 35ms, TE 7ms, flip angle 35°, field of view 24cm, voxel size = 1mm3] comprising 120 contiguous slices; Axial 3-dimensional T-2 weighted fast spin echo images (TR = 4300ms; TE = 106ms; NEX = 1; turbo factor = 48; voxel size = 3 mm3); Axial FLAIR (fluid attenuated inversion recovery) sequence (TR 8802, TE 125, TI 2200, 3mm contiguous thickness).	All scans were registered to a standard 152-brain Montreal Neurological Institute (MNI) template in stereotaxic coordinate space. Using T1 sequences and methods based on statistical parametric mapping software (SPM5), brain tissue was classified as gray matter, white matter, or cerebrospinal fluid. Fully automated morphological segmentation with adaptive boosting classification was applied to FLAIR and T1- and T2-weighted scans to identify WMH
3C Dijon	1.5-Tesla Magnetom scanner (Siemens, Erlangen, Germany).	T1- and T2-weighted images of each subject were first aligned to each other using the AIR package. These images were then further analyzed with the optimized Voxel-Based Morphometry (VBM) protocol, using Statistical Parametric Mapping 99 (SPM99) that we modified in order to take into account the structural characteristics of the aged brain, as described in detail elsewhere.	Fully automated image processing software was developed to detect, measure, and localize white matter hyperintensities (WMH).[38]
UKBB	3T Siemens Skyra, software VD13	The resulting imaging protocol included: three structural modalities, T1-weighted, T2-weighted and susceptibility-weighted MRI (referred to here as T1, T2 and swMRI); diffusion MRI (dMRI); and both task and resting-state functional MRI (tfMRI and rfMRI).	UKBB-I: Total volume of white matter hyperintensities (WMHs) was calculated with BIANCA[57] (using both T1 and T2 FLAIR)<br>UKBB-II: Total volume of WMHs was calculated using FreeSurfer (v6) using T1 images

* Except for the i-Share cohort in young adults where MRI-markers of integrity of white matter microstructure on diffusion tensor imaging were used instead of WMH burden
AGES: AGES-Reykjavik Study ; ARIC : Atherosclerosis Risk In Communities Study; ASPS: Austrian Stroke Prevention Study; ASPS-Fam: Austrian Stroke Prevention Family Study; CHS: Cardiovascular Health Study; CHAP: Chicago Health and Aging Project; CARDIA: Coronary Artery Risk Development in Young Adults; FHS: Framingham Heart Study; GENOA: Genetic Epidemiology Network of Arteriopathy; GeneSTAR: Genetic Studies of Atherosclerosis Risk; LLS: Leiden Longevity Study; LBC1936: Lothian Birth Cohort 1936; MAS: Sydney Memory and Ageing Study ; OATS: Older Australian Twins Study ; PROSPER: The Prospective Study on Pravastatin in the Elderly at Risk; RS: Rotterdam Study; SHIP/SHIP-TREND: Study of Health in Pomerania; TASCOG: ; 3C-Dijon: Three-City Dijon Study; UKBB: UK BioBank; i-Share

Supplementary table 2: Accessed GWAS summary statistics

TRAITS	ARTICLE	PMID	GW SUMMARY STATISTICS ACCESS
**DBP**	Evangelou E et al., Nature Genetics 2018 [58]	30429575	By application
**SBP**	Evangelou E et al., Nature Genetics 2018 [58]	30429575	By application
**PP**	Evangelou E et al., Nature Genetics 2018 [58]	30429575	By application
**HDL**	Willer CJ et al., Nature Genetics 2013 [59]	24097068	http://lipidgenetics.org/
**LDL**	Willer CJ et al., Nature Genetics 2013 [59]	24097068	http://lipidgenetics.org/
**TG**	Willer CJ et al., Nature Genetics 2013 [59]	24097068	http://lipidgenetics.org/
**T2D**	Xue A et al., Nature Communications 2018 [60]	30054458	http://cnsgenomics.com/data.html
**VTE**	Germain M et al., American Journal of Human genetics 2015 [61]	25772935	By application
**MIGRAINE**	Gormley P et al., Nature Genetics 2016 [62]	27322543	By application
**AS**	Malik R et al., Nature Genetics 2018 [63]	29531354	http://www.megastroke.org/index.html
**IS**	Malik R et al., Nature Genetics 2018 [63]	29531354	http://www.megastroke.org/index.html
**CE**	Malik R et al., Nature Genetics 2018 [63]	29531354	http://www.megastroke.org/index.html
**LAS**	Malik R et al., Nature Genetics 2018 [63]	29531354	http://www.megastroke.org/index.html
**SVS**	Malik R et al., Nature Genetics 2018 [63]	29531354	http://www.megastroke.org/index.html
**DEEP ICH**	Woo D et al., American Journal of Human genetics 2014 [64]	24656865	http://cerebrovascularportal.org/informational/downloads
**LOBAR ICH**	Woo D et al., American Journal of Human genetics 2014 [64]	24656865	http://cerebrovascularportal.org/informational/downloads
**GCF**	Davies G et al., Nature Communications 2018 [65]	29844566	http://www.ccace.ed.ac.uk/node/335
**AD**	Jansen IE et al., Nature Genetics 2019 [66]	30617256	https://ctg.cncr.nl/software/summary_statistics

Abbreviations: AS = All Stroke; IS = Ischemic Stroke; DBP = Diastolic Blood Pressure; BMI = Body Mass Index; SBP = Systolic Blood Pressure; SVS = Small Vessel Stroke; GCF = General Cognitive Function; VTE = Venous Thrombo Embolism; T2D = Type II Diabetes; ICH = Intracerebral Hemorrhage; PP = Pulse Pressure; CE = Cardio-Embolic stroke; AD = Alzheimer's Disease; LAS = Large Artery Stroke; LDL = Low-Density Lipoprotein; HDL = High-Density Lipoprotein; TG = triglycerides

Supplementary Figures

Supplementary Figure 1: Iceberg model on the pathomechanisms underlying cerebral small vessel disease (source of the base image: https://publicdomainvectors.org/en/free-clipart/Iceberg/83724.html)

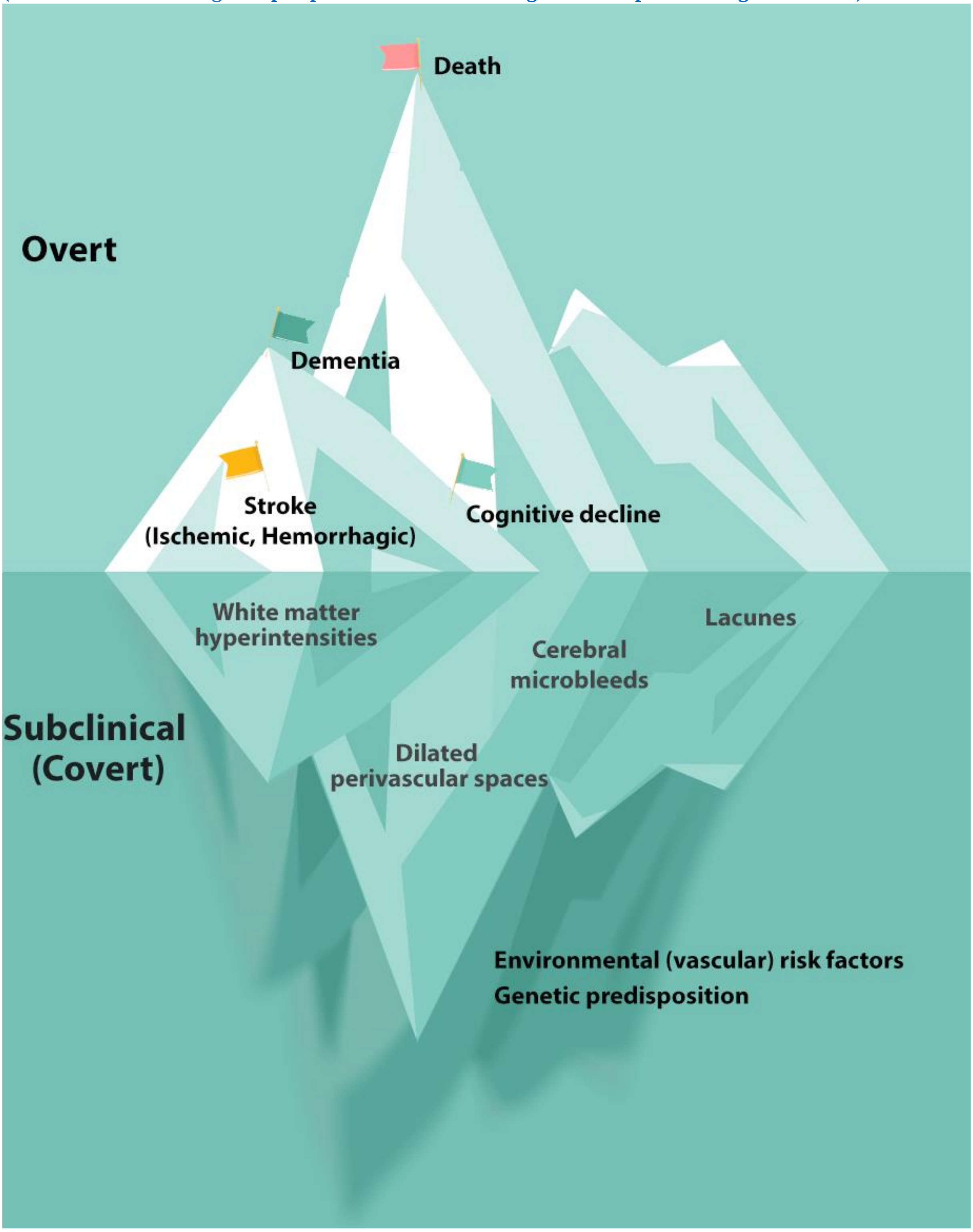

Supplementary Figure 2: QQ plot of genomic inflation factor for the different association models

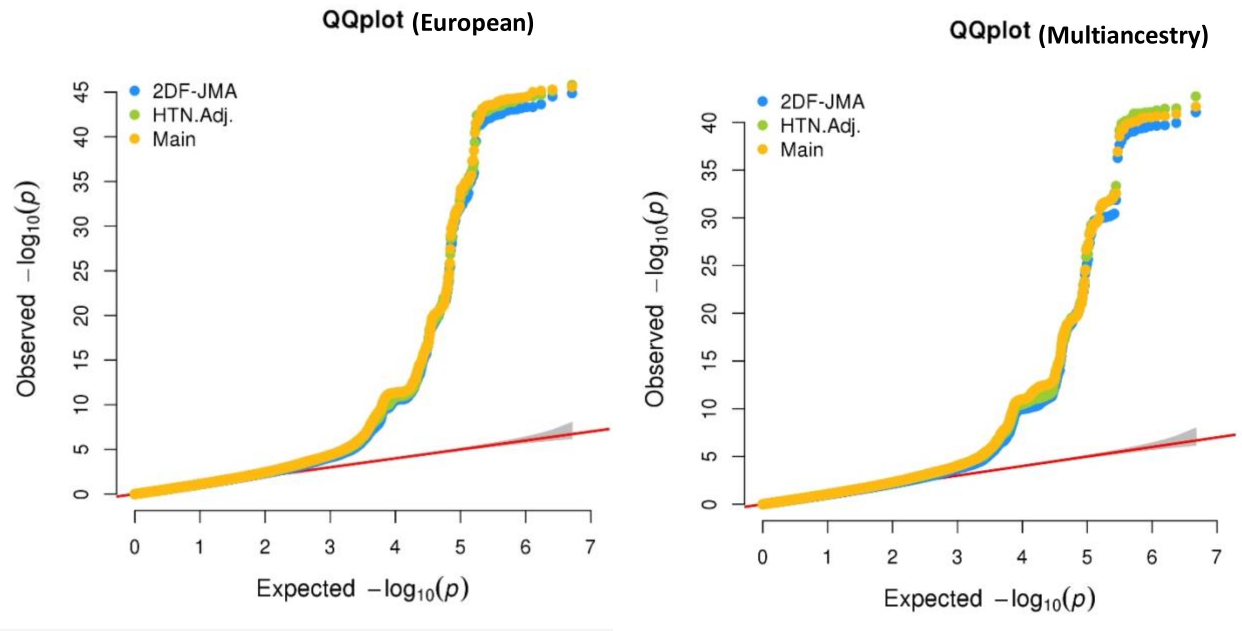

Main: main effects, HTN. Adj.: Hypertension adjusted main effects, 2DF-JMA: 2 degrees of freedom Joint meta-analysis

Supplementary Figure 3: Regional association plots

Regional plots encompass the region flanking (± 500kb) the lead WMH risk variant

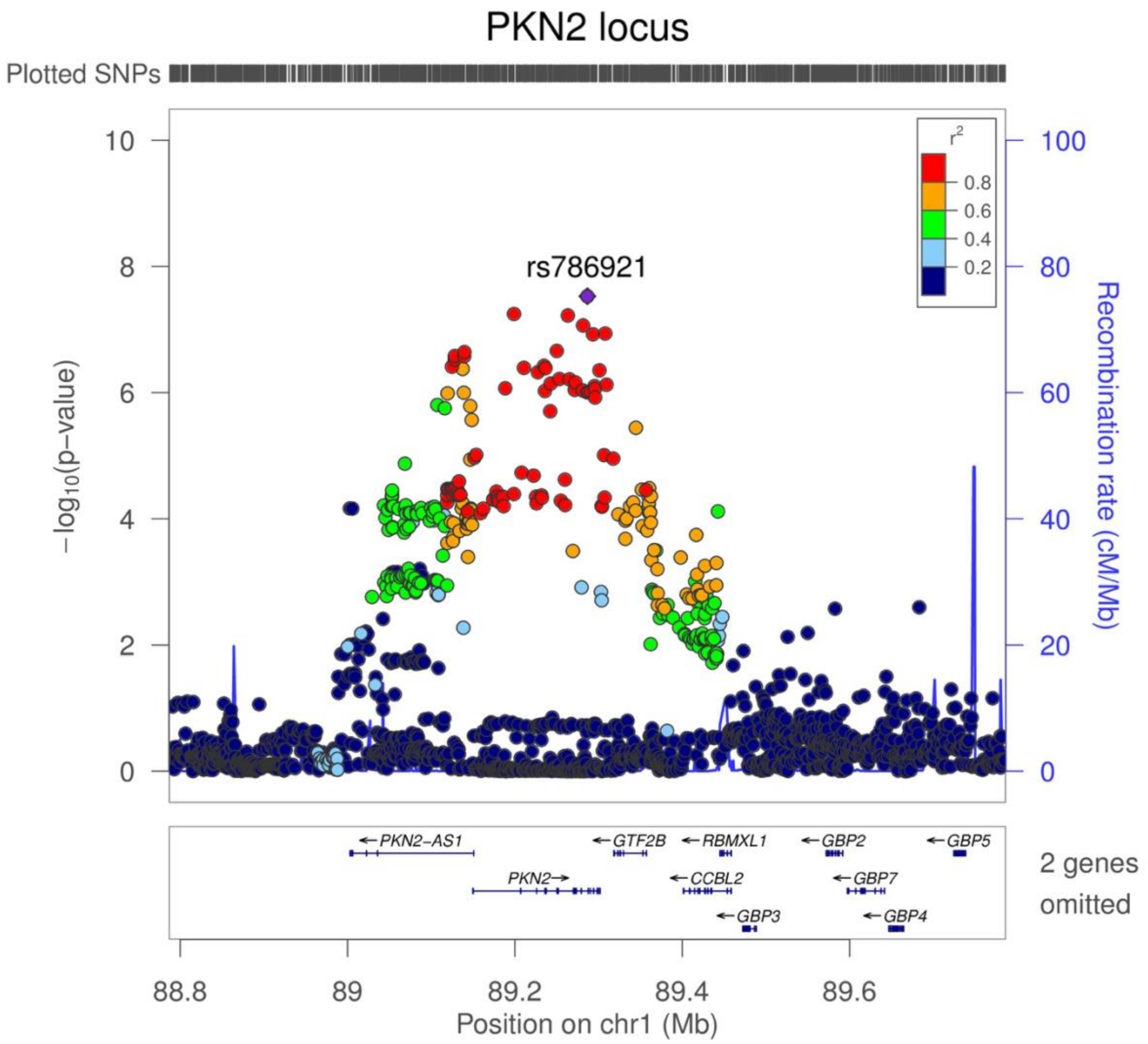

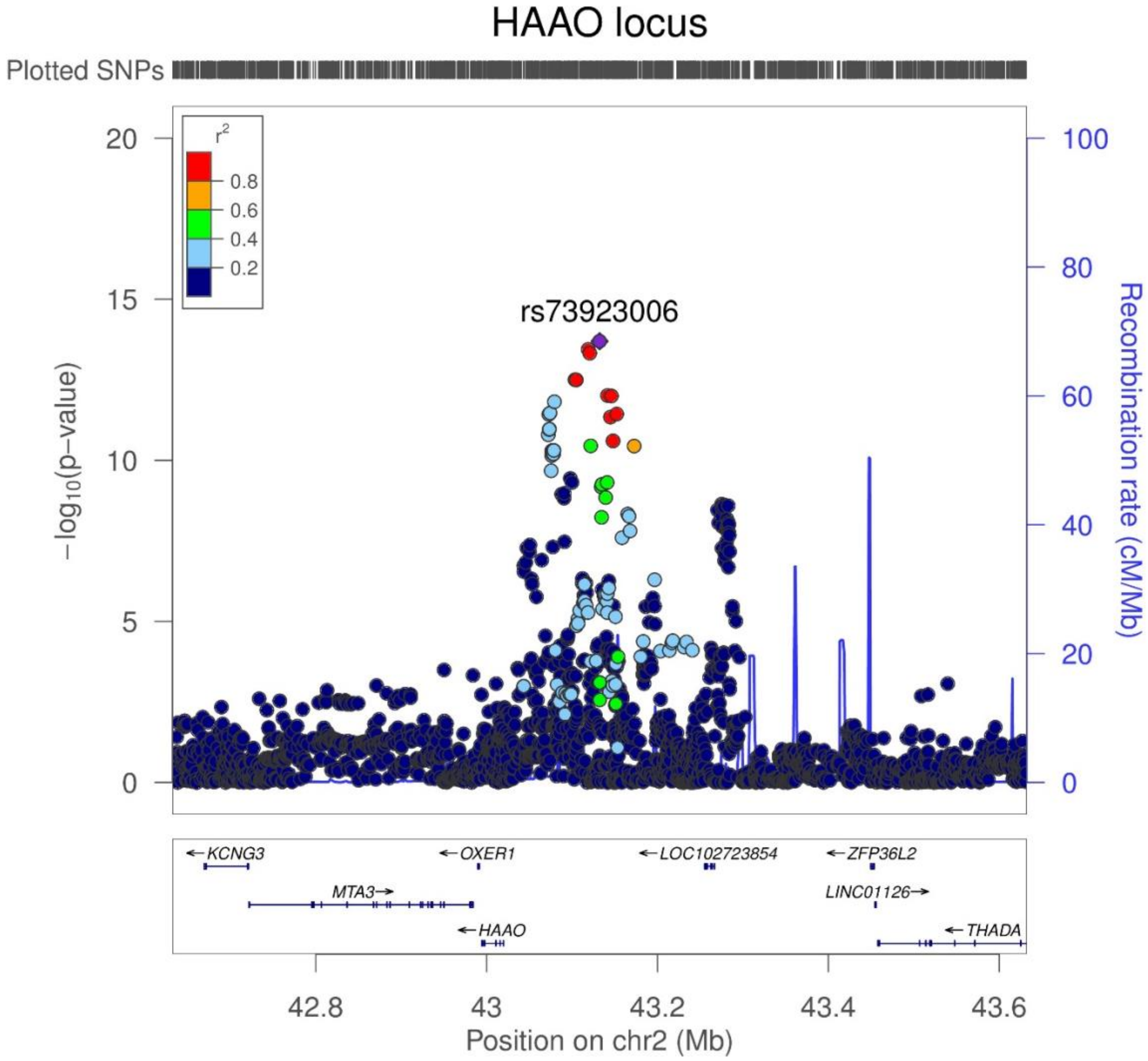

HAAO locus
Plotted SNPs
r²
0.8
0.6
0.4
0.2
rs73923006
−log10(p−value)
Recombination rate (cM/Mb)
20
15
10
5
0
100
80
60
40
20
0
KCNG3
OXER1
LOC102723854
ZFP36L2
MTA3
LINC01126
HAAO
THADA
42.8
43
43.2
43.4
43.6
Position on chr2 (Mb)

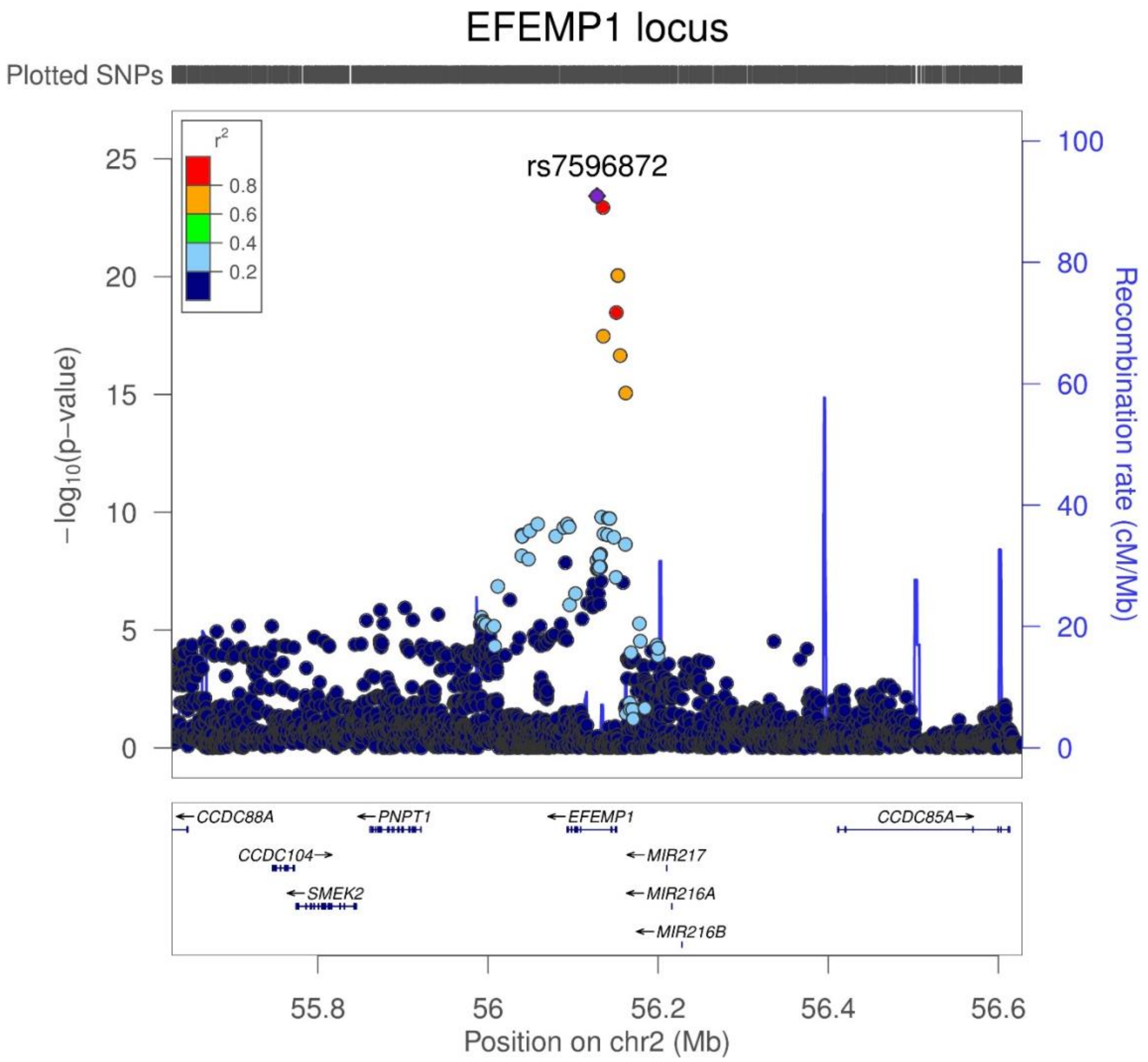
EFEMP1 locus
Plotted SNPs
rs7596872
r²
0.8
0.6
0.4
0.2
−log10(p−value)
Recombination rate (cM/Mb)
CCDC88A
PNPT1
EFEMP1
CCDC85A
CCDC104
MIR217
SMEK2
MIR216A
MIR216B
55.8
56
56.2
56.4
56.6
Position on chr2 (Mb)

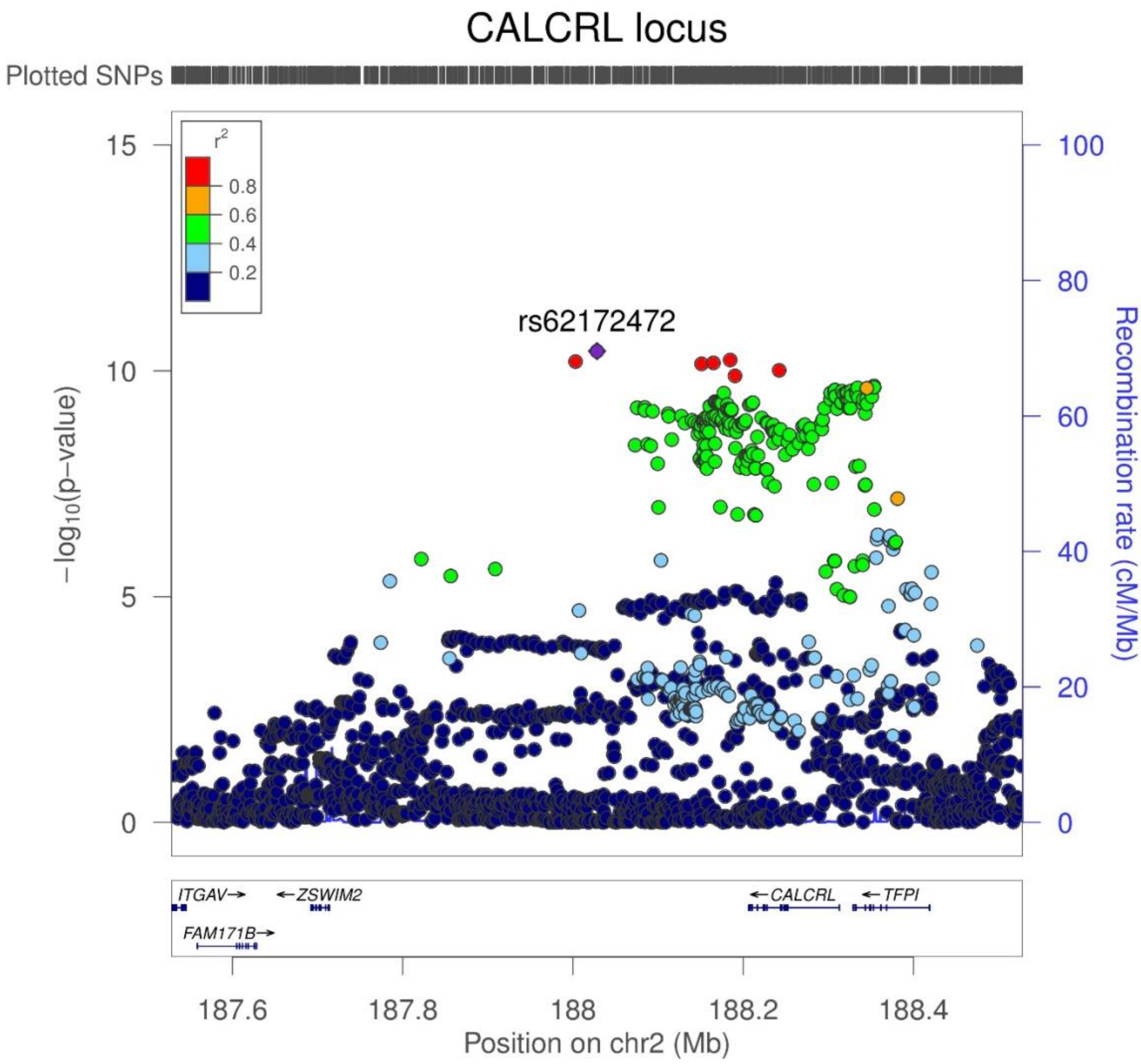

CALCRL locus
Plotted SNPs
r²
0.8
0.6
0.4
0.2
rs62172472
−log₁₀(p−value)
Recombination rate (cM/Mb)
15
10
5
0
100
80
60
40
20
ITGAV→
←ZSWIM2
FAM171B→
←CALCRL
←TFPI
187.6
187.8
188
188.2
188.4
Position on chr2 (Mb)

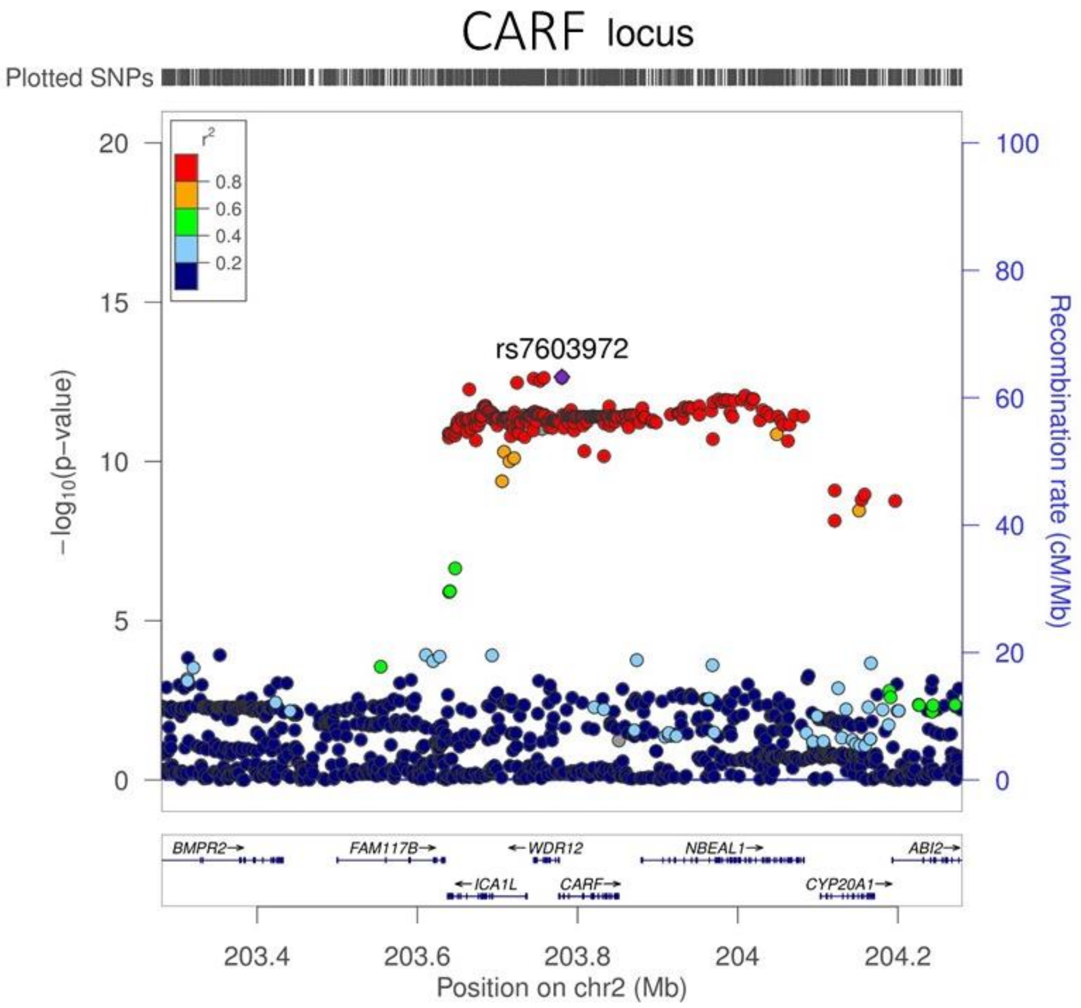
CARF locus
Plotted SNPs
r2
0.8
0.6
0.4
0.2
rs7603972
−log10(p−value)
Recombination rate (cM/Mb)
BMPR2→
FAM117B→
←WDR12
NBEAL1→
ABI2→
←ICA1L
CARF→
CYP20A1→
203.4
203.6
203.8
204
204.2
Position on chr2 (Mb)

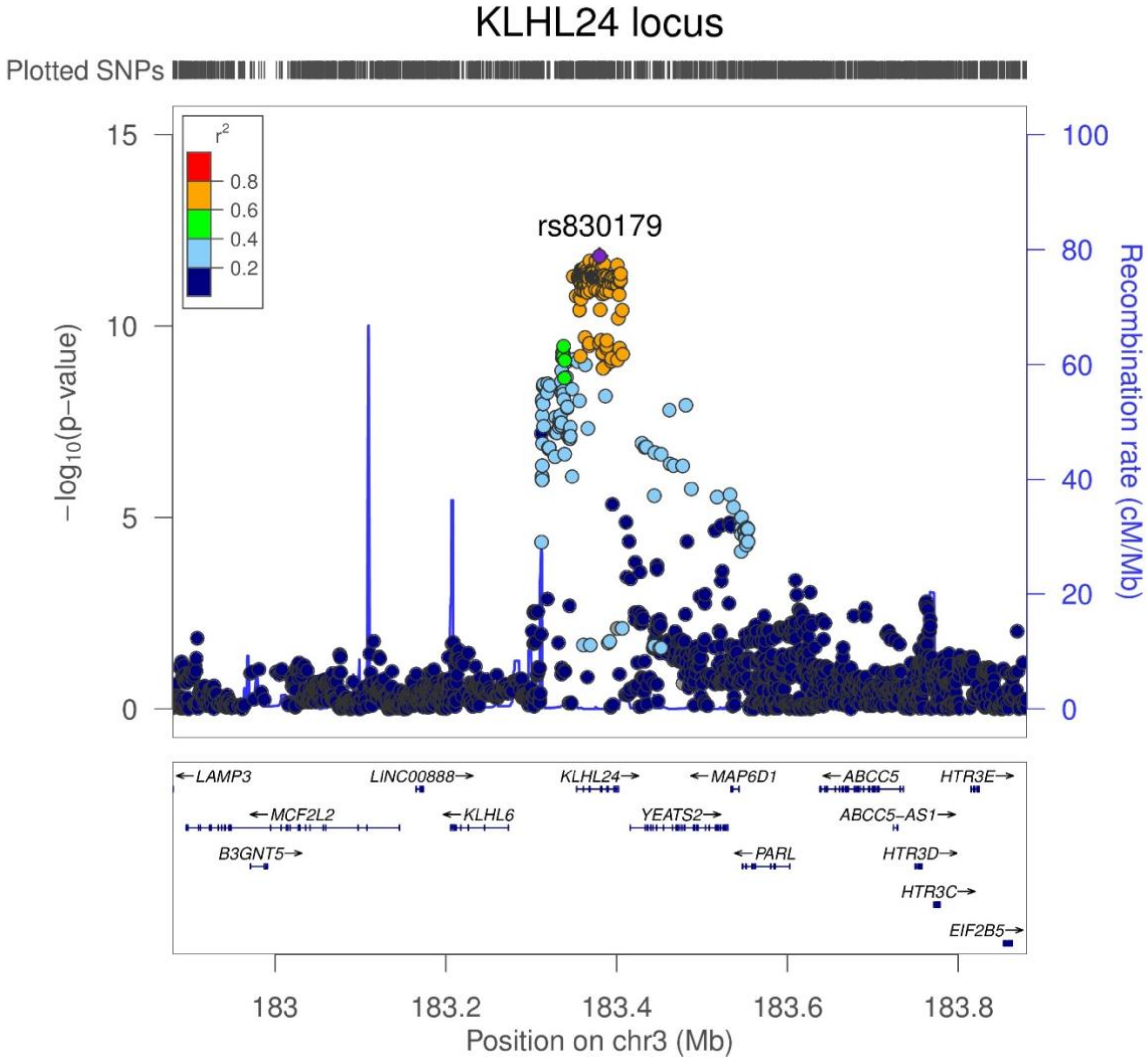
KLHL24 locus
Plotted SNPs
r²
0.8
0.6
0.4
0.2
rs830179
15
10
5
0
−log10(p−value)
100
80
60
40
20
0
Recombination rate (cM/Mb)
←LAMP3
LINC00888→
KLHL24→
←MAP6D1
←ABCC5
HTR3E→
←MCF2L2
←KLHL6
YEATS2→
ABCC5−AS1→
B3GNT5→
←PARL
HTR3D→
HTR3C→
EIF2B5→
183
183.2
183.4
183.6
183.8
Position on chr3 (Mb)

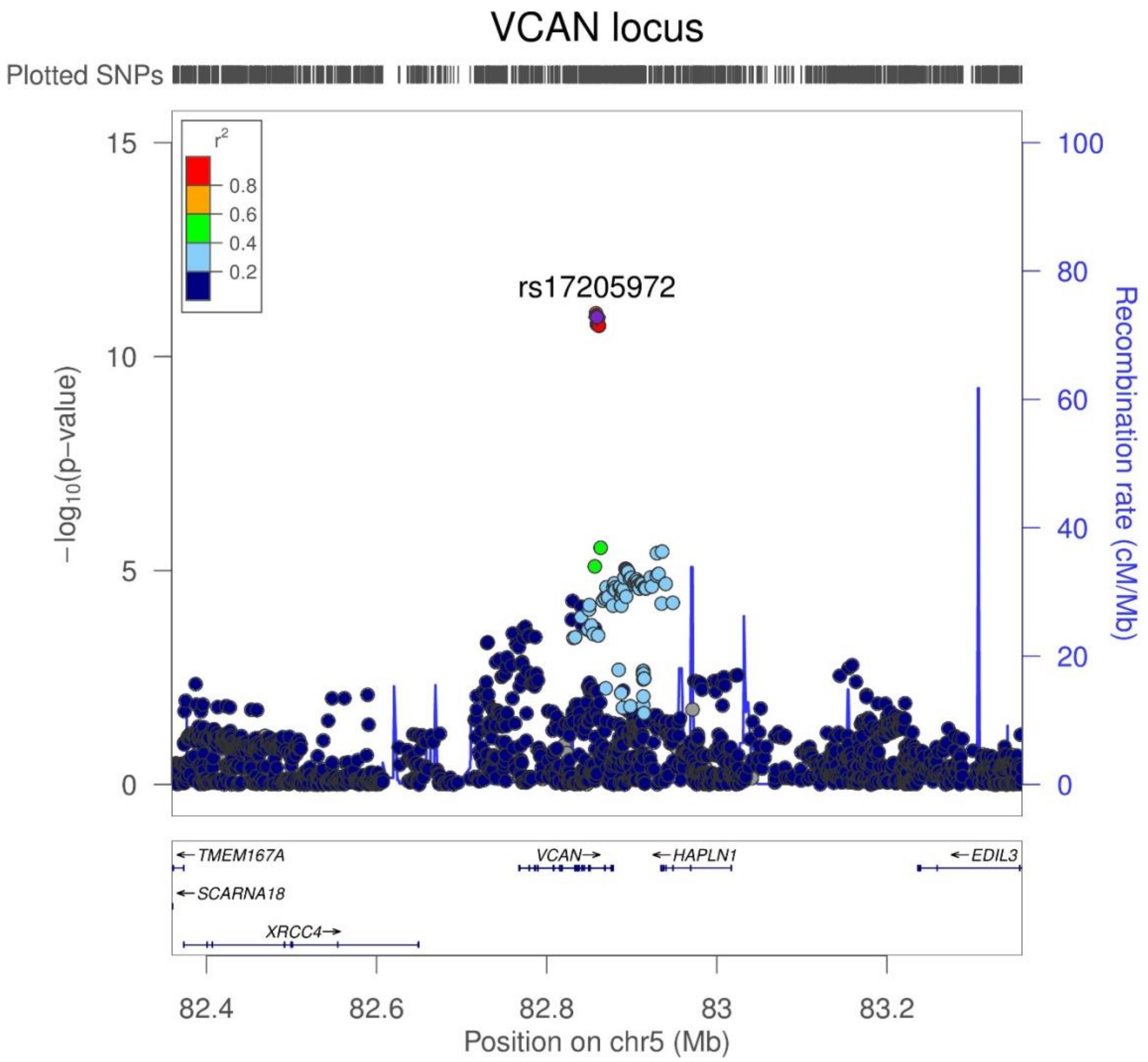
VCAN locus
Plotted SNPs
r²
0.8
0.6
0.4
0.2
rs17205972
−log10(p−value)
15
10
5
0
100
80
60
40
20
0
Recombination rate (cM/Mb)
TMEM167A
SCARNA18
XRCC4
VCAN
HAPLN1
EDIL3
82.4
82.6
82.8
83
83.2
Position on chr5 (Mb)

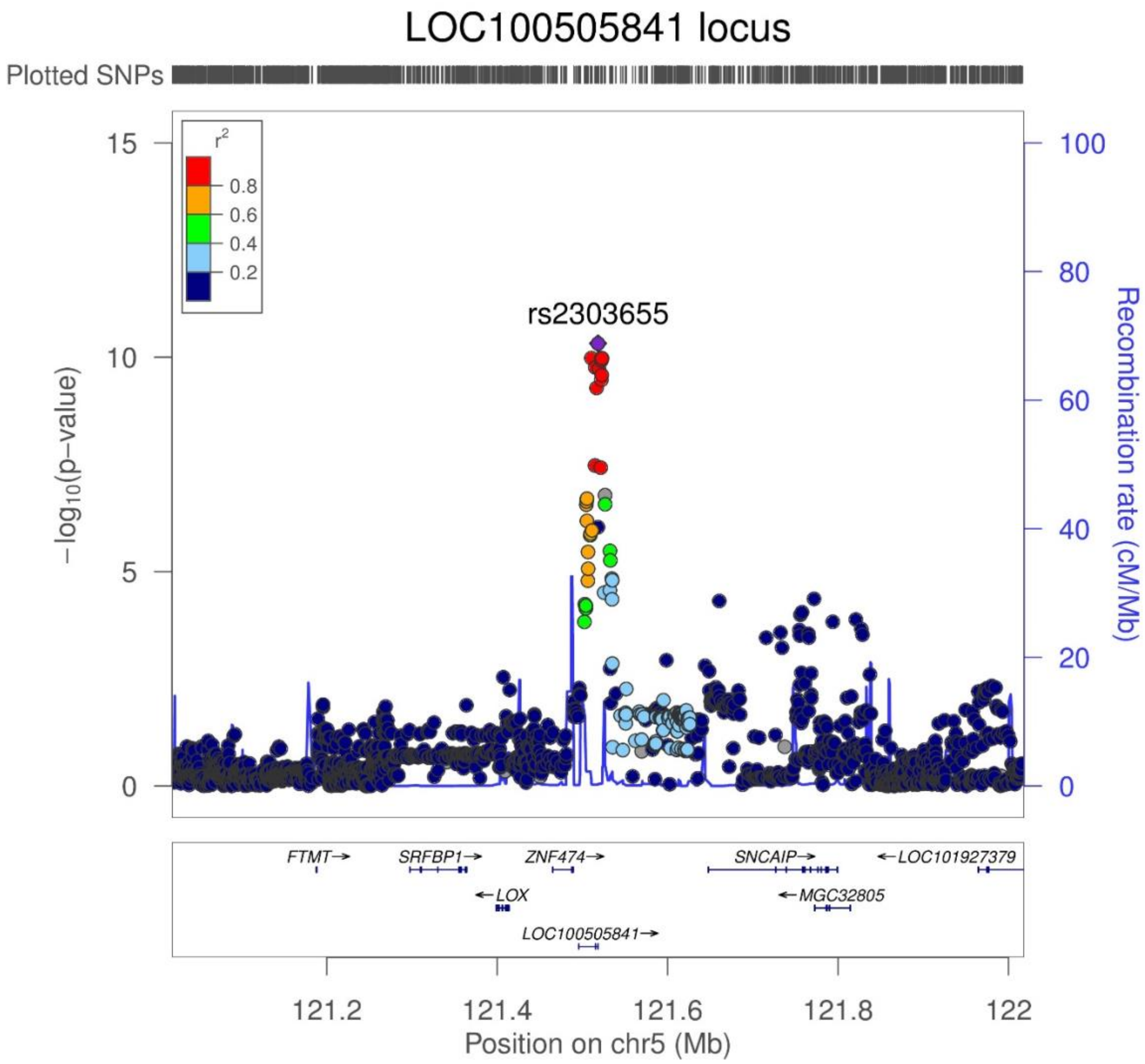
LOC100505841 locus
Plotted SNPs
−log10(p−value)
Recombination rate (cM/Mb)
r²
0.8
0.6
0.4
0.2
rs2303655
FTMT→
SRFBP1→
ZNF474→
SNCAIP→
←LOC101927379
←LOX
←MGC32805
LOC100505841→
Position on chr5 (Mb)

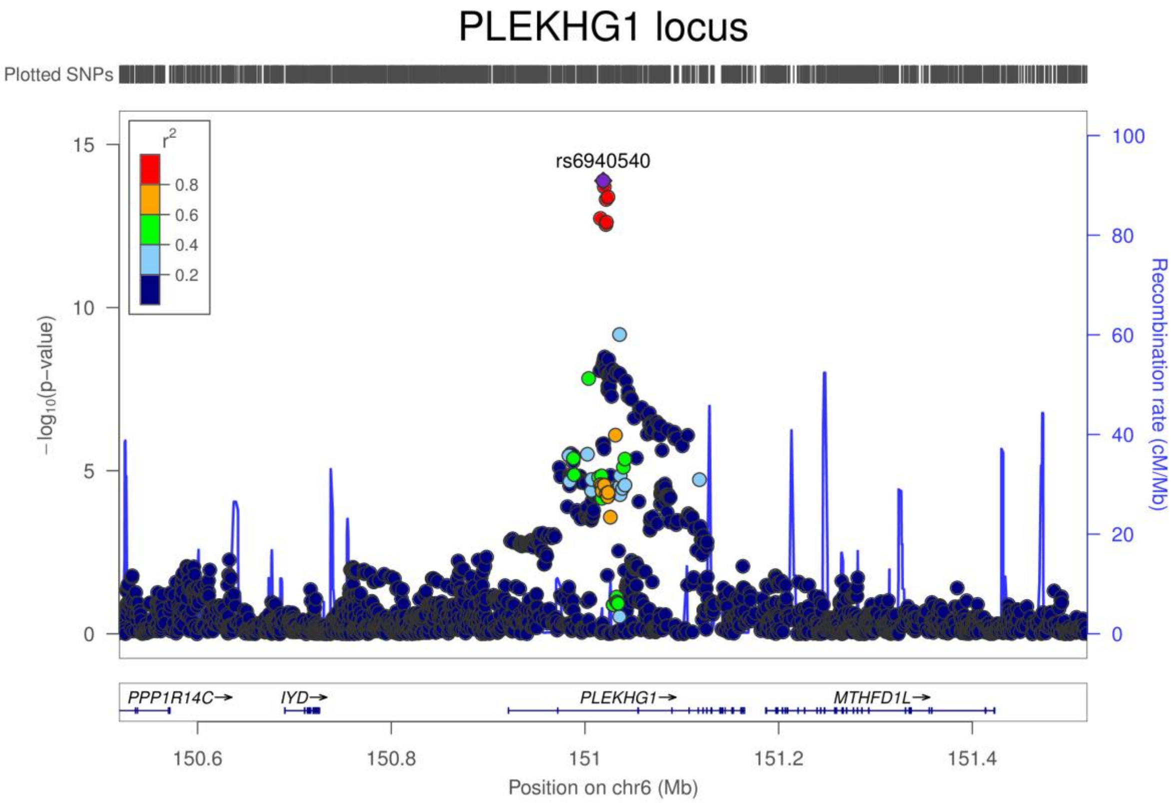

PLEKHG1 locus
Plotted SNPs
rs6940540
r²
0.8
0.6
0.4
0.2
−log10(p−value)
Recombination rate (cM/Mb)
PPP1R14C→
IYD→
PLEKHG1→
MTHFD1L→
150.6
150.8
151
151.2
151.4
Position on chr6 (Mb)

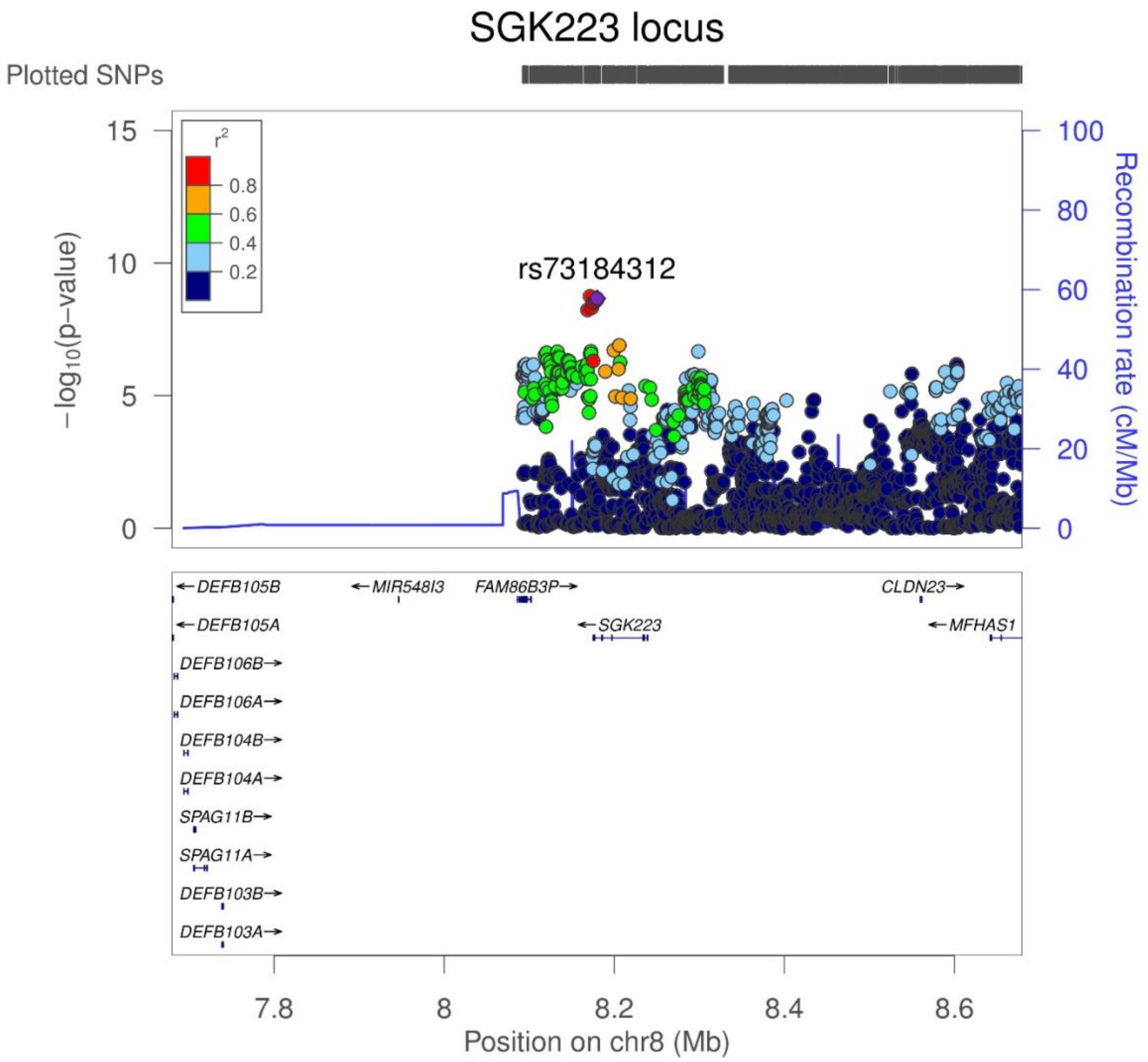

SGK223 locus
Plotted SNPs
−log10(p−value)
Recombination rate (cM/Mb)
r²
0.8
0.6
0.4
0.2
rs73184312
DEFB105B
DEFB105A
DEFB106B
DEFB106A
DEFB104B
DEFB104A
SPAG11B
SPAG11A
DEFB103B
DEFB103A
MIR548I3
FAM86B3P
SGK223
CLDN23
MFHAS1
7.8
8
8.2
8.4
8.6
Position on chr8 (Mb)

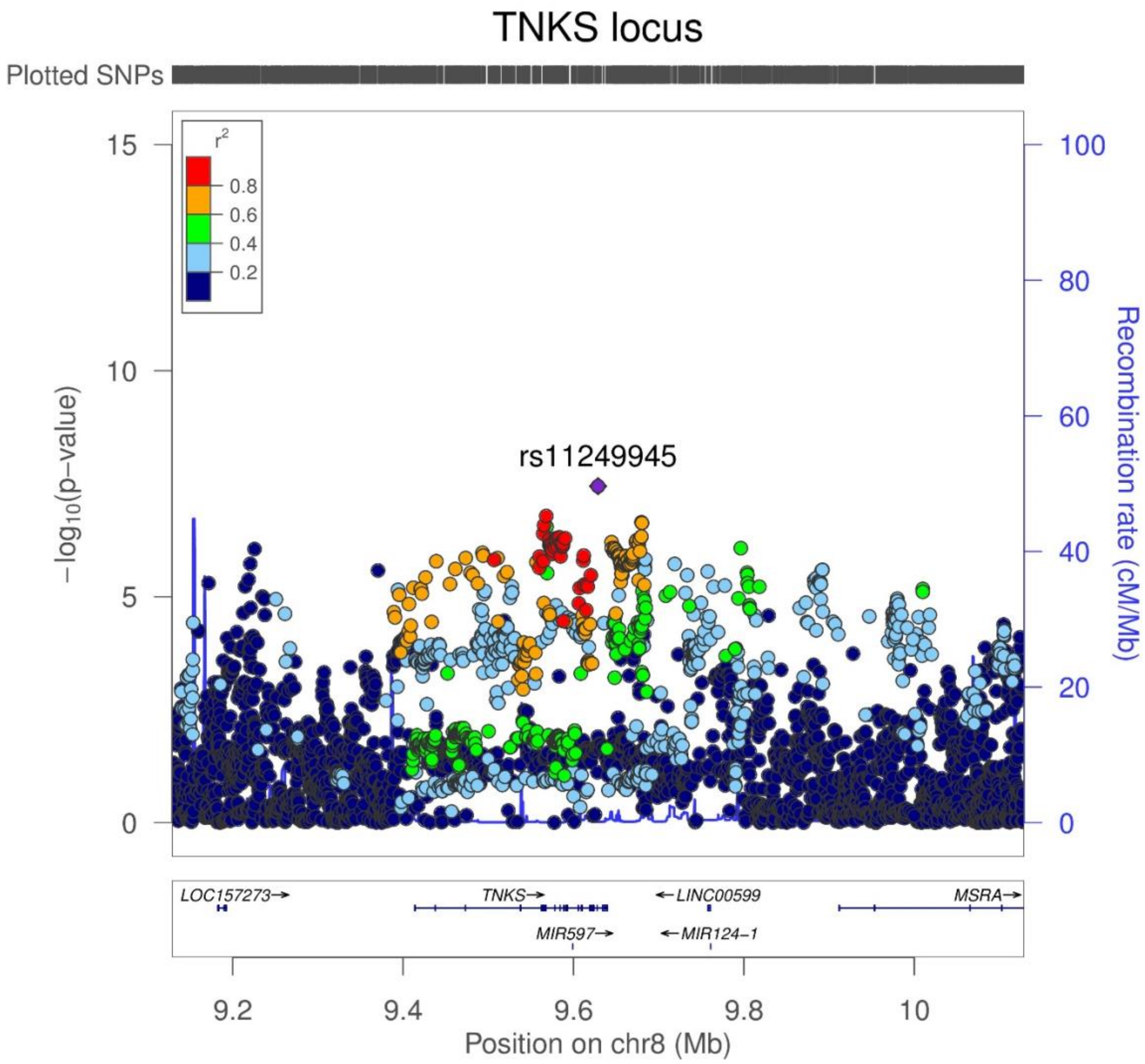
TNKS locus
Plotted SNPs
r2
0.8
0.6
0.4
0.2
rs11249945
−log10(p−value)
Recombination rate (cM/Mb)
LOC157273→
TNKS→
←LINC00599
MSRA→
MIR597→
←MIR124−1
9.2
9.4
9.6
9.8
10
Position on chr8 (Mb)

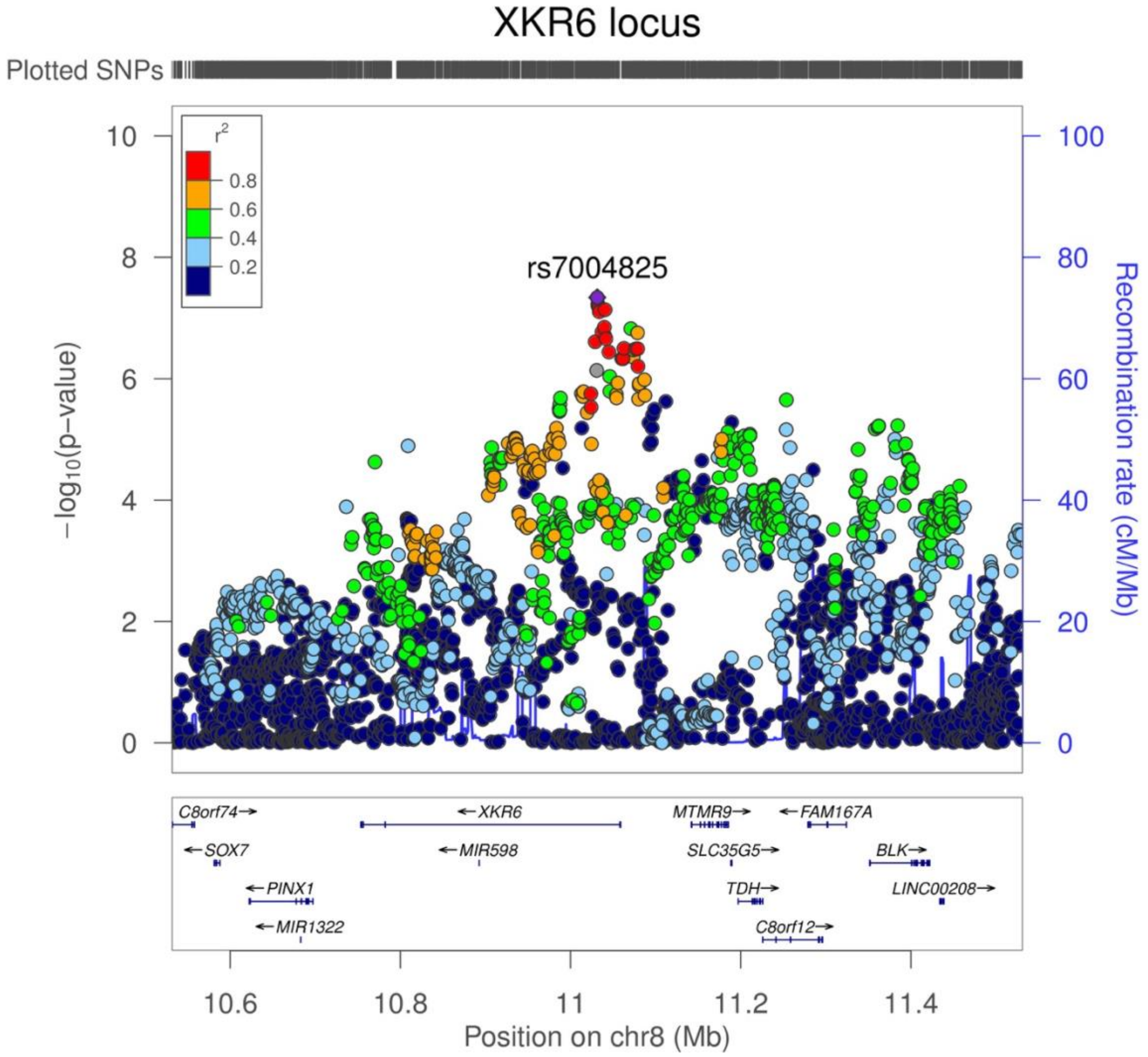
XKR6 locus
Plotted SNPs
rs7004825
r2
0.8
0.6
0.4
0.2
−log10(p−value)
Recombination rate (cM/Mb)
C8orf74
XKR6
MTMR9
FAM167A
SOX7
MIR598
SLC35G5
BLK
PINX1
TDH
LINC00208
MIR1322
C8orf12
Position on chr8 (Mb)

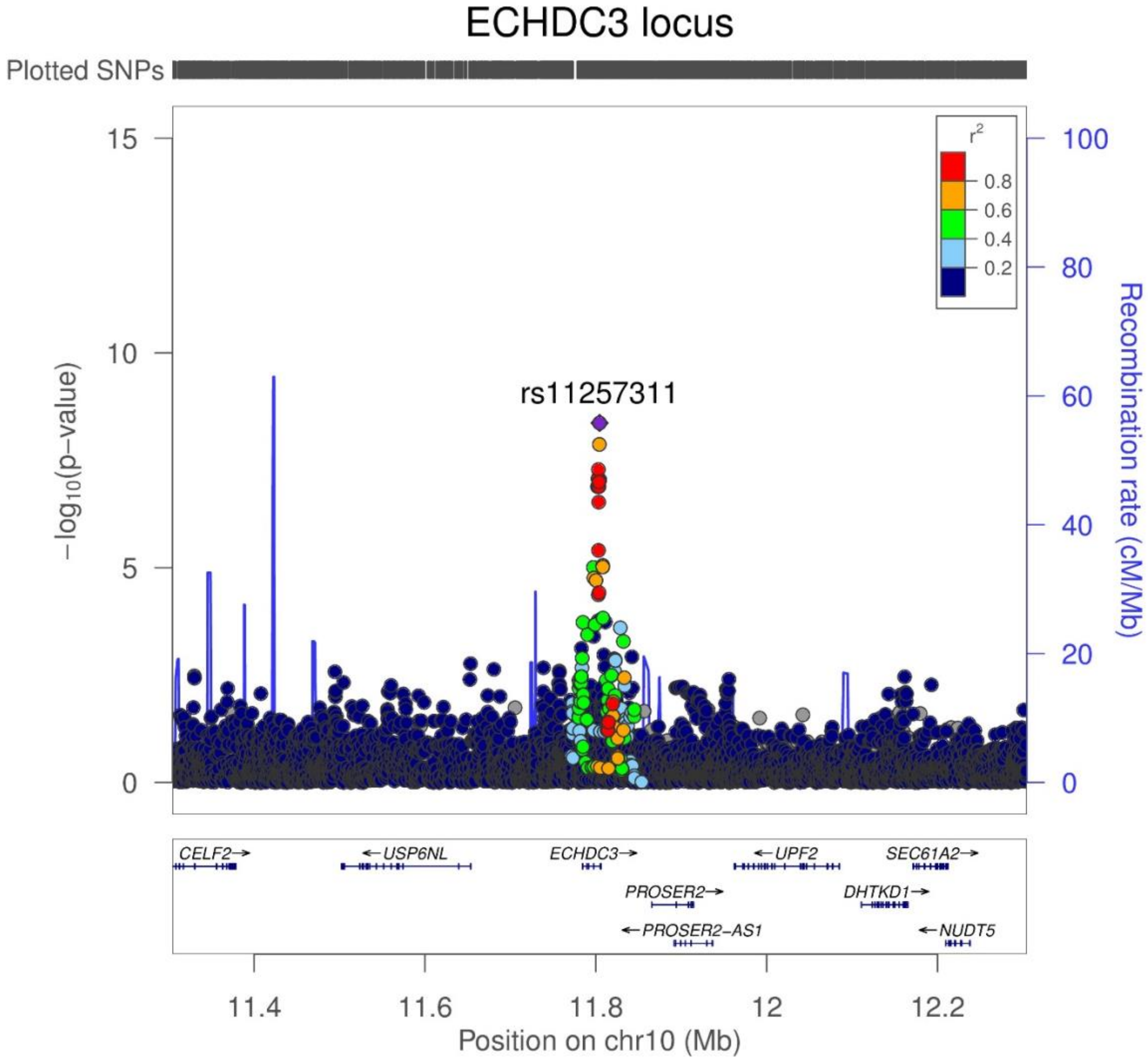
ECHDC3 locus
Plotted SNPs
−log10(p−value)
Recombination rate (cM/Mb)
r2
0.8
0.6
0.4
0.2
rs11257311
CELF2→
←USP6NL
ECHDC3→
←UPF2
SEC61A2→
PROSER2→
DHTKD1→
←PROSER2-AS1
←NUDT5
11.4
11.6
11.8
12
12.2
Position on chr10 (Mb)

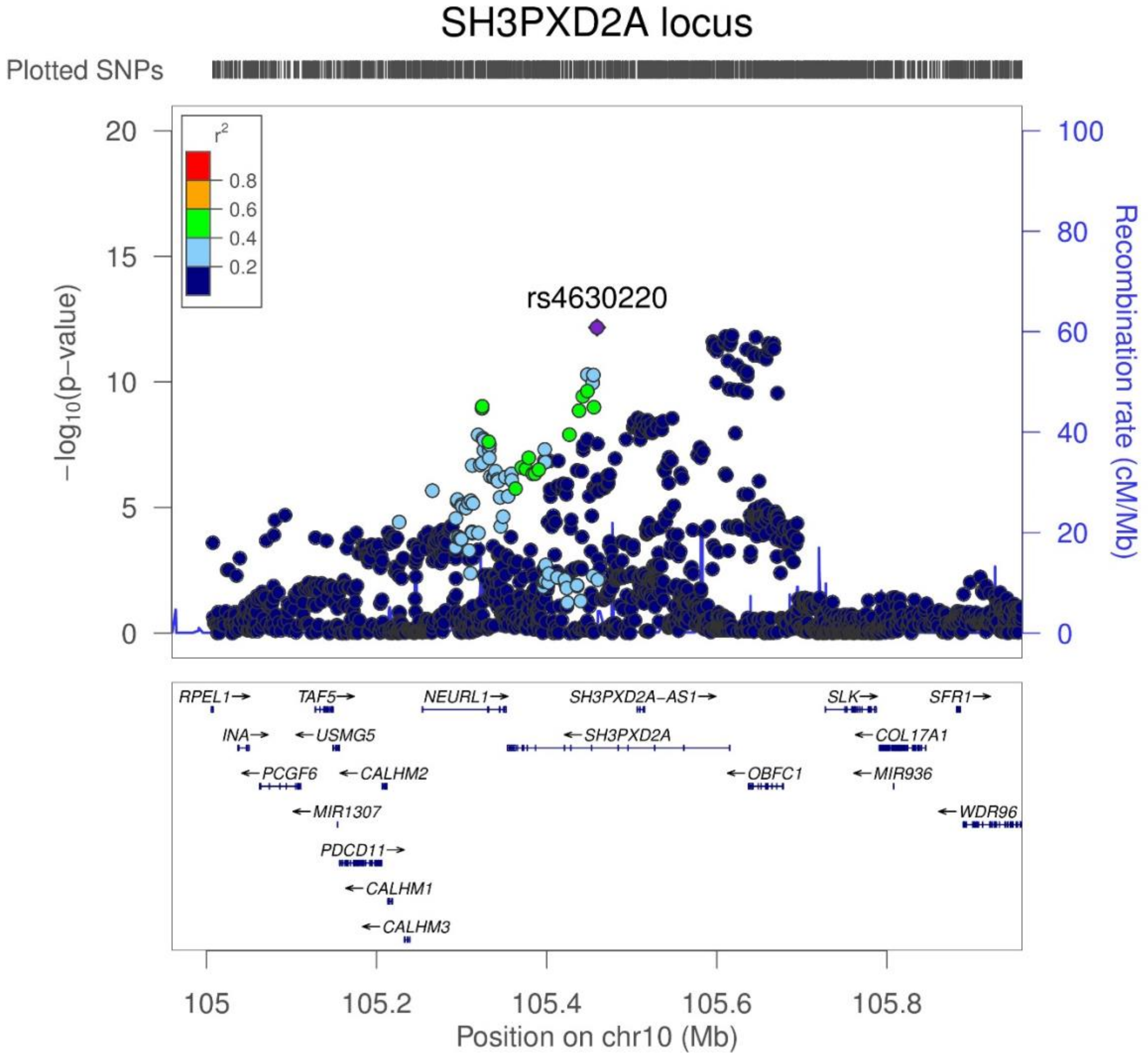
SH3PXD2A locus
Plotted SNPs
r2
0.8
0.6
0.4
0.2
rs4630220
20
15
10
5
0
−log10(p−value)
100
80
60
40
20
0
Recombination rate (cM/Mb)
RPEL1→
TAF5→
NEURL1→
SH3PXD2A−AS1→
SLK→
SFR1→
INA→
←USMG5
←SH3PXD2A
←COL17A1
←PCGF6
←CALHM2
←OBFC1
←MIR936
←MIR1307
←WDR96
PDCD11→
←CALHM1
←CALHM3
105
105.2
105.4
105.6
105.8
Position on chr10 (Mb)

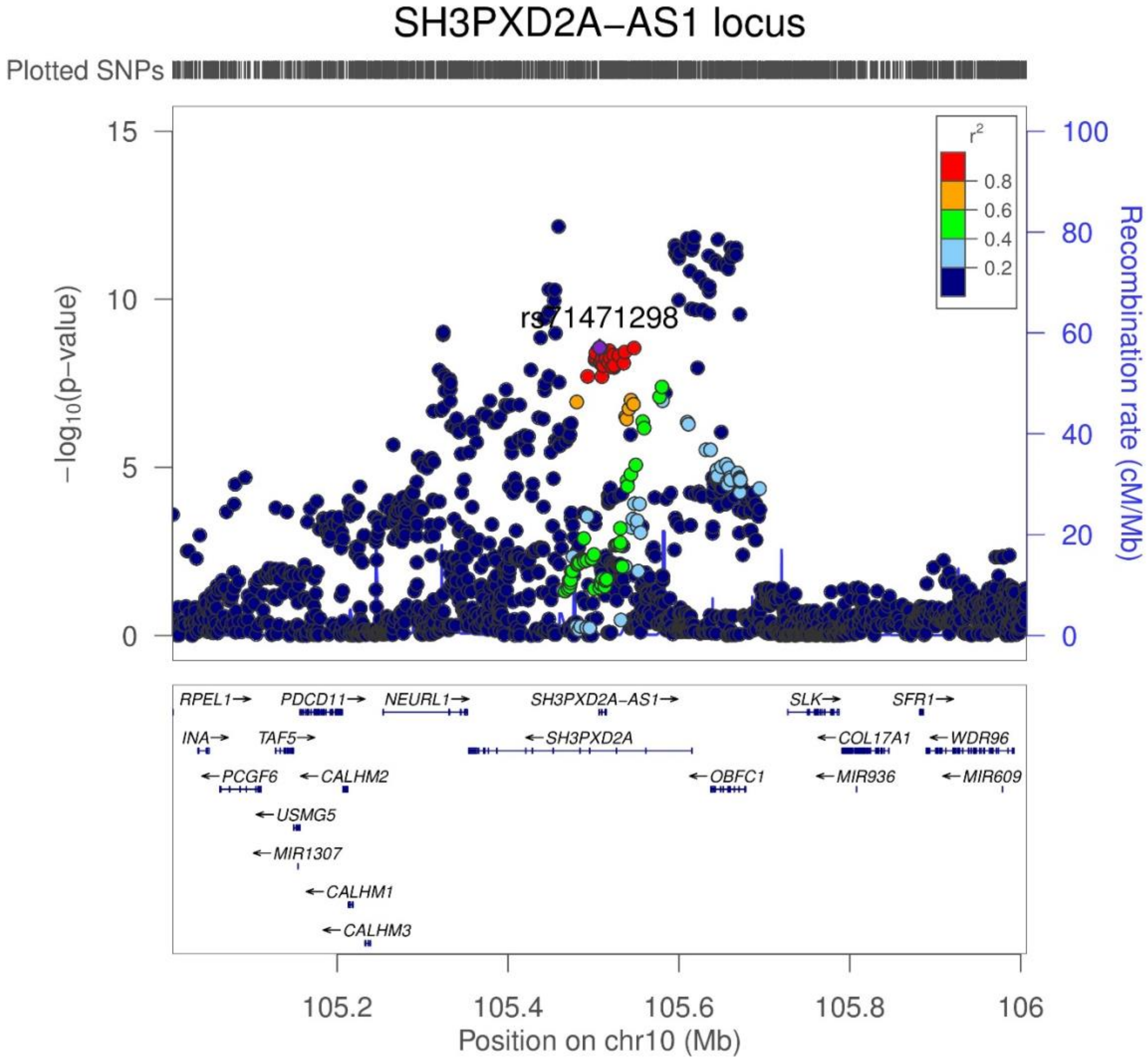

SH3PXD2A−AS1 locus
Plotted SNPs
−log10(p−value)
Recombination rate (cM/Mb)
r2
0.8
0.6
0.4
0.2
rs71471298
15
10
5
0
100
80
60
40
20
0
RPEL1→
PDCD11→
NEURL1→
SH3PXD2A−AS1→
SLK→
SFR1→
INA→
TAF5→
←SH3PXD2A
←COL17A1
←WDR96
←PCGF6
←CALHM2
←OBFC1
←MIR936
←MIR609
←USMG5
←MIR1307
←CALHM1
←CALHM3
105.2
105.4
105.6
105.8
106
Position on chr10 (Mb)

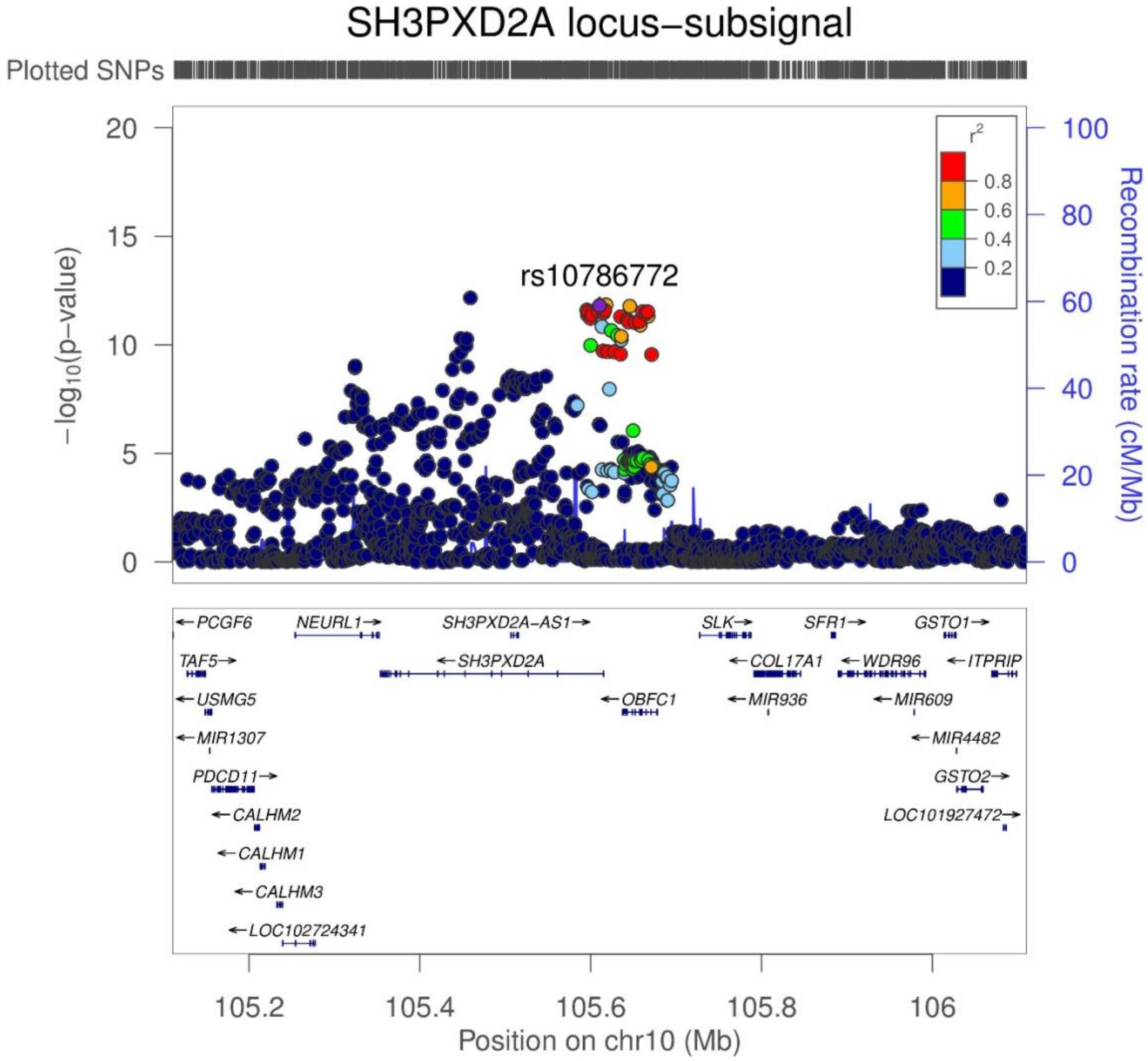
SH3PXD2A locus−subsignal
Plotted SNPs
rs10786772
−log10(p−value)
Recombination rate (cM/Mb)
r²
0.8
0.6
0.4
0.2
PCGF6
NEURL1
SH3PXD2A-AS1
SLK
SFR1
GSTO1
TAF5
SH3PXD2A
COL17A1
WDR96
ITPRIP
USMG5
OBFC1
MIR936
MIR609
MIR1307
MIR4482
PDCD11
GSTO2
CALHM2
LOC101927472
CALHM1
CALHM3
LOC102724341
105.2
105.4
105.6
105.8
106
Position on chr10 (Mb)

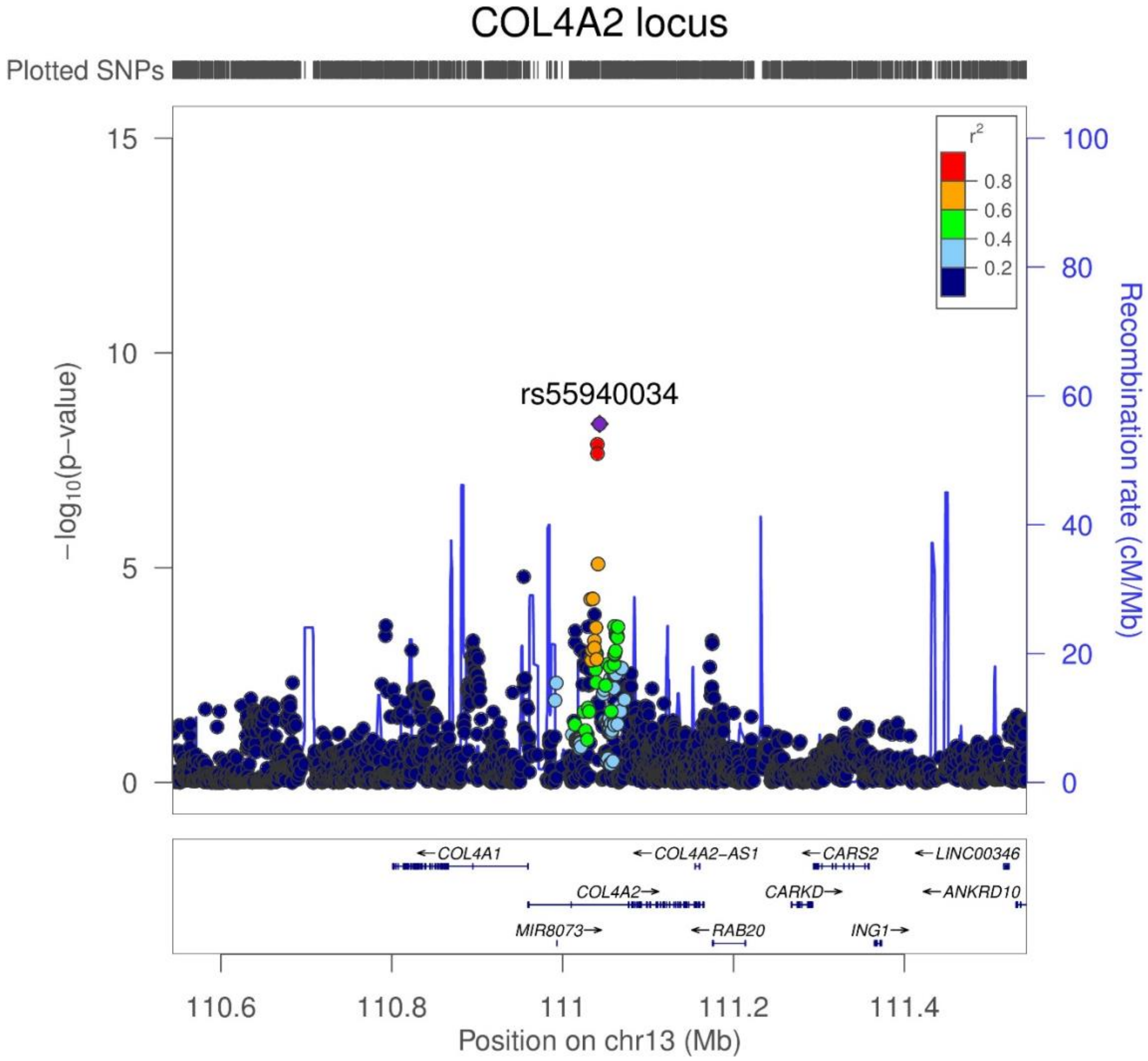
COL4A2 locus
Plotted SNPs
$-\log_{10}$(p-value)
15
10
5
0
r^2
0.8
0.6
0.4
0.2
100
80
60
40
20
0
Recombination rate (cM/Mb)
rs55940034
←COL4A1
←COL4A2-AS1
←CARS2
←LINC00346
COL4A2→
CARKD→
←ANKRD10
MIR8073→
←RAB20
ING1→
110.6
110.8
111
111.2
111.4
Position on chr13 (Mb)

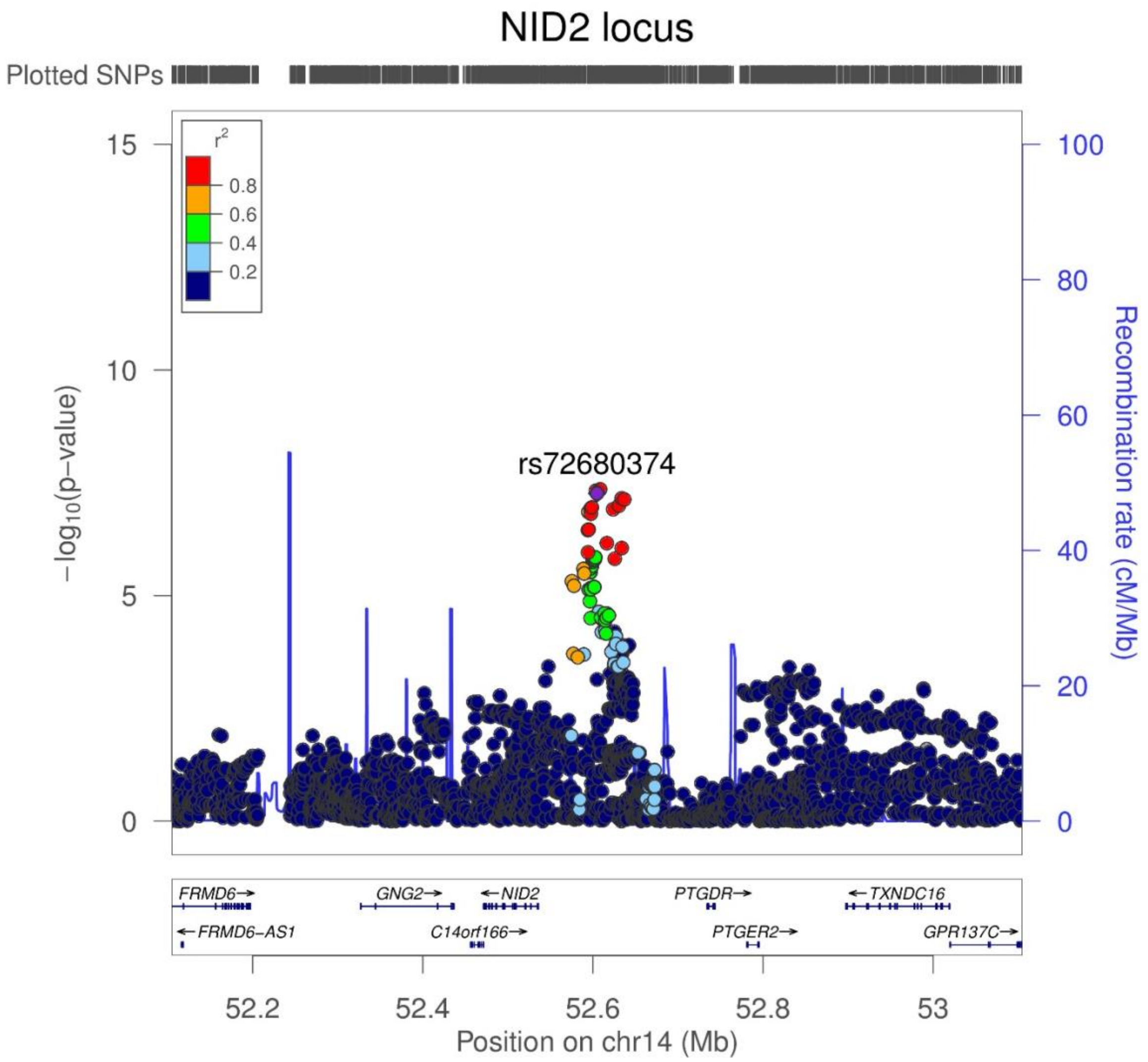

NID2 locus
Plotted SNPs
r²
0.8
0.6
0.4
0.2
15
10
5
0
100
80
60
40
20
0
−log10(p−value)
Recombination rate (cM/Mb)
rs72680374
FRMD6→
←FRMD6−AS1
GNG2→
C14orf166→
←NID2
PTGDR→
PTGER2→
←TXNDC16
GPR137C→
52.2
52.4
52.6
52.8
53
Position on chr14 (Mb)

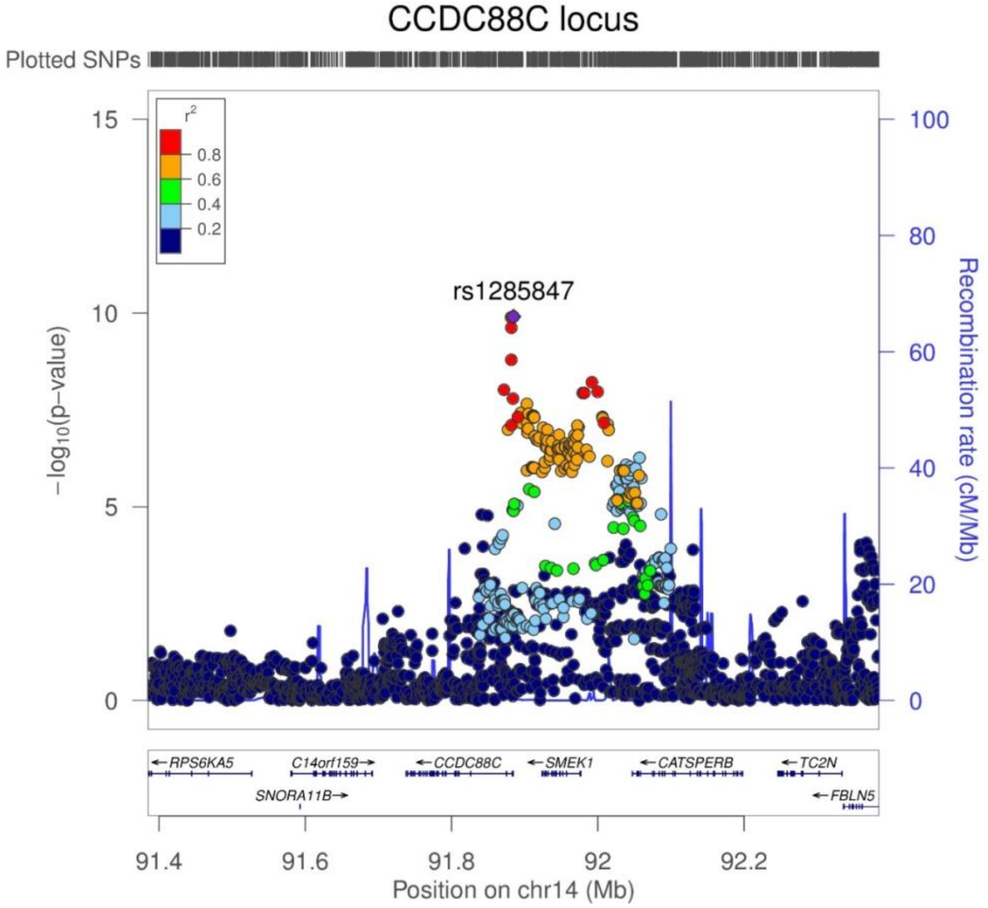
CCDC88C locus
Plotted SNPs
r²
0.8
0.6
0.4
0.2
rs1285847
−log10(p−value)
Recombination rate (cM/Mb)
15
10
5
0
100
80
60
40
20
0
RPS6KA5
C14orf159
CCDC88C
SMEK1
CATSPERB
TC2N
SNORA11B
FBLN5
91.4
91.6
91.8
92
92.2
Position on chr14 (Mb)

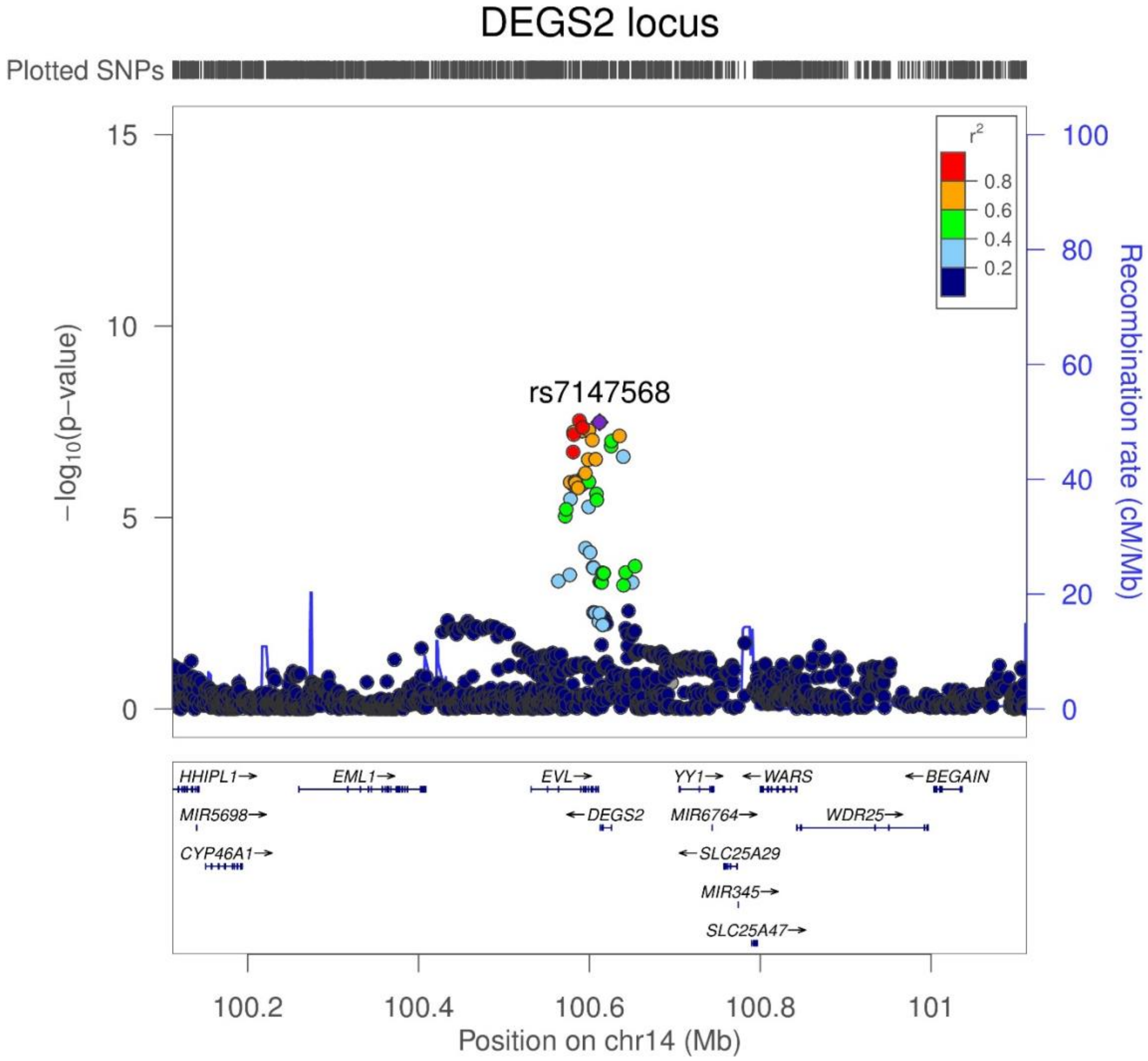

DEGS2 locus
Plotted SNPs
−log10(p−value)
Recombination rate (cM/Mb)
rs7147568
r2
0.8
0.6
0.4
0.2
HHIPL1→
EML1→
EVL→
YY1→
←WARS
←BEGAIN
MIR5698→
←DEGS2
MIR6764→
WDR25→
CYP46A1→
←SLC25A29
MIR345→
SLC25A47→
100.2
100.4
100.6
100.8
101
Position on chr14 (Mb)

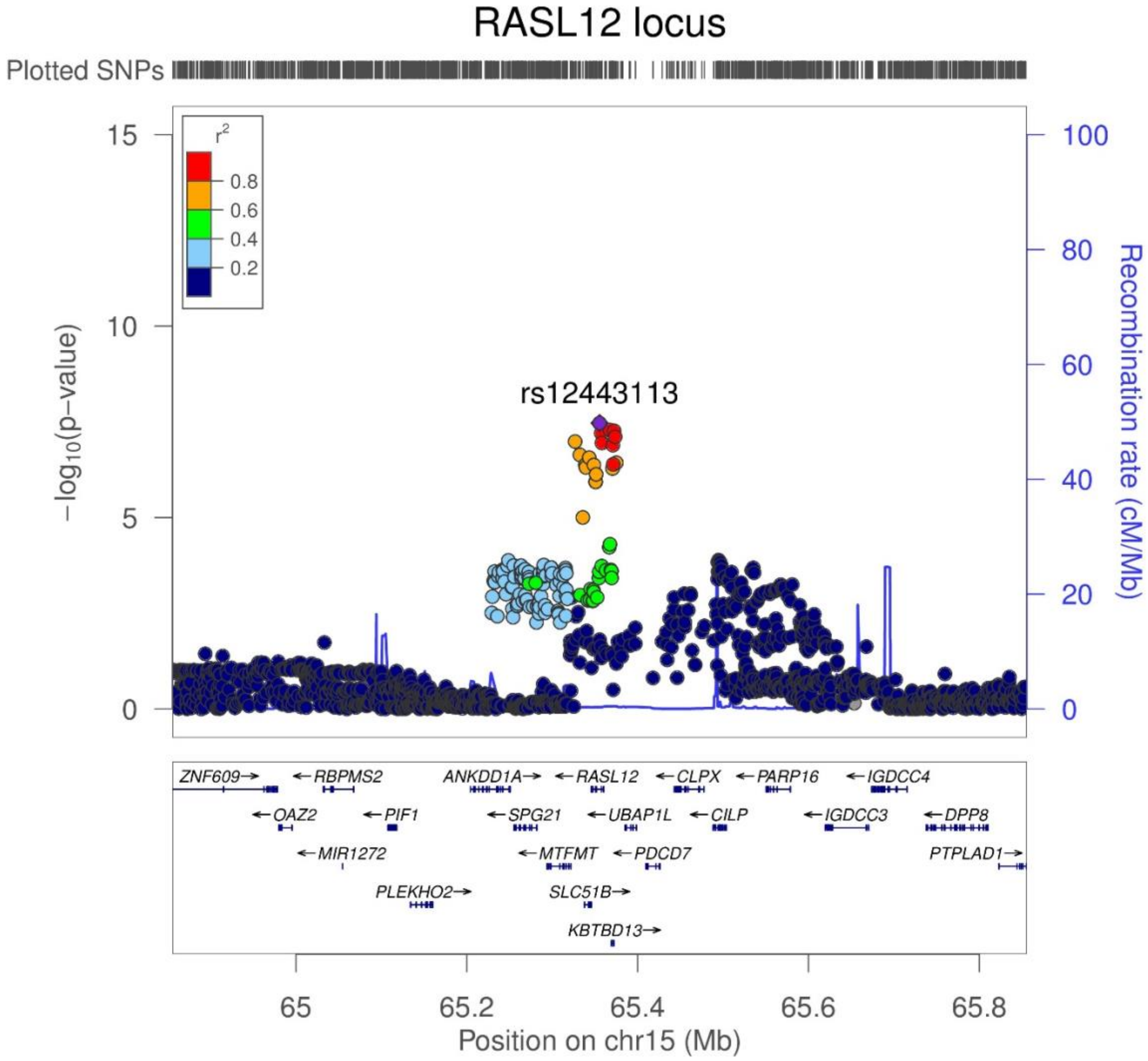
RASL12 locus
Plotted SNPs
rs12443113
−log10(p−value)
Recombination rate (cM/Mb)
r2
0.8
0.6
0.4
0.2
ZNF609
RBPMS2
ANKDD1A
RASL12
CLPX
PARP16
IGDCC4
OAZ2
PIF1
SPG21
UBAP1L
CILP
IGDCC3
DPP8
MIR1272
MTFMT
PDCD7
PTPLAD1
PLEKHO2
SLC51B
KBTBD13
Position on chr15 (Mb)

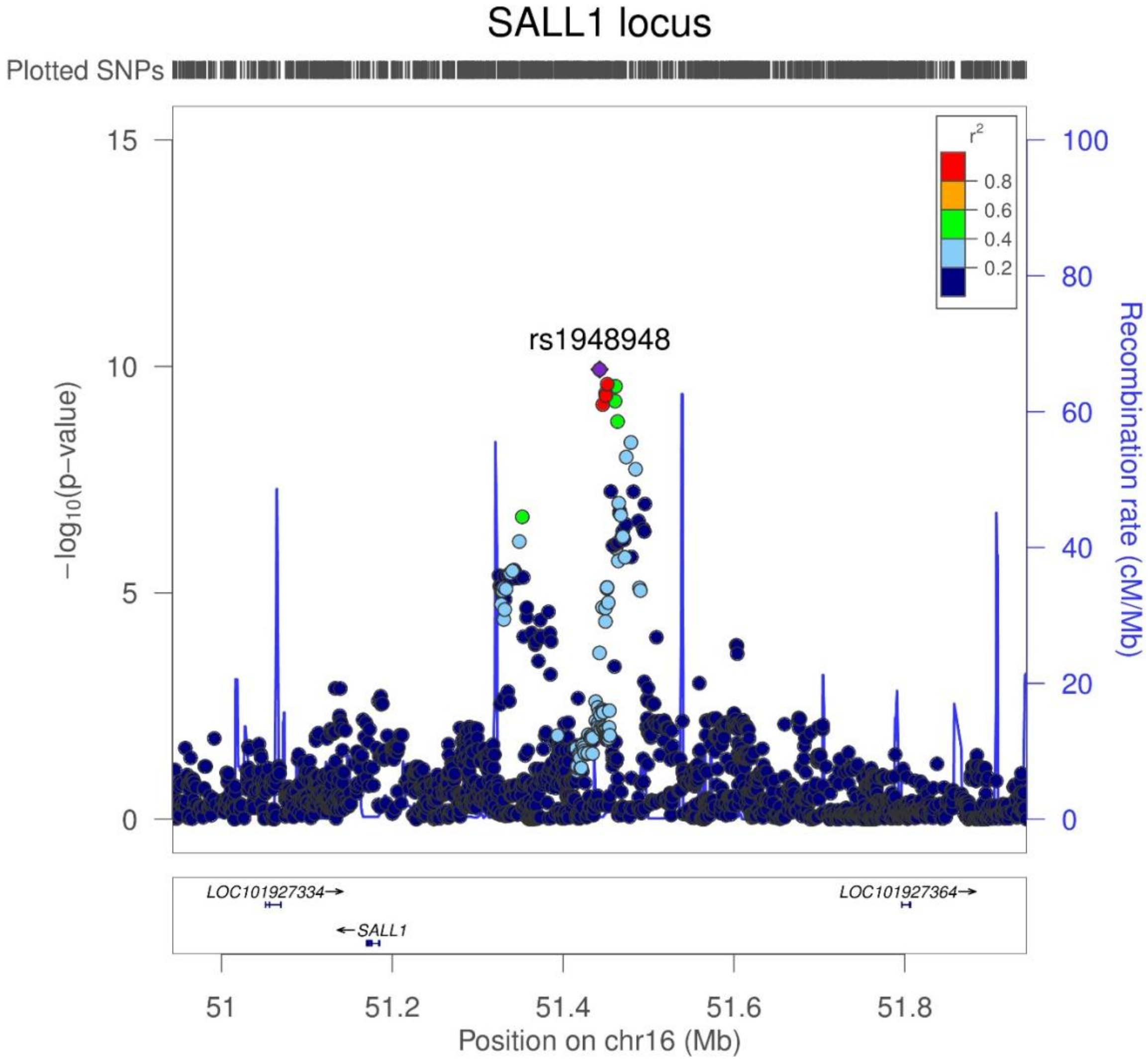
SALL1 locus
Plotted SNPs
15
10
5
0
−log10(p−value)
100
80
60
40
20
0
Recombination rate (cM/Mb)
r2
0.8
0.6
0.4
0.2
rs1948948
LOC101927334→
←SALL1
LOC101927364→
51
51.2
51.4
51.6
51.8
Position on chr16 (Mb)

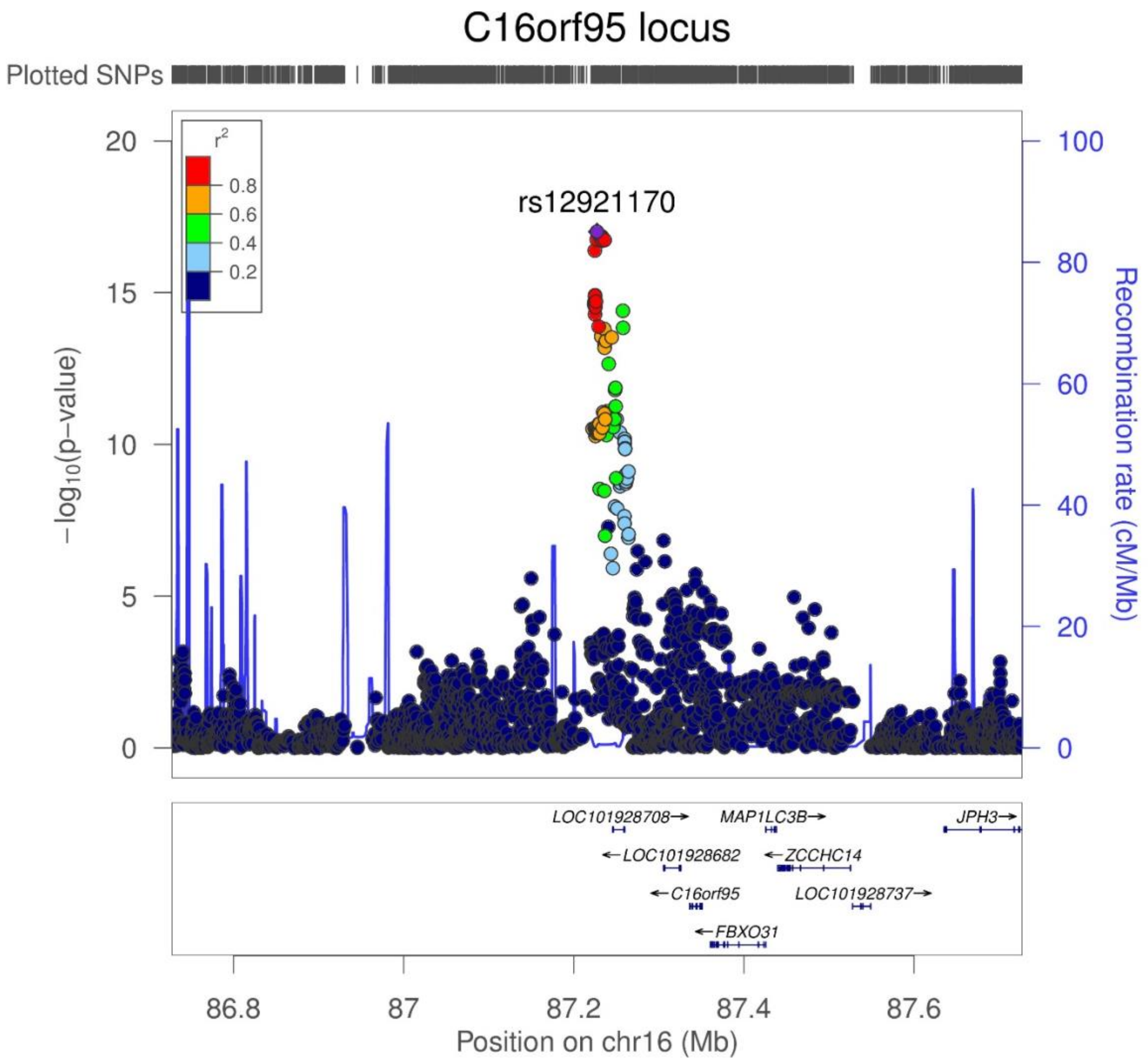
C16orf95 locus
Plotted SNPs
rs12921170
r²
0.8
0.6
0.4
0.2
−log10(p−value)
Recombination rate (cM/Mb)
LOC101928708→
MAP1LC3B→
JPH3→
←LOC101928682
←ZCCHC14
←C16orf95
LOC101928737→
←FBXO31
86.8
87
87.2
87.4
87.6
Position on chr16 (Mb)

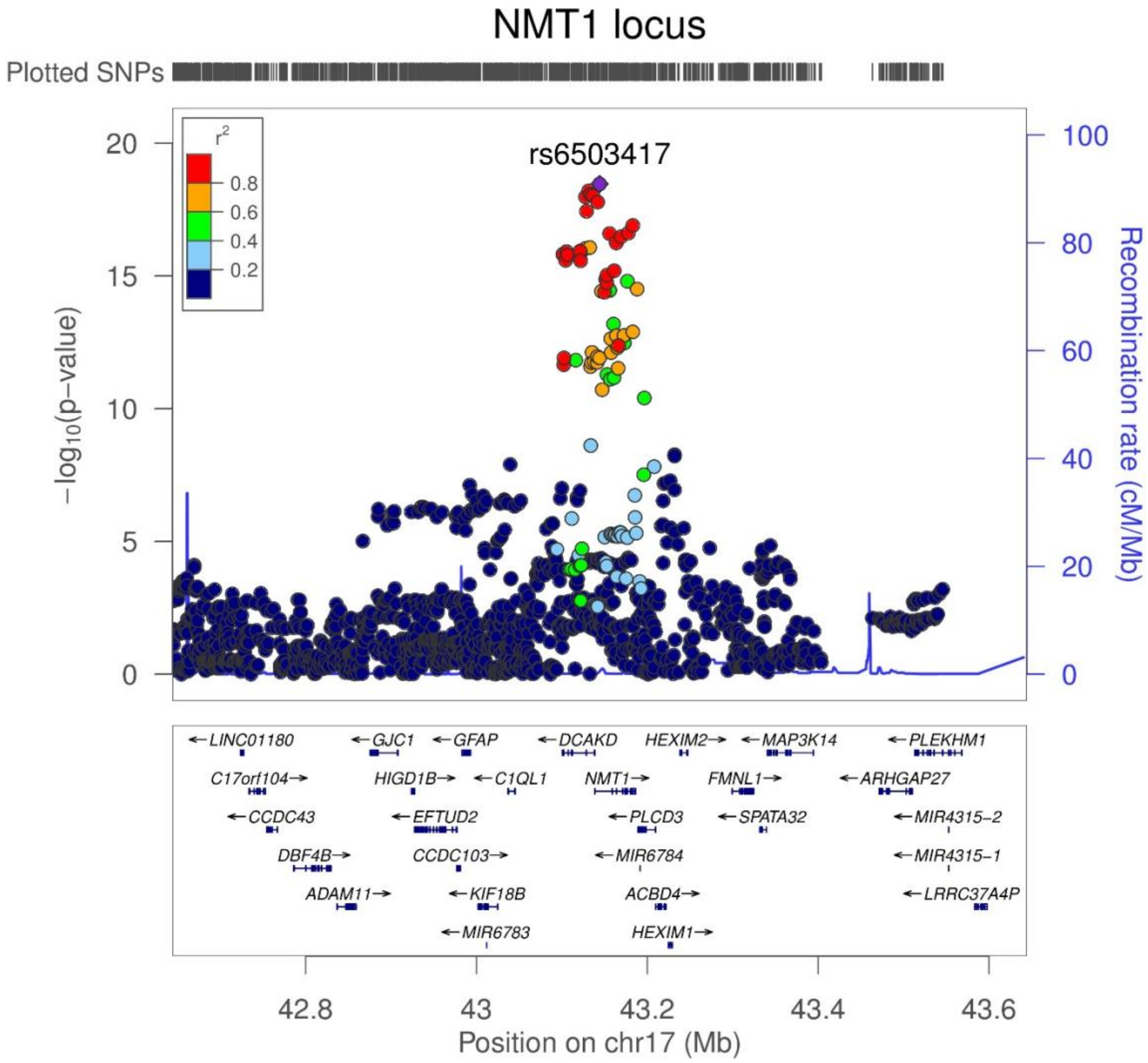
NMT1 locus
Plotted SNPs
rs6503417
r2
0.8
0.6
0.4
0.2
−log10(p−value)
Recombination rate (cM/Mb)
LINC01180
GJC1
GFAP
DCAKD
HEXIM2
MAP3K14
PLEKHM1
C17orf104
HIGD1B
C1QL1
NMT1
FMNL1
ARHGAP27
CCDC43
EFTUD2
PLCD3
SPATA32
MIR4315−2
DBF4B
CCDC103
MIR6784
MIR4315−1
ADAM11
KIF18B
ACBD4
LRRC37A4P
MIR6783
HEXIM1
Position on chr17 (Mb)

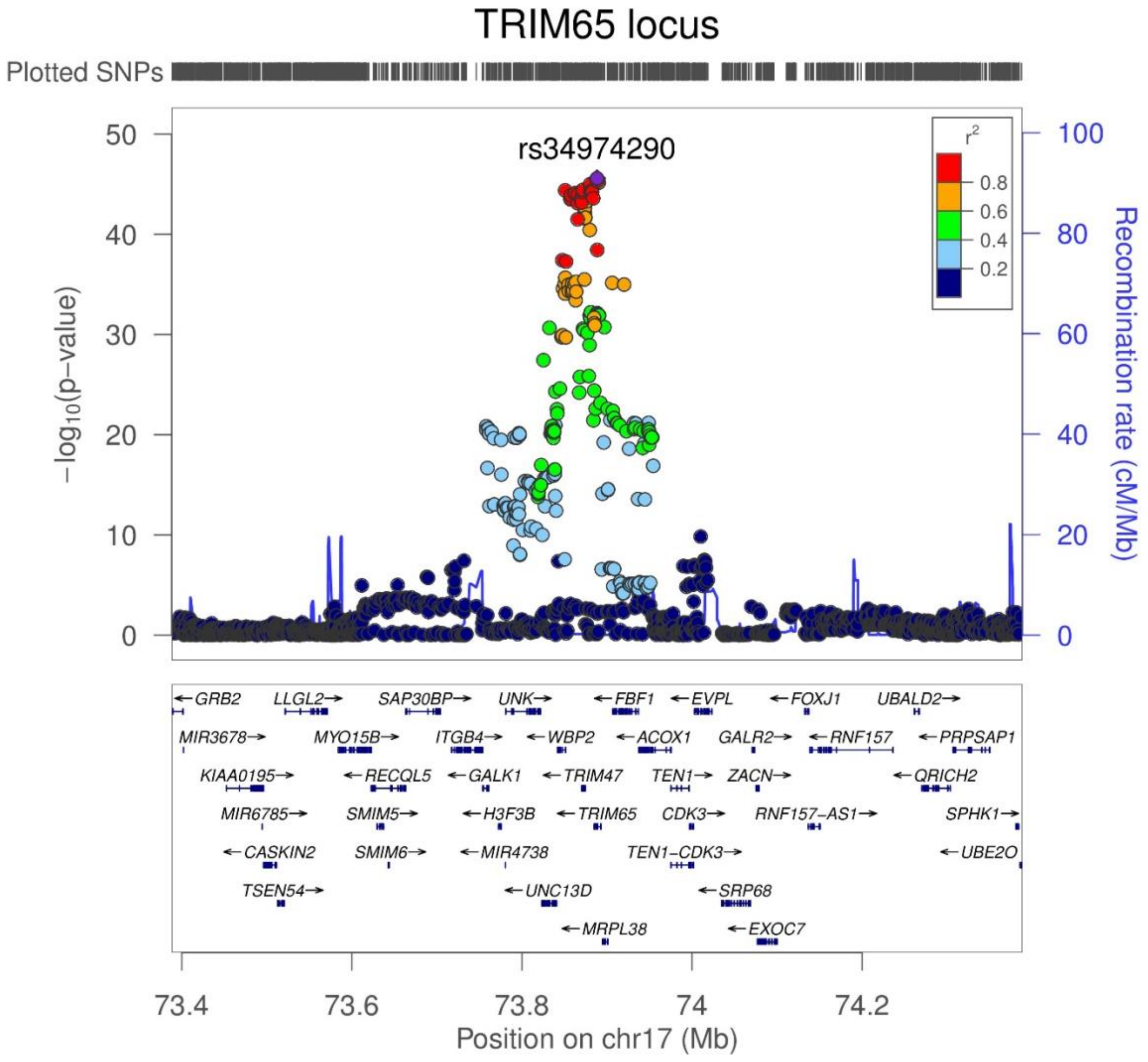

TRIM65 locus
Plotted SNPs
rs34974290
−log10(p−value)
Recombination rate (cM/Mb)
r²
0.8
0.6
0.4
0.2
0
10
20
30
40
50
0
20
40
60
80
100
←GRB2
LLGL2→
SAP30BP→
UNK→
←FBF1
←EVPL
←FOXJ1
UBALD2→
MIR3678→
MYO15B→
ITGB4→
←WBP2
←ACOX1
GALR2→
←RNF157
←PRPSAP1
KIAA0195→
←RECQL5
←GALK1
←TRIM47
TEN1→
ZACN→
←QRICH2
MIR6785→
SMIM5→
←H3F3B
←TRIM65
CDK3→
RNF157−AS1→
SPHK1→
←CASKIN2
SMIM6→
←MIR4738
TEN1−CDK3→
←UBE2O
TSEN54→
←UNC13D
←SRP68
←MRPL38
←EXOC7
73.4
73.6
73.8
74
74.2
Position on chr17 (Mb)

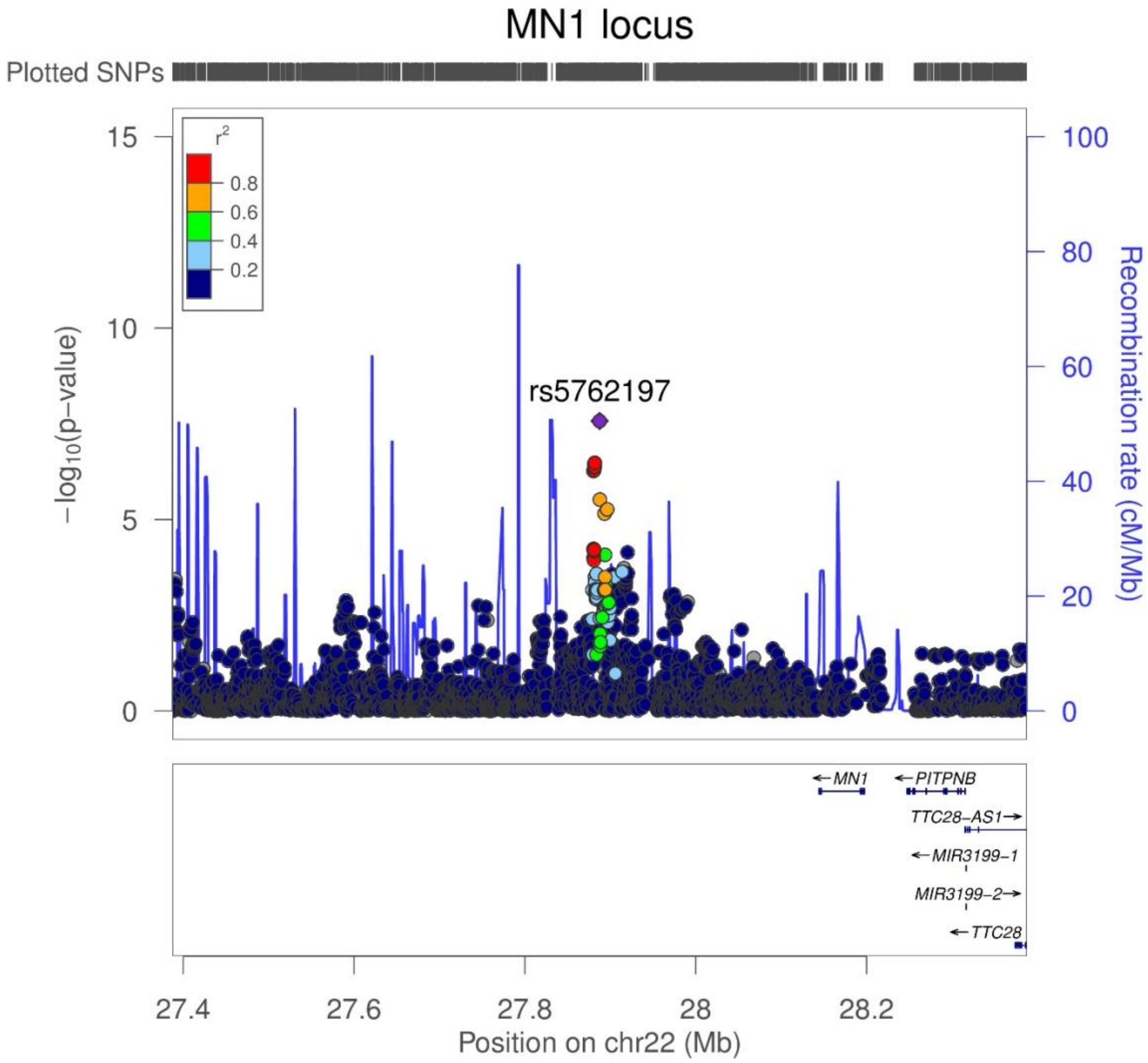
MN1 locus
Plotted SNPs
r²
0.8
0.6
0.4
0.2
rs5762197
−log10(p−value)
Recombination rate (cM/Mb)
MN1
PITPNB
TTC28−AS1
MIR3199−1
MIR3199−2
TTC28
27.4
27.6
27.8
28
28.2
Position on chr22 (Mb)

Supplementary Figure 4: Forest plot showing the distribution of HTN status interaction effects on WMH risk

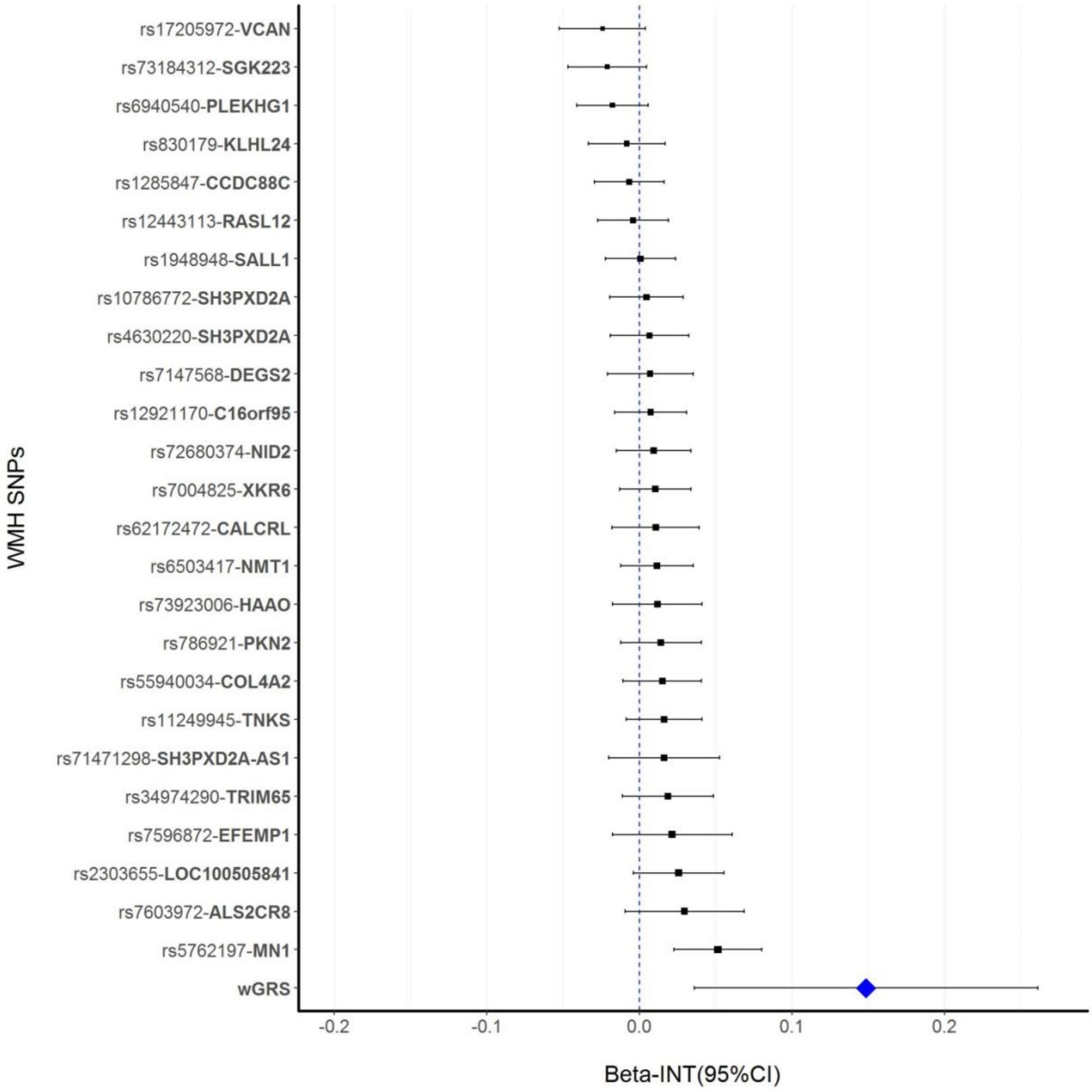

Forest plot displaying 1-degree of freedom SNP by hypertension (HTN) interaction effect estimates (βint) and 95% confidence intervals in relation with WMH burden, for each individual lead SNP of genome-wide significant loci plotted by decreasing p-values and for the WMH weighted genetic risk score (combining these lead SNPs weighted by main effect estimates on WMH) at the bottom (blue diamond)

Supplementary references

1 Harris, T. B. *et al.* Age, Gene/Environment Susceptibility-Reykjavik Study: multidisciplinary applied phenomics. *Am J Epidemiol* **165**, 1076-1087, doi:10.1093/aje/kwk115 (2007).

2 Vidal, J. S. *et al.* Coronary artery calcium, brain function and structure: the AGES-Reykjavik Study. *Stroke* **41**, 891-897, doi:10.1161/strokeaha.110.579581 (2010).

3 The Atherosclerosis Risk in Communities (ARIC) Study: design and objectives. The ARIC investigators. *Am J Epidemiol* **129**, 687-702 (1989).

4 Schmidt, R. *et al.* Assessment of cerebrovascular risk profiles in healthy persons: definition of research goals and the Austrian Stroke Prevention Study (ASPS). *Neuroepidemiology* **13**, 308-313, doi:10.1159/000110396 (1994).

5 Schmidt, R., Fazekas, F., Kapeller, P., Schmidt, H. & Hartung, H. P. MRI white matter hyperintensities: three-year follow-up of the Austrian Stroke Prevention Study. *Neurology* **53**, 132-139 (1999).

6 Seiler, S. *et al.* Magnetization transfer ratio relates to cognitive impairment in normal elderly. *Front Aging Neurosci* **6**, 263, doi:10.3389/fnagi.2014.00263 (2014).

7 Ghadery, C. *et al.* R2* mapping for brain iron: associations with cognition in normal aging. *Neurobiol Aging* **36**, 925-932, doi:10.1016/j.neurobiolaging.2014.09.013 (2015).

8 Fried, L. P. *et al.* The Cardiovascular Health Study: design and rationale. *Ann Epidemiol* **1**, 263-276 (1991).

9 Lopez, O. L. *et al.* Evaluation of dementia in the cardiovascular health cognition study. *Neuroepidemiology* **22**, 1-12, doi:10.1159/000067110 (2003).

10 Longstreth, W. T., Jr. *et al.* Frequency and predictors of stroke death in 5,888 participants in the Cardiovascular Health Study. *Neurology* **56**, 368-375 (2001).

11 Evans, D. A. *et al.* Incidence of Alzheimer disease in a biracial urban community: relation to apolipoprotein E allele status. *Arch Neurol* **60**, 185-189 (2003).

12 Friedman, G. D. *et al.* CARDIA: study design, recruitment, and some characteristics of the examined subjects. *J Clin Epidemiol* **41**, 1105-1116 (1988).

13 Dawber, T. R. & Kannel, W. B. The Framingham study. An epidemiological approach to coronary heart disease. *Circulation* **34**, 553-555 (1966).

14 Feinleib, M., Kannel, W. B., Garrison, R. J., McNamara, P. M. & Castelli, W. P. The Framingham Offspring Study. Design and preliminary data. *Prev Med* **4**, 518-525 (1975).

15 Splansky, G. L. *et al.* The Third Generation Cohort of the National Heart, Lung, and Blood Institute's Framingham Heart Study: design, recruitment, and initial examination. *Am J Epidemiol* **165**, 1328-1335, doi:10.1093/aje/kwm021 (2007).

16 Carandang, R. *et al.* Trends in incidence, lifetime risk, severity, and 30-day mortality of stroke over the past 50 years. *JAMA* **296**, 2939-2946, doi:10.1001/jama.296.24.2939 (2006).

17 Seshadri, S. *et al.* The lifetime risk of stroke: estimates from the Framingham Study. *Stroke* **37**, 345-350, doi:10.1161/01.STR.0000199613.38911.b2 (2006).

18 Multi-center genetic study of hypertension: The Family Blood Pressure Program (FBPP). *Hypertension* **39**, 3-9 (2002).

19 Daniels, P. R. *et al.* Familial aggregation of hypertension treatment and control in the Genetic Epidemiology Network of Arteriopathy (GENOA) study. *Am J Med* **116**, 676-681, doi:10.1016/j.amjmed.2003.12.032 (2004).

20 Vaidya, D. *et al.* Incidence of coronary artery disease in siblings of patients with premature coronary artery disease: 10 years of follow-up. *Am J Cardiol* **100**, 1410-1415, doi:10.1016/j.amjcard.2007.06.031 (2007).

21 Nyquist, P. A. *et al.* Age differences in periventricular and deep white matter lesions. *Neurobiol Aging* **36**, 1653-1658, doi:10.1016/j.neurobiolaging.2015.01.005 (2015).

22 Schoenmaker, M. *et al.* Evidence of genetic enrichment for exceptional survival using a family approach: the Leiden Longevity Study. *Eur J Hum Genet* **14**, 79-84, doi:10.1038/sj.ejhg.5201508 (2006).

23 Wardlaw, J. M. *et al.* Brain aging, cognition in youth and old age and vascular disease in the Lothian Birth Cohort 1936: rationale, design and methodology of the imaging protocol. *Int J Stroke* **6**, 547-559, doi:10.1111/j.1747-4949.2011.00683.x (2011).
24 Deary, I. J. *et al.* The Lothian Birth Cohort 1936: a study to examine influences on cognitive ageing from age 11 to age 70 and beyond. *BMC Geriatr* **7**, 28, doi:10.1186/1471-2318-7-28 (2007).
25 Sachdev, P. S. *et al.* The Sydney Memory and Ageing Study (MAS): methodology and baseline medical and neuropsychiatric characteristics of an elderly epidemiological non-demented cohort of Australians aged 70-90 years. *Int Psychogeriatr* **22**, 1248-1264, doi:10.1017/s1041610210001067 (2010).
26 Sachdev, P. S. *et al.* A comprehensive neuropsychiatric study of elderly twins: the Older Australian Twins Study. *Twin Res Hum Genet* **12**, 573-582, doi:10.1375/twin.12.6.573 (2009).
27 Sachdev, P. S. *et al.* Cognitive functioning in older twins: the Older Australian Twins Study. *Australas J Ageing* **30 Suppl 2**, 17-23, doi:10.1111/j.1741-6612.2011.00534.x (2011).
28 Shepherd, J. *et al.* The design of a prospective study of Pravastatin in the Elderly at Risk (PROSPER). PROSPER Study Group. PROspective Study of Pravastatin in the Elderly at Risk. *Am J Cardiol* **84**, 1192-1197 (1999).
29 Shepherd, J. *et al.* Pravastatin in elderly individuals at risk of vascular disease (PROSPER): a randomised controlled trial. *Lancet* **360**, 1623-1630 (2002).
30 Ikram, M. A. *et al.* The Rotterdam Study: 2018 update on objectives, design and main results. *Eur J Epidemiol* **32**, 807-850, doi:10.1007/s10654-017-0321-4 (2017).
31 Ikram, M. A. *et al.* The Rotterdam Scan Study: design update 2016 and main findings. *Eur J Epidemiol* **30**, 1299-1315, doi:10.1007/s10654-015-0105-7 (2015).
32 Volzke, H. *et al.* Cohort profile: the study of health in Pomerania. *Int J Epidemiol* **40**, 294-307, doi:10.1093/ije/dyp394 (2011).
33 Hegenscheid, K. *et al.* Whole-body magnetic resonance imaging of healthy volunteers: pilot study results from the population-based SHIP study. *Rofo* **181**, 748-759, doi:10.1055/s-0028-1109510 (2009).
34 Callisaya, M. L. *et al.* A population-based study of sensorimotor factors affecting gait in older people. *Age Ageing* **38**, 290-295, doi:10.1093/ageing/afp017 (2009).
35 Vascular factors and risk of dementia: design of the Three-City Study and baseline characteristics of the study population. *Neuroepidemiology* **22**, 316-325, doi:10.1159/000072920 (2003).
36 Godin, O. *et al.* White matter lesions as a predictor of depression in the elderly: the 3C-Dijon study. *Biol Psychiatry* **63**, 663-669, doi:10.1016/j.biopsych.2007.09.006 (2008).
37 Soumare, A. *et al.* White matter lesions volume and motor performances in the elderly. *Ann Neurol* **65**, 706-715, doi:10.1002/ana.21674 (2009).
38 Maillard, P. *et al.* An automated procedure for the assessment of white matter hyperintensities by multispectral (T1, T2, PD) MRI and an evaluation of its between-centre reproducibility based on two large community databases. *Neuroradiology* **50**, 31-42, doi:10.1007/s00234-007-0312-3 (2008).
39 Zijdenbos, A. P., Forghani, R. & Evans, A. C. Automatic "pipeline" analysis of 3-D MRI data for clinical trials: application to multiple sclerosis. *IEEE Trans Med Imaging* **21**, 1280-1291, doi:10.1109/tmi.2002.806283 (2002).
40 Sigurdsson, S. *et al.* Brain tissue volumes in the general population of the elderly: the AGES-Reykjavik study. *Neuroimage* **59**, 3862-3870, doi:10.1016/j.neuroimage.2011.11.024 (2012).
41 Howard, G. *et al.* Cigarette smoking and other risk factors for silent cerebral infarction in the general population. *Stroke* **29**, 913-917 (1998).
42 Bryan, R. N. *et al.* A method for using MR to evaluate the effects of cardiovascular disease on the brain: the cardiovascular health study. *AJNR Am J Neuroradiol* **15**, 1625-1633 (1994).

43 Schmidt, R. *et al.* C-reactive protein, carotid atherosclerosis, and cerebral small-vessel disease: results of the Austrian Stroke Prevention Study. *Stroke* **37**, 2910-2916, doi:10.1161/01.STR.0000248768.40043.f9 (2006).
44 Fletcher, E., Singh, B., Harvey, D., Carmichael, O. & DeCarli, C. Adaptive image segmentation for robust measurement of longitudinal brain tissue change. *Conf Proc IEEE Eng Med Biol Soc* **2012**, 5319-5322, doi:10.1109/embc.2012.6347195 (2012).
45 Desikan, R. S. *et al.* An automated labeling system for subdividing the human cerebral cortex on MRI scans into gyral based regions of interest. *Neuroimage* **31**, 968-980, doi:10.1016/j.neuroimage.2006.01.021 (2006).
46 Lee, D. Y. *et al.* Vascular and degenerative processes differentially affect regional interhemispheric connections in normal aging, mild cognitive impairment, and Alzheimer disease. *Stroke* **41**, 1791-1797, doi:10.1161/strokeaha.110.582163 (2010).
47 Das, S. R., Avants, B. B., Grossman, M. & Gee, J. C. Registration based cortical thickness measurement. *Neuroimage* **45**, 867-879, doi:10.1016/j.neuroimage.2008.12.016 (2009).
48 Lao, Z. *et al.* Computer-assisted segmentation of white matter lesions in 3D MR images using support vector machine. *Acad Radiol* **15**, 300-313, doi:10.1016/j.acra.2007.10.012 (2008).
49 Jack, C. R., Jr. *et al.* FLAIR histogram segmentation for measurement of leukoaraiosis volume. *J Magn Reson Imaging* **14**, 668-676 (2001).
50 Smith, S. M. *et al.* Advances in functional and structural MR image analysis and implementation as FSL. *Neuroimage* **23 Suppl 1**, S208-219, doi:10.1016/j.neuroimage.2004.07.051 (2004).
51 Smith, S. M. *et al.* Tract-based spatial statistics: voxelwise analysis of multi-subject diffusion data. *Neuroimage* **31**, 1487-1505, doi:10.1016/j.neuroimage.2006.02.024 (2006).
52 Baykara, E. *et al.* A Novel Imaging Marker for Small Vessel Disease Based on Skeletonization of White Matter Tracts and Diffusion Histograms. *Ann Neurol* **80**, 581-592, doi:10.1002/ana.24758 (2016).
53 Lopez, L. M. *et al.* Genes from a translational analysis support a multifactorial nature of white matter hyperintensities. *Stroke* **46**, 341-347, doi:10.1161/strokeaha.114.007649 (2015).
54 Jiang, J. *et al.* UBO Detector - A cluster-based, fully automated pipeline for extracting white matter hyperintensities. *Neuroimage* **174**, 539-549, doi:10.1016/j.neuroimage.2018.03.050 (2018).
55 Admiraal-Behloul, F. *et al.* Fully automatic segmentation of white matter hyperintensities in MR images of the elderly. *Neuroimage* **28**, 607-617, doi:10.1016/j.neuroimage.2005.06.061 (2005).
56 Ikram, M. A. *et al.* Brain tissue volumes in the general elderly population. The Rotterdam Scan Study. *Neurobiol Aging* **29**, 882-890, doi:10.1016/j.neurobiolaging.2006.12.012 (2008).
57 Griffanti, L. *et al.* BIANCA (Brain Intensity AbNormality Classification Algorithm): A new tool for automated segmentation of white matter hyperintensities. *Neuroimage* **141**, 191-205, doi:10.1016/j.neuroimage.2016.07.018 (2016).
58 Evangelou, E. *et al.* Genetic analysis of over 1 million people identifies 535 new loci associated with blood pressure traits. *Nat Genet* **50**, 1412-1425, doi:10.1038/s41588-018-0205-x (2018).
59 Willer, C. J. *et al.* Discovery and refinement of loci associated with lipid levels. *Nat Genet* **45**, 1274-1283, doi:10.1038/ng.2797 (2013).
60 Xue, A. *et al.* Genome-wide association analyses identify 143 risk variants and putative regulatory mechanisms for type 2 diabetes. *Nat Commun* **9**, 2941, doi:10.1038/s41467-018-04951-w (2018).
61 Germain, M. *et al.* Meta-analysis of 65,734 individuals identifies TSPAN15 and SLC44A2 as two susceptibility loci for venous thromboembolism. *American journal of human genetics* **96**, 532-542 (2015).
62 Gormley, P. *et al.* Meta-analysis of 375,000 individuals identifies 38 susceptibility loci for migraine. *Nat Genet* **48**, 856-866, doi:10.1038/ng.3598 (2016).

63 Malik, R. *et al.* Multiancestry genome-wide association study of 520,000 subjects identifies 32 loci associated with stroke and stroke subtypes. *Nat Genet* **50**, 524-537, doi:10.1038/s41588-018-0058-3 (2018).
64 Woo, D. *et al.* Meta-analysis of genome-wide association studies identifies 1q22 as a susceptibility locus for intracerebral hemorrhage. *Am J Hum Genet* **94**, 511-521, doi:10.1016/j.ajhg.2014.02.012 (2014).
65 Davies, G. *et al.* Study of 300,486 individuals identifies 148 independent genetic loci influencing general cognitive function. *Nat Commun* **9**, 2098, doi:10.1038/s41467-018-04362-x (2018).
66 Jansen, I. E. *et al.* Genome-wide meta-analysis identifies new loci and functional pathways influencing Alzheimer's disease risk. *Nat Genet* **51**, 404-413, doi:10.1038/s41588-018-0311-9 (2019).
67 Yang, J., Zaitlen, N. A., Goddard, M. E., Visscher, P. M. & Price, A. L. Advantages and pitfalls in the application of mixed-model association methods. *Nat Genet* **46**, 100-106, doi:10.1038/ng.2876 (2014).
68 Naba, A. *et al.* The matrisome: in silico definition and in vivo characterization by proteomics of normal and tumor extracellular matrices. *Mol Cell Proteomics* **11**, M111 014647, doi:10.1074/mcp.M111.014647 (2012).
