# Supplementary material for: Cerebral small vessel disease genomics and its implications across the lifespan
Source: Nat Commun. 2020 Dec 8;11:6285. doi: 10.1038/s41467-020-19111-2 (PMC7722866; doi:10.1038/s41467-020-19111-2)
Supplement: Supplementary file 3 — Description of Additional Supplementary Files [file 41467_2020_19111_MOESM3_ESM.pdf]

Descriptions of Additional Supplementary Files

Supplementary Data 1. Study design and sample characteristics of the individual studies

Description: MRI = Magnetic Resonance Imaging; WMH = White Matter Hyperintensities; BP = Blood pressure; PP = Pulse Pressure; PC = Principal Component; ICV = Intracranial Volume; 3C-Dijon = Three-City Dijon Study; AGES = AGES-Reykjavik Study; ARIC = Atherosclerosis Risk In Communities Study; AA = African American; EUR = European Ancestry; ASPS = Austrian Stroke Prevention Study; ASPS-Fam = Austrian Stroke Prevention Family Study; CARDIA = Coronary Artery Risk Development in Young Adults; CHAP = Chicago Health and Aging Project; CHS = Cardiovascular Health Study; FHS = Framingham Heart Study; GeneSTAR = Genetic Studies of Atherosclerosis Risk; GENOA = Genetic Epidemiology Network of Arteriopathy; LBC1936 = Lothian Birth Cohort 1936; LLS = Leiden Longevity Study; OATS = Older Australian Twins Study; PROSPER = The Prospective Study on Pravastatin in the Elderly at Risk; RS = Rotterdam Study; SHIP/SHIP-TREND = Study of Health in Pomerania; Sydney MAS = Sydney Memory and Ageing Study; TASCOG = Tasmanian Study of Cognition and Gait; UKBB = UK BioBank. * Pathologies that may influence WMH measurement.† Grade (0-9 scale). ‡ with information on hypertension status. § Stroke or dementia

Supplementary Data 2. GWAS genotyping platforms, imputation panels and quality control of genotypes

Description: SNP = Single Nucleotide Polymorphism; PC = Principal Component; TIV = Total Intracranial volume; ICV = Intracranial Volume; 3C-Dijon = Three-City Dijon Study; AGES = AGES-Reykjavik Study; ARIC = Atherosclerosis Risk In Communities Study; ASPS = Austrian Stroke Prevention Study; ASPS-Fam: Austrian Stroke Prevention Family Study; CARDIA = Coronary Artery Risk Development in Young Adults; CHAP = Chicago Health and Aging Project; CHS = Cardiovascular Health Study; FHS = Framingham Heart Study; GeneSTAR = Genetic Studies of Atherosclerosis Risk; GENOA = Genetic Epidemiology Network of Arteriopathy; LBC1936 = Lothian Birth Cohort 1936; LLS = Leiden Longevity Study; OATS = Older Australian Twins Study; PROSPER = The Prospective Study on Pravastatin in the Elderly at Risk; RS = Rotterdam Study; SHIP/SHIP-TREND = Study of Health in Pomerania; Sydney MAS = Sydney Memory and Ageing Study; TASCOG = Tasmanian Study of Cognition and Gait; UKBB = UK BioBank. *Affymetrix SNP 6.0. † Illumina Omni 2.5

Supplementary Data 3. Genomic-control inflation factor (AS, lambda) for individual studies and meta-analysis

Description: HTN = Hypertension; JMA = Joint Meta-Analysis; EUR = European Ancestry; AA = African-American. * Main effects are assessed in Model 1 adjusted for age, sex, principal components for population stratification, total intracranial volume, and in Model 2 (Model 1 + hypertension)

Supplementary Data 4. Cross-ancestry comparison of GW associated White Matter Hyperintensities loci

Description: GW= Genome-Wide; Meta-Analysis EUR = Meta-Analysis in European Ancestry; Meta-Analysis AA = Meta-Analysis in African-American;

SNP = Single Nucleotide Polymorphism; EA = effect allele; OA = Other Allele; 13 = effect estimate; SE = Standard Error; P = P-value; HTN = Hypertension.* Main effects are assessed in Model 1 adjusted for age, sex, principal components for population stratification, total intracranial volume, and in Model 2 (Model 1 + hypertension) . † Additional locus reaching GW significance in the GCTA-COJO analysis for the main effects model, 13j=joint effect, SE_13j=Standard error of 13j, Pj=Joint P-value. ‡ Additional locus reaching GW significance in the African-American only meta-analysis. § Additional locus reaching GW significance in the MR-MEGA multiancestry meta-analysis

Supplementary Data 5. Loci reaching GW significance in the multiancestry meta-analysis for the main effects in association models adjusted for hypertension status or not

Description: GW= Genome-Wide; HTN= Hypertension; SNP = Single Nucleotide Polymorphism; EA = Effect Allele; OA = Other Allele; EAF = Effect Allele Frequency; 13 = effect estimate; SE = Standard Error; P = P-value; P.MRMega = P-value MR-MEGA analysis; PHet-ANC = heterogeneity P-value due to ancestry; PHet-RES = residua

heterogeneity P-value. * Main effects are assessed in Model 1 adjusted for age, sex, principal components for population stratification, total intracranial volume, and in Model 2 (Model 1 + hypertension). ‡ Additional locus reaching GW significance in the MR-MEGA multiancestry meta-analysis

Supplementary Data 6. Loci reaching GW significance in the multiancestry meta-analysis for the joint meta-analysis (JMA) model

Description: GW = Genome-Wide; JMA = Joint Meta-Analysis; HTN= Hypertension; Chr = chromosome; SNP = Single Nucleotide Polymorphism; EA = Effect Allele; OA = Other Allele; EAF = Effect Allele Frequency; 13 = effect estimate; SE = Standard Error; P = P-value. * Main effects are assessed in Model 1 adjusted for age, sex, principal components for population stratification, total intracranial volume, and in Model 2 (Model 1 + hypertension)

Supplementary Data 7. Loci reaching GW significance for the joint meta-analysis (JMA) in African-American specific analysis

Description: GW = Genome-Wide; JMA = joint meta-analysis; EUR = European ancestry; AA = African-American; SNP = Single Nucleotide Polymorphism; EA = Effect Allele; OA = Other Allele; EAF = Effect Allele Frequency; P = P-value

Supplementary Data 8. Association status of GW associated White Matter Hyperintensity (WMH) loci with WMH burden stratified by hypertension status

Description: GW = Genome-Wide; HTN = Hypertension; SNP = Single Nucleotide Polymorphism; EA = Effect Allele; OA = Other Allele; EAF = Effect Allele Frequency; 13 = effect estimate; SE = Standard Error; P = P-value; Phet = heterogeneity P-value; ncRNA = non coding RNA. * Additional locus reaching GW significance in the GCTA-COJO analysis for the main effects model. † Locus reaching Genome-Wide significance in African-American specific analysis

Supplementary Data 9. Association of GW significant WMH SNPs and WMH wGRS with WMH values in the UK biobank stratified by SBP GRS distribution (quartiles)

Description: GW= Genome-wide; Chr = chromosome; SNP = Single Nucleotide Polymorphism; EA = Effect Allele; OA = Other Allele; EAF = Effect Allele Frequency; 13 = effect estimate; SE = Standard Error; P = P-value; GRS = genetic risk score; wGRS= weighted GRS. Linear regression: adjusted for age, sex, principal components for population stratification, total intracranial volume

Supplementary Data 10. Association of GW significant WMH SNPs and WMH wGRS with WMH values in the UK biobank stratified by DBP GRS distribution (quartiles)

Description: GW= Genome-wide; Chr = chromosome; SNP = Single Nucleotide Polymorphism; EA = Effect Allele; OA = Other Allele; EAF = Effect Allele Frequency; 13 = effect estimate; SE = Standard Error; P = P-value; GRS = genetic risk score; wGRS= weighted GRS. Linear regression: adjusted for age, sex, principal components for population stratification, total intracranial volume

Supplementary Data 11. Suggestive associations of genetic loci with White Matter Hyperintensity (WMH) volume at 5x10-8<p<5x10-6

Description: JMA = Joint Meta-Analysis; EUR= European ancestry; SNP = Single Nucleotide Polymorphism; EA = Effect Allele; OA = Other Allele; EAF = Effect Allele Frequency; 13 = effect estimate; SE = Standard Error; P = P-value. * Main effects are assessed in Model 1 adjusted for age, sex, principal components for population stratification, total intracranial volume, and in Model 2 (Model 1 + hypertension)

Supplementary Data 12. Loci reaching gene-wide significance (P-value < 2.77 x10-6) from the MAGMA analysis

Description: Chr = chromosome; NSNPS = number of SNPs; N = sample size; ZSTAT = Z-value effect size for the gene; P = P-value. * Association status based on the proximity to the associated White Matter Hyperintensities loci (see online methods)

Supplementary Data 13. Association of genome-wide significant White Matter Hyperintensity (WMH) loci with related vascular and neurological traits

Description: GW= Genome Wide; SNP = Single Nucleotide Polymorphism; EA = Effect Allele; Chr = chromosome; P = P-value; SBP = Systolic Blood Pressure; PP = Pulse Pressure; DBP = Diastolic Blood Pressure; SMKindex = lifetime smoking index; BMI = Body Mass Index; LDL = Low-Density Lipoprotein; T2D = Type II Diabetes; AS = All Stroke; SVS = Small Vessel Stroke; IS = Ischemic Stroke; CES = Cardio-Embolic stroke; AD = Alzheimer's Disease; ICH = Intracerebral Hemorrhage; VTE = Venous Thrombo Embolism. * Linkage disequilibrium (LD) between the lead WMH SNP and the overlapping SNP. † Effect estimate corresponding to the WMH risk increasing allele

Supplementary Data 14. LD score regression (LDSR) estimates of the genome-wide genetic correlation between White Matter Hyperintensities (WMH) and related vascular and neurological traits

Description: 13 = effect estimate; SE = Standard Error; P = P-value; AS = All Stroke; IS = Ischemic Stroke; DBP = Diastolic Blood Pressure; BMI = Body Mass Index; SBP = Systolic Blood Pressure; SMKindex = lifetime smoking index; SVS = Small Vessel Stroke; GCF = General Cognitive Function; VTE = Venous Thrombo Embolism; T2D = Type II Diabetes; ICH = Intracerebral Hemorrhage; PP = Pulse Pressure; CE = Cardio-Embolic stroke; AD = Alzheimer's Disease; LAS = Large Artery Stroke; LDL = Low-Density Lipoprotein; HDL = High-Density Lipoprotein; TG = triglycerides

Supplementary Data 15. Regional genetic overlap between White Matter Hyperintensity (WMH) and related vascular and neurological traits with high probability (> 90%), using a Bayesian pairwise GWAS approach

Description: GWAS-PW = GWAS pairwise analysis; ρHESS = rho heritability estimator from summary statistics; Chr = Chromosome; PPA3 = posterior probability of association for model 3; ZSTAT = Z-value effect size; P = P-value; AS = All Stroke; BMI = Body Mass Index; CE = Cardio-Embolic stroke; DBP = Diastolic Blood Pressure; GCF = General Cognitive Function; HDL = High-Density Lipoprotein; IS = Ischemic Stroke; LDL = Low-Density Lipoprotein; PP = Pulse Pressure; SBP = Systolic Blood Pressure; SVS = Small Vessel Stroke; SMKindex = lifetime smoking index. * Nearest gene to the top associated WMH SNP from this region

Supplementary Data 16. Association between white matter hyperintensity (WMH) and neurological traits using mendelian randomization (MR) experiments

Description: 13 = effect estimate; SE = standard error; IVW = inverse variance weighted; P = P-value; Q-PHet = heterogeneity P-value from cochran Q statistic; MR = Mendelian Randomization; QR = Ruckers Q - fitness of model; SVS = Small Vessel Stroke; AS = All Stroke; IS = Ischemic Stroke; AD = Alzheimer's Disease; ICH = Intracerebral Hemorrhage; CE = cardio-embolic

stroke; LAS = large artery stroke; GCF = General Cognitive Function. * Main effects are assessed in i) Model 1 adjusted for age, sex, the first 4 principal components for population stratification, and total intracranial volume

Supplementary Data 17. Association between vascular risk factors and white matter hyperintensity (WMH) using mendelian randomization (MR) experiments

Description: 13 = effect estimate; SE = standard error; IVW = inverse variance weighted; P = P-value; Q-PHet = heterogeneity P-value from Cochran Q statistic; MR = Mendelian Randomization; QR = Ruckers Q - fitness of model; DBP = Diastolic Blood Pressure; HTN = Hypertension; NT = Normotensive; SBP = Systolic Blood Pressure; PP = Pulse Pressure; BMI = Body Mass Index; T2D = Type II Diabetes; LDL = Low-Density lipoprotein; HDL = High-Density Lipoprotein; VTE = Venous Thrombo Embolism; TG = Triglycerides; Hb1Ac = glycated hemoglobin levels; SMKindex = lifetime smoking index. * Main effects are assessed in i) Model 1 adjusted for age, sex, the first 4 principal components for population stratification, and total intracranial volume; ii) Model 2 (Model 1 + hypertension) ; iii) Model 1 in hypertensive individuals; iv) Model 1 in Normotensive individuals

Supplementary Data 18. Association between vascular risk factors (non-pleiotropic BP instruments) and white matter hyperintensity (WMH) using mendelian randomization (MR) experiments

Description: 13 = effect estimate; SE = standard error; IVW = inverse variance weighted; P = P-value; Q-PHet = heterogeneity P-value from Cochran Q statistic; MR = Mendelian Randomization; QR = Ruckers Q - fitness of model; DBP = Diastolic Blood Pressure; HTN = Hypertension; NT = Normotensive; SBP = Systolic Blood Pressure; PP = Pulse Pressure. * Main effects are assessed in i) Model 1 adjusted for age, sex, the first 4 principal components for population stratification, and total intracranial volume; ii) Model 2 (Model 1 + hypertension) ; iii) Model 1 in hypertensive individuals; iv) Model 1 in Normotensive individuals

Supplementary Data 19. Tissue specific enrichment of White Matter Hyperintensity (WMH) risk loci using EPIGWAS and regulatory marks for promoter (H3K4me3) and enhancer (H3K4me1) binding

Description: BP = Blood Pressure; P= P-value. * EPIGWAS association using summary statistics from 25 European-only WMH risk loci. † EPIGWAS association using summary statistics from European-only WMH risk loci that are not shared with any of the blood pressure traits

Supplementary Data 20. Tissue specificity from MAGMA gene-property analysis on the tissue specific gene sets (top 10%) using Karolinska mouse brain single cell RNA (scRNA) data

Description: WMH = White Matter Hyperintensity; BP = Blood Pressure; 13 = effect estimate; SE = Standard Error; P = P-value. * MAGMA association using the full WMH summary statistics from European only main effects analysis. † MAGMA association using the full WMH summary statistics from European only main effects analysis except loci that are harbouring a shared casual variant with any of the blood pressure traits with high posterior probability of association for model 3 (PPA3 > 0.9) from GWAS pairwise analysis

Supplementary Data 21. Transcriptome wide association statistics (TWAS) of White Matter Hyperintensity (WMH) with gene expression from related tissue types

Description: TWAS = transcriptome Wide Association Study; eGENE = expression associated gene; eQTL = SNP associated with gene expression; TWAS.Z = Z-score effect size for TWAS; TWAS.P = TWAS P-value; COLOC.PP4 = Colocalization posterior probability for model 4; P = P-value; BLD = blood, BRN = brain; HRT = heart; BA = brodmann area; DLPFC = dorsolateral prefrontal cortex; CMC = common mind consortium; NTR = netherlands twin

registry; YFS = young finn's study; ROSMAP = religious order study and the rush memory aging project. * WMH P-value for the main effects from European-only analysis. ł Association status of the eQTLs (PP4 >= 0.75) based on the proximity to the associated White Matter Hyperintensities loci (see online methods)

Supplementary Data 22. Drug target enrichment analysis of WMH-TWAS associated genes by Genome for Repositioning drugs (GREP)

Description: ICD10 = International Classification of Diseases 10. * Enrichment for the overall group and the top associated sub-category per group are shown. † Fisher's exact P-value ordered by the significance in the overall group
